# Crystal structure of Al_2.95_Cr_0.59_, a phase closely related to the η-phase in the binary Al–Cr system

**DOI:** 10.1107/S2414314620014121

**Published:** 2020-10-27

**Authors:** Xu Geng, Bin Wen, Changzeng Fan

**Affiliations:** a State Key Laboratory of Metastable Materials Science and Technology, Yanshan University, Qinhuangdao 066004, People’s Republic of China; Vienna University of Technology, Austria

**Keywords:** crystal structure, Al–Cr inter­metallics, high-pressure sinter­ing, icosa­hedra

## Abstract

The monoclinic η-phase in the binary Al–Cr system was initially named by Bradley & Lu [Bradley & Lu (1937 ▸). *J. Inst. Met*. **60**, 319–337] as having the composition Al_11_Cr_2_ (Al:Cr ratio = 5.5:1). Its crystal structure was later refined [Cao & Kuo (2008). *J. Alloys Compd.* **458**, 30, 319–337] to have a slightly lower Al content (Al:Cr ratio = 5.16:1). In the present work, a monoclinic phase with composition Al_2.95_Cr_0.59_ (Al:Cr ratio = 5.04:1) was obtained by high-pressure sinter­ing (HPS) of a stoichiometric Al_11_Cr_2_ mixture. Structure analysis of this phase, hereafter named η′, revealed a close relationship to the previously reported η-Al_11_Cr_2_ structure, but with different mixed-occupied sites. Five fully occupied sites exhibit refined site occupation factors of 0.899 (5), 0.984 (4), 0.977 (5), 0.946 (4) and 0.945 (4) for the corresponding Al atoms. Moreover, there are no split Al sites in the η′-structure as reported for the η-structure. The refined chemical composition of the η′-phase revealed that it comprises two Al atoms fewer and two Cr atoms more than the previously reported η-Al_11_Cr_2_ phase.

## Abstract 2

Al_2.95_Cr_0.59_ was synthesized by high-pressure sinter­ing (HPS). It has a slightly lower Al content than the closely related η-Al_11_Cr_2_ phase.

## Graphical abstract

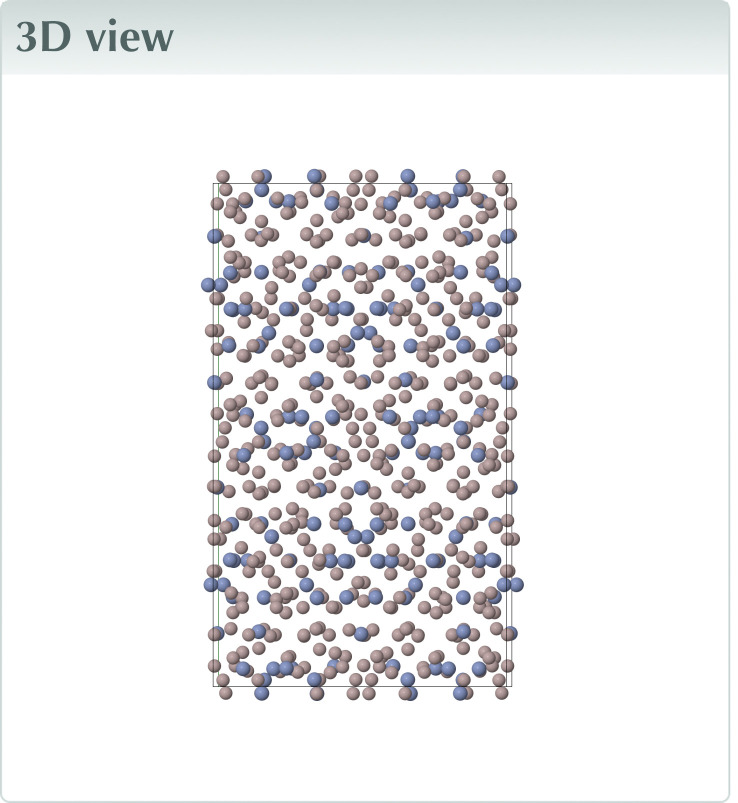

## Structure description

The Al-rich part of the binary Al–Cr system has been investigated intensively in the past. One of the reported phases is monoclinic η-Al_11_Cr_2_ (Al:Cr ratio = 5.5:1), initially named by Bradley & Lu (1937 ▸). Its crystal structure was later refined by Cao & Kuo (2008*a* ▸) to have a slightly lower Al content (Al:Cr ratio = 5.16:1). An additional ortho­rhom­bic phase with similar composition was reported by Little (1954 ▸), but later it was found that this phase represents in fact monoclinic η-Al_11_Cr_2_ (Bendersky *et al.*, 1991 ▸). However, an ortho­rhom­bic phase was prepared by *in situ* heating of the η-Al_11_Cr_2_ phase at 1072 K for 2 h (Cao & Kuo, 2008*b* ▸). The η-Al_11_Cr_2_ phase and the related ortho­rhom­bic phase are crucial for understanding the formation and stability of the Al—Cr icosa­hedral quasicrystal firstly reported by Lilienfeld *et al.* (1986 ▸) shortly after the discovery of quasicrystals in rapidly solidified Al—Mn alloys (Shechtman *et al.*, 1984 ▸). Inoue *et al.* (1987 ▸) found that single-phase icosa­hedral quasicrystals have formed in the vicinity of about 15.4 at.% Cr in rapidly quenched Al–Cr alloys, and the quasicrystal can be approximately formulated to have the composition Al_11_Cr_2_. In terms of thermal stability, the quasicrystal with composition Al_84.6_Cr_15.4_ decomposes into a stable ortho­rhom­bic Al_11_Cr_2_ phase while the quasicrystal containing less Cr (6 to 14.5 at% Cr) changes directly to stable phases of Al + Al_7_Cr and Al_7_Cr + Al_11_Cr_2._ Icosa­hedral quasicrystals from the Al–Cr alloy containing 7 to 15 at.% Cr have twinned Al_7_Cr as final decomposition product while the equilibrium η-Al_11_Cr_2_ phase is completely absent during the decomposition of quasicrystals (Swamy *et al.*, 1989 ▸). Inter­estingly, Zhang *et al.* (1988 ▸) also found rotational twins of the Al_45_Cr_7_ phase while no Al_11_Cr_2_ phase was found in a rapidly solidified Al_7_Cr alloy. From these pioneering studies one can conclude that the Al_11_Cr_2_ phase can coexist with the quasicrystalline phase(s). Therefore, it is pivotal to decipher the formation of the Al_11_Cr_2_ phase in order to enhance our understanding of the formation of quasicrystals as well as precipitations in the Al–Cr binary system. For the present investigation, we used high-pressure sinter­ing (HPS) of a stoichiometric Al:Cr mixture (molar ratio = 11:2) for crystal growth.

We have named the present phase η′-Al_11_Cr_2_. Its crystal structure is closely related to the η-Al_11_Cr_2_ phase previously reported by Cao & Kuo (2008*a* ▸), however with a different refined composition (Al:Cr ratio = 5.04). There are 66 Al and 14 Cr independent atomic positions and a total of 616 atoms (514 Al + 102 Cr) in the unit cell of η′-Al_11_Cr_2_. The crystal structure of η-Al_11_Cr_2_ comprises the same total number of atoms but with 516 Al atoms and 100 Cr atoms. In the η′-Al_11_Cr_2_ phase, there are only five mixed-occupied sites by Al and Cr atoms (all showing full occupancy), *viz*. Al4/Cr15, Al5/Cr16, Al11/Cr17, Al13/Cr18 and Al15/Cr19 with refined site occupation factors (s.o.f.) of 0.899 (5), 0.984 (4), 0.977 (5), 0.946 (4) and 0.945 (4) for Al4, Al5, Al11, Al13 and Al15, respectively. Quite differently, in the η-Al_11_Cr_2_ model there are six Al/Cr mixed-occupied sites (with an s.o.f. of 0.8291 for Al in three sites and 0.8291 for Cr in the remaining three sites) and 15 disordered positions for Al atoms, all split into two sites with a ratio of 0.8291:0.1709. A detailed comparison of coord­inates and occupancies for related atoms in the crystal structure models of η′-Al_11_Cr_2_ and η-Al_11_Cr_2_ can be found in the supporting information (Table S1).

**Figure 1:**
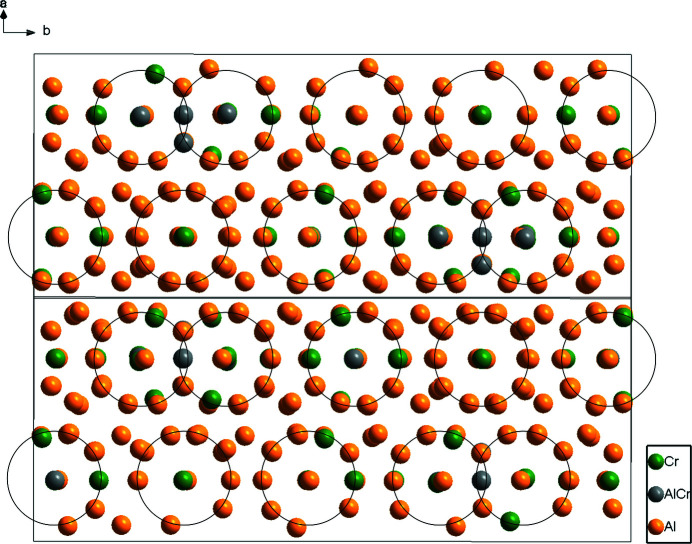
Icosa­hedrally surrounded sites (indicated by circles) in the crystal structure of η′-Al_11_Cr_2_ in a projection along [ 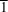 01].

**Figure 3:**
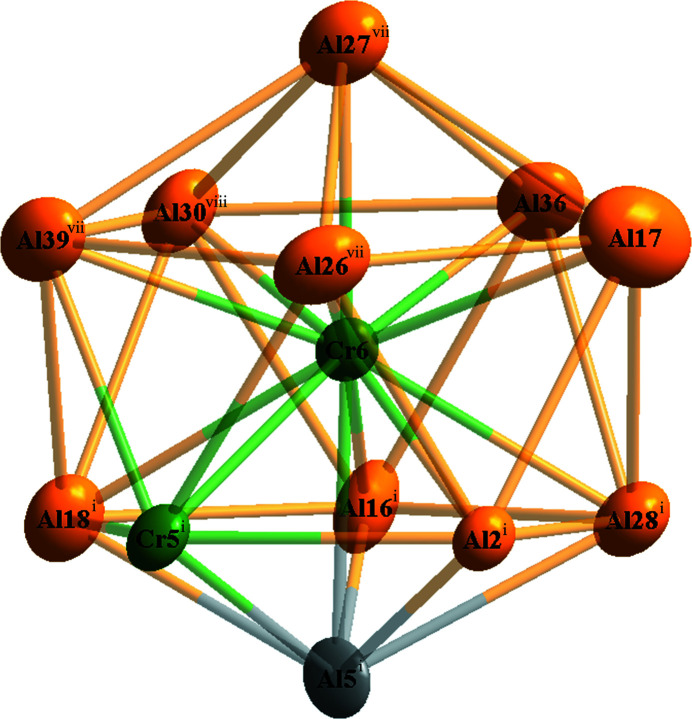
The environment of the Cr6 atom. Displacement ellipsoids are drawn at the 99.9% probability level. [Symmetry codes: (i) −*x* + 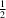 , −*y* + 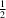 , −*z* + 1; (vii) −*x*, −*y* + 1, −*z* + 1; (viii) −*x* + 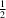 , *y* + 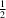 , −*z* + 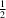 .]

Since the crystal structure of η-Al_11_Cr_2_ was described in detail (Cao & Kuo, 2008*a* ▸), we report here only the most important features. Figs. 1 ▸ and 2 ▸ illustrates the crystal structure of η′-Al_11_Cr_2_ in a projection along [ 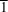 01] and [010], respectively. In Fig. 1 ▸, icosa­hedrally surrounded sites (indicated by circles) can be seen. One of such icosa­hedra (here around Cr6) was selected to show its chemical environment (Fig. 3 ▸). It is quite inter­esting that there are no split sites for Al atoms in the present η′-Al_11_Cr_2_ structure model. Scanning electron microscope (SEM) micrographs and energy dispersive X-ray spectroscopy (EDS) analysis of a fragment from which single crystals were selected for X-ray diffraction studies revealed that the η- and η′-Al_11_Cr_2_ phases have a very similar (and based on this method indistinguishable) chemical composition (see Fig. S1 and Table S2 in the supporting information). However, the refined chemical composition of the present η′-Al_11_Cr_2_ phase using single-crystal X-ray analysis reveals that it has two Al atoms fewer and two Cr atoms more than the reported η-Al_11_Cr_2_ phase.

## Synthesis and crystallization

The high-purity elements Al (indicated purity 99.8%; 1.2537 g) and Cr (indicated purity 99.95%; 0.4389 g) were mixed in the stoichiometric ratio 11:2 and ground in an agate mortar. The blended powders were placed into a cemented carbide grinding mound of 9.6 mm diameter and pressed at 4 MPa for about 5 min. A uniformly cylindrical block with 9.6 mm in diameter and 10.0 mm in height was obtained that was subsequently loaded into a six-anvil high-temperature high-pressure apparatus as described elsewhere (Xia *et al.*, 2018 ▸). For the present high-pressure sinter­ing experiments, the sample was pressurized up to 5 GPa and heated up to 1222 K for 30 minutes, slowly cooled to 1092 K and held at this temperature for 2 h, and then cooled to room temperature by turning off the furnace power. Suitable pieces of single-crystal grains were selected from the products for X-ray diffraction experiments.

## Refinement

Crystal data, data collection and structure refinement details are summarized in Table 1 ▸. Atom labelling and starting coordinates were taken from the η-Al_11_Cr_2_ model (Cao & Kuo, 2008*a* ▸). Those five sites (assuming full occupancy) with mixed occupation by Al and Cr atoms are Al4/Cr15, Al5/Cr16, Al11/Cr17, Al13/Cr18 and Al15/Cr19, with s.o.f.s 0.899 (5), 0.984 (4), 0.977 (5), 0.946 (4) and 0.945 (4) for Al4, Al5, Al11, Al13 and Al15. The remaining maximum and minimum electron densities are located 2.00 Å from atom Al43 and 1.08 Å from atom Al6, respectively.

## Supplementary Material

Crystal structure: contains datablock(s) I. DOI: 10.1107/S2414314620014121/wm4139sup1.cif

Structure factors: contains datablock(s) I. DOI: 10.1107/S2414314620014121/wm4139Isup2.hkl

Supplementary material (comparison of coordinates and occupancies; EDS analyses). DOI: 10.1107/S2414314620014121/wm4139sup3.pdf

CCDC reference: 2040163

Additional supporting information:  crystallographic information; 3D view; checkCIF report

## Figures and Tables

**Figure 2:**
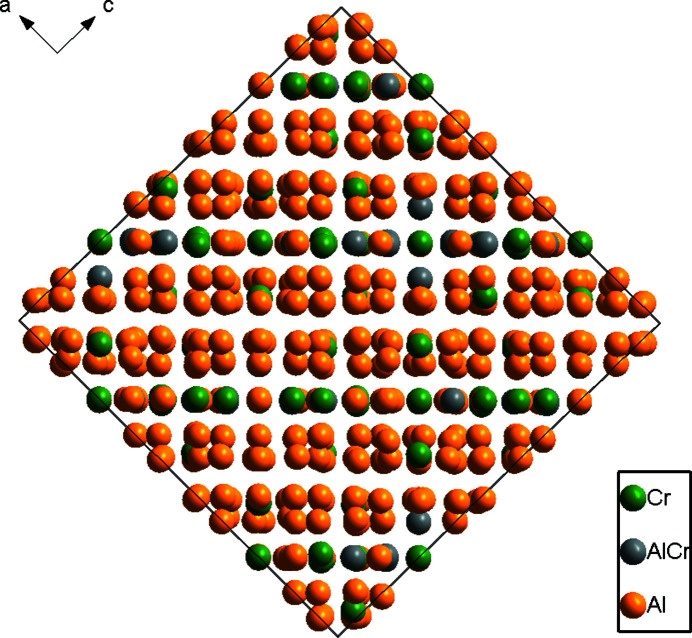
The crystal structure of η′-Al_11_Cr_2_ projected along [010].

**Table 1:** Experimental details

Crystal data	
Chemical formula	Al_2.95_Cr_0.59_
*M*_r_	110.18
Crystal system, space group	Monoclinic, *C*2/*c*
Temperature (K)	296
*a*, *b*, *c* (Å)	17.7519 (11), 30.4850 (11), 17.7526 (8)
β (°)	91.061 (1)
*V* (Å^3^)	9605.5 (8)
*Z*	174
Radiation type	Mo *K*α
μ (mm^−1^)	3.97
Crystal size (mm)	0.10 × 0.08 × 0.04

Data collection	
Diffractometer	Bruker D8 Venture Photon 100 CMOS
Absorption correction	Multi-scan (*SADABS*; Bruker, 2015 ▸)
*T*_min_, *T*_max_	0.633, 0.745
No. of measured, independent and observed [*I* > 2σ(*I*)] reflections	17649, 8944, 6039
*R*_int_	0.035
(sin θ/λ)_max_ (Å^−1^)	0.606

Refinement	
*R*[*F* ^2^ > 2σ(*F* ^2^)], *wR*(*F* ^2^), *S*	0.044, 0.081, 1.03
No. of reflections	8944
No. of parameters	704
Δρ_max_, Δρ_min_ (e Å^−3^)	0.95, −0.57

Computer programs: *APEX3* and *SAINT* (Bruker, 2015 ▸), *SHELXT2014/5* (Sheldrick, 2015*a* ▸), *SHELXL2017/1* (Sheldrick, 2015*b* ▸), *DIAMOND* (Brandenburg & Putz, 2017 ▸) and *publCIF* (Westrip, 2010 ▸).

## full crystallographic data

### Crystal data

**Table:**

Al_2.95_Cr_0.59_	*F*(000) = 9130
*M**_r_* = 110.18	*D*_x_ = 3.314 Mg m^−^^3^
Monoclinic, *C*2/*c*	Mo *K*α radiation, λ = 0.71073 Å
*a* = 17.7519 (11) Å	Cell parameters from 9683 reflections
*b* = 30.4850 (11) Å	θ = 2.3–25.5°
*c* = 17.7526 (8) Å	µ = 3.97 mm^−^^1^
β = 91.061 (1)°	*T* = 296 K
*V* = 9605.5 (8) Å^3^	Graininess, light
*Z* = 174	0.10 × 0.08 × 0.04 mm

### Data collection

**Table:**

Bruker D8 Venture Photon 100 CMOS diffractometer	6039 reflections with *I* > 2σ(*I*)
phi and ω scans	*R*_int_ = 0.035
Absorption correction: multi-scan (*SADABS*; Bruker, 2015)	θ_max_ = 25.5°, θ_min_ = 2.3°
*T*_min_ = 0.633, *T*_max_ = 0.745	*h* = −21→21
17649 measured reflections	*k* = −36→36
8944 independent reflections	*l* = −21→0

### Refinement

**Table:**

Refinement on *F*^2^	0 restraints
Least-squares matrix: full	*w* = 1/[σ^2^(*F*_o_^2^) + (0.0253*P*)^2^] where *P* = (*F*_o_^2^ + 2*F*_c_^2^)/3
*R*[*F*^2^ > 2σ(*F*^2^)] = 0.044	(Δ/σ)_max_ = 0.001
*wR*(*F*^2^) = 0.081	Δρ_max_ = 0.95 e Å^−^^3^
*S* = 1.03	Δρ_min_ = −0.57 e Å^−^^3^
8944 reflections	Extinction correction: SHELXL-2017/1 (Sheldrick 2017), Fc^*^=kFc[1+0.001xFc^2^λ^3^/sin(2θ)]^-1/4^
704 parameters	Extinction coefficient: 0.000029 (2)

### Special details

**Table:**

Geometry. All esds (except the esd in the dihedral angle between two l.s. planes) are estimated using the full covariance matrix. The cell esds are taken into account individually in the estimation of esds in distances, angles and torsion angles; correlations between esds in cell parameters are only used when they are defined by crystal symmetry. An approximate (isotropic) treatment of cell esds is used for estimating esds involving l.s. planes.

### Fractional atomic coordinates and isotropic or equivalent isotropic displacement parameters (Å^2^)

**Table:**

	*x*	*y*	*z*	*U*_iso_*/*U*_eq_	Occ. (<1)
Cr1	0.250000	0.250000	0.500000	0.0041 (3)	
Cr2	0.44734 (4)	0.24848 (2)	0.30280 (4)	0.00366 (19)	
Cr3	0.000000	0.39588 (3)	0.250000	0.0038 (3)	
Cr4	0.000000	0.10561 (3)	0.250000	0.0035 (3)	
Cr5	0.24978 (4)	−0.03633 (2)	0.49964 (4)	0.00377 (19)	
Cr6	0.16243 (4)	0.48613 (2)	0.41394 (4)	0.00351 (18)	
Cr7	−0.16469 (4)	−0.01328 (2)	0.58691 (4)	0.00311 (18)	
Cr8	0.40155 (4)	0.04038 (3)	0.34708 (4)	0.00335 (19)	
Cr9	0.09583 (4)	0.03550 (2)	0.65295 (4)	0.00351 (19)	
Cr10	0.15775 (4)	0.17684 (2)	0.10249 (4)	0.00398 (18)	
Cr11	0.14733 (4)	0.32385 (2)	0.09261 (4)	0.00369 (18)	
Cr12	0.18525 (4)	0.29801 (2)	0.23235 (4)	0.00487 (19)	
Cr13	0.14806 (4)	0.10915 (2)	0.60130 (4)	0.00353 (18)	
Cr14	−0.01777 (4)	0.20238 (2)	0.43544 (4)	0.00468 (19)	
Al1	0.000000	0.45786 (7)	0.750000	0.0077 (5)	
Al2	0.30099 (8)	0.03789 (5)	0.44894 (7)	0.0038 (3)	
Al3	−0.19430 (8)	0.10296 (5)	0.44181 (8)	0.0064 (3)	
Al4	0.05724 (8)	0.25039 (4)	0.30708 (8)	0.0092 (5)	0.899 (5)
Cr15	0.05724 (8)	0.25039 (4)	0.30708 (8)	0.0092 (5)	0.101 (5)
Al5	0.19798 (8)	0.03557 (5)	0.55224 (7)	0.0043 (5)	0.984 (4)
Cr16	0.19798 (8)	0.03557 (5)	0.55224 (7)	0.0043 (5)	0.016 (4)
Al6	−0.19137 (8)	0.39765 (5)	0.44365 (8)	0.0061 (3)	
Al7	0.08860 (8)	0.06822 (5)	0.34072 (8)	0.0068 (3)	
Al8	0.24834 (8)	0.05992 (5)	0.30739 (8)	0.0096 (4)	
Al9	0.15861 (8)	0.11356 (5)	0.21280 (8)	0.0098 (4)	
Al10	−0.25107 (8)	0.18478 (5)	0.49931 (8)	0.0069 (4)	
Al11	0.10237 (8)	0.25033 (5)	0.14778 (8)	0.0056 (5)	0.977 (5)
Cr17	0.10237 (8)	0.25033 (5)	0.14778 (8)	0.0056 (5)	0.023 (5)
Al12	0.000000	0.04101 (7)	0.750000	0.0053 (5)	
Al13	−0.04869 (7)	0.32273 (5)	0.30330 (7)	0.0061 (5)	0.946 (4)
Cr18	−0.04869 (7)	0.32273 (5)	0.30330 (7)	0.0061 (5)	0.054 (4)
Al14	0.05907 (8)	0.05710 (5)	0.50340 (8)	0.0066 (3)	
Al15	−0.05333 (7)	0.17819 (4)	0.29867 (7)	0.0056 (4)	0.945 (4)
Cr19	−0.05333 (7)	0.17819 (4)	0.29867 (7)	0.0056 (4)	0.055 (4)
Al16	0.24698 (8)	0.05713 (5)	0.69112 (8)	0.0067 (3)	
Al17	0.05822 (8)	0.44014 (5)	0.49793 (8)	0.0097 (4)	
Al18	0.21340 (8)	−0.02979 (5)	0.64991 (8)	0.0063 (3)	
Al19	0.09959 (8)	−0.03082 (5)	0.53662 (8)	0.0061 (3)	
Al20	−0.03199 (8)	0.01301 (5)	0.60072 (8)	0.0059 (3)	
Al21	0.13623 (8)	0.24868 (5)	0.00158 (8)	0.0073 (4)	
Al22	0.24848 (8)	0.25145 (5)	0.11385 (8)	0.0071 (4)	
Al23	0.14938 (8)	−0.01304 (5)	0.28092 (8)	0.0063 (3)	
Al24	0.28573 (8)	−0.02881 (5)	0.35037 (8)	0.0075 (4)	
Al25	0.28704 (8)	0.34248 (5)	0.15098 (8)	0.0079 (3)	
Al26	−0.10093 (8)	0.47223 (5)	0.46366 (8)	0.0068 (3)	
Al27	−0.02969 (8)	0.48609 (5)	0.59881 (8)	0.0071 (3)	
Al28	0.30215 (8)	0.10031 (5)	0.56536 (8)	0.0064 (3)	
Al29	0.18487 (8)	0.10057 (5)	0.44794 (8)	0.0062 (3)	
Al30	0.34886 (8)	0.01406 (5)	0.21925 (8)	0.0063 (3)	
Al31	0.09927 (8)	0.34223 (5)	0.33571 (8)	0.0073 (3)	
Al32	−0.15423 (8)	0.07608 (5)	0.59617 (8)	0.0067 (3)	
Al33	0.08565 (8)	0.15864 (5)	0.34924 (8)	0.0067 (3)	
Al34	−0.06083 (8)	0.04125 (5)	0.31042 (8)	0.0063 (3)	
Al35	−0.06187 (8)	0.46077 (5)	0.31249 (8)	0.0060 (3)	
Al36	0.08975 (8)	0.43312 (5)	0.33737 (8)	0.0068 (3)	
Al37	0.04419 (8)	0.11508 (5)	0.09782 (8)	0.0073 (3)	
Al38	−0.00194 (8)	0.10311 (5)	0.63352 (8)	0.0068 (3)	
Al39	−0.15352 (8)	0.42581 (5)	0.59628 (8)	0.0066 (3)	
Al40	0.11673 (8)	0.10287 (5)	0.75126 (8)	0.0071 (3)	
Al41	0.09933 (8)	0.15775 (5)	−0.03738 (8)	0.0075 (3)	
Al42	0.50498 (8)	0.10226 (5)	0.36801 (8)	0.0085 (4)	
Al43	0.38188 (8)	0.10254 (5)	0.24457 (8)	0.0076 (3)	
Al44	−0.03745 (8)	0.38746 (5)	0.40781 (8)	0.0105 (4)	
Al45	0.15248 (8)	0.38578 (5)	0.20640 (8)	0.0073 (3)	
Al46	0.29115 (8)	0.15696 (5)	0.15089 (8)	0.0080 (3)	
Al47	0.09882 (8)	0.34335 (5)	−0.04088 (8)	0.0079 (3)	
Al48	0.20769 (8)	0.17339 (5)	0.54219 (8)	0.0072 (3)	
Al49	0.11258 (8)	0.22881 (5)	0.45288 (8)	0.0075 (3)	
Al50	0.13633 (9)	0.32293 (5)	0.48425 (8)	0.0144 (4)	
Al51	0.23406 (8)	0.17736 (5)	0.38604 (9)	0.0143 (4)	
Al52	0.20329 (8)	0.27139 (5)	0.36267 (8)	0.0079 (3)	
Al53	0.09196 (8)	0.17539 (5)	0.65788 (8)	0.0063 (3)	
Al54	0.31655 (8)	0.27079 (5)	0.25076 (8)	0.0061 (3)	
Al55	0.34771 (8)	0.18353 (5)	0.28639 (8)	0.0063 (3)	
Al56	0.03640 (8)	0.31665 (5)	0.59768 (8)	0.0060 (3)	
Al57	0.49945 (8)	0.27059 (5)	0.43355 (8)	0.0066 (3)	
Al58	−0.09387 (8)	0.27614 (5)	0.65609 (8)	0.0077 (3)	
Al59	−0.05813 (8)	0.18594 (5)	0.69193 (8)	0.0072 (3)	
Al60	0.000000	0.33001 (7)	0.750000	0.0072 (5)	
Al61	0.05876 (8)	0.14688 (5)	0.50765 (8)	0.0071 (3)	
Al62	0.25770 (8)	0.35334 (5)	0.30867 (8)	0.0063 (3)	
Al63	0.35041 (8)	0.11692 (5)	0.39887 (8)	0.0064 (3)	
Al64	0.19528 (8)	0.20754 (5)	0.24318 (8)	0.0062 (3)	
Al65	−0.00686 (8)	0.29306 (5)	0.44513 (8)	0.0070 (3)	
Al66	0.15067 (8)	0.25680 (5)	0.59927 (8)	0.0070 (3)	

### Atomic displacement parameters (Å^2^)

**Table:**

	*U*^11^	*U*^22^	*U*^33^	*U*^12^	*U*^13^	*U*^23^
Cr1	0.0040 (6)	0.0053 (6)	0.0031 (6)	−0.0014 (5)	0.0000 (5)	0.0013 (5)
Cr2	0.0049 (4)	0.0028 (4)	0.0033 (4)	−0.0003 (3)	−0.0005 (3)	−0.0002 (3)
Cr3	0.0055 (6)	0.0019 (6)	0.0040 (6)	0.000	−0.0011 (5)	0.000
Cr4	0.0053 (6)	0.0022 (6)	0.0029 (6)	0.000	−0.0005 (5)	0.000
Cr5	0.0050 (4)	0.0028 (5)	0.0035 (4)	0.0009 (3)	0.0001 (3)	−0.0005 (3)
Cr6	0.0046 (4)	0.0035 (4)	0.0024 (4)	−0.0001 (3)	−0.0009 (3)	−0.0005 (3)
Cr7	0.0038 (4)	0.0032 (4)	0.0023 (4)	−0.0005 (3)	−0.0012 (3)	−0.0008 (3)
Cr8	0.0040 (4)	0.0036 (4)	0.0024 (4)	0.0002 (3)	−0.0001 (3)	0.0000 (3)
Cr9	0.0046 (4)	0.0034 (5)	0.0025 (4)	0.0002 (3)	−0.0006 (3)	0.0000 (3)
Cr10	0.0053 (4)	0.0026 (4)	0.0040 (4)	−0.0004 (4)	0.0003 (3)	−0.0001 (4)
Cr11	0.0051 (4)	0.0029 (4)	0.0031 (4)	−0.0008 (4)	0.0006 (3)	−0.0004 (3)
Cr12	0.0051 (4)	0.0038 (4)	0.0056 (4)	0.0000 (3)	−0.0014 (3)	−0.0013 (3)
Cr13	0.0047 (4)	0.0024 (4)	0.0035 (4)	0.0004 (4)	−0.0010 (3)	−0.0006 (4)
Cr14	0.0071 (4)	0.0035 (4)	0.0033 (4)	0.0009 (4)	−0.0022 (3)	−0.0002 (3)
Al1	0.0056 (12)	0.0099 (12)	0.0077 (12)	0.000	0.0004 (9)	0.000
Al2	0.0046 (8)	0.0036 (8)	0.0031 (8)	0.0003 (6)	−0.0002 (6)	0.0002 (6)
Al3	0.0077 (8)	0.0047 (8)	0.0068 (8)	−0.0011 (7)	−0.0003 (6)	0.0018 (7)
Al4	0.0104 (9)	0.0085 (9)	0.0087 (8)	0.0006 (7)	−0.0019 (6)	−0.0014 (6)
Cr15	0.0104 (9)	0.0085 (9)	0.0087 (8)	0.0006 (7)	−0.0019 (6)	−0.0014 (6)
Al5	0.0058 (9)	0.0031 (9)	0.0041 (8)	−0.0004 (6)	0.0007 (6)	−0.0026 (6)
Cr16	0.0058 (9)	0.0031 (9)	0.0041 (8)	−0.0004 (6)	0.0007 (6)	−0.0026 (6)
Al6	0.0078 (8)	0.0036 (8)	0.0067 (8)	0.0005 (7)	−0.0002 (6)	−0.0021 (6)
Al7	0.0083 (8)	0.0057 (8)	0.0063 (8)	0.0007 (7)	−0.0030 (6)	0.0001 (7)
Al8	0.0084 (8)	0.0113 (9)	0.0088 (8)	−0.0010 (7)	−0.0011 (7)	0.0013 (7)
Al9	0.0120 (9)	0.0068 (8)	0.0107 (8)	0.0005 (7)	0.0007 (7)	0.0017 (7)
Al10	0.0092 (8)	0.0069 (9)	0.0048 (8)	−0.0002 (7)	0.0016 (6)	0.0000 (7)
Al11	0.0103 (9)	0.0017 (8)	0.0049 (8)	−0.0017 (6)	−0.0012 (6)	−0.0001 (6)
Cr17	0.0103 (9)	0.0017 (8)	0.0049 (8)	−0.0017 (6)	−0.0012 (6)	−0.0001 (6)
Al12	0.0059 (12)	0.0064 (12)	0.0037 (11)	0.000	0.0028 (9)	0.000
Al13	0.0055 (8)	0.0070 (9)	0.0058 (8)	−0.0023 (6)	0.0016 (6)	0.0022 (6)
Cr18	0.0055 (8)	0.0070 (9)	0.0058 (8)	−0.0023 (6)	0.0016 (6)	0.0022 (6)
Al14	0.0079 (8)	0.0072 (8)	0.0047 (8)	−0.0008 (7)	−0.0006 (6)	−0.0008 (7)
Al15	0.0072 (8)	0.0052 (9)	0.0044 (8)	0.0018 (6)	−0.0005 (6)	−0.0007 (6)
Cr19	0.0072 (8)	0.0052 (9)	0.0044 (8)	0.0018 (6)	−0.0005 (6)	−0.0007 (6)
Al16	0.0091 (8)	0.0040 (8)	0.0070 (8)	0.0004 (7)	0.0008 (7)	0.0013 (6)
Al17	0.0099 (9)	0.0137 (9)	0.0056 (8)	−0.0008 (7)	0.0002 (7)	0.0002 (7)
Al18	0.0084 (9)	0.0060 (8)	0.0044 (8)	0.0001 (7)	−0.0006 (6)	0.0004 (6)
Al19	0.0081 (9)	0.0061 (8)	0.0041 (8)	0.0010 (7)	0.0003 (6)	0.0018 (6)
Al20	0.0051 (8)	0.0054 (8)	0.0072 (8)	−0.0023 (7)	−0.0019 (6)	−0.0001 (7)
Al21	0.0065 (9)	0.0079 (9)	0.0075 (8)	−0.0015 (7)	−0.0012 (6)	−0.0010 (7)
Al22	0.0072 (8)	0.0072 (9)	0.0069 (8)	−0.0015 (7)	−0.0008 (6)	−0.0006 (7)
Al23	0.0081 (8)	0.0079 (8)	0.0029 (8)	0.0005 (7)	−0.0011 (6)	−0.0019 (7)
Al24	0.0096 (9)	0.0080 (8)	0.0048 (8)	−0.0001 (7)	0.0000 (7)	0.0009 (7)
Al25	0.0094 (8)	0.0080 (8)	0.0063 (8)	−0.0016 (7)	−0.0007 (6)	0.0000 (7)
Al26	0.0081 (9)	0.0079 (8)	0.0042 (8)	0.0017 (7)	−0.0014 (6)	−0.0001 (7)
Al27	0.0087 (8)	0.0061 (8)	0.0066 (8)	0.0008 (7)	−0.0019 (6)	−0.0011 (7)
Al28	0.0069 (8)	0.0047 (8)	0.0074 (8)	0.0008 (7)	−0.0015 (6)	0.0005 (7)
Al29	0.0081 (8)	0.0049 (8)	0.0055 (8)	0.0012 (7)	−0.0009 (6)	−0.0010 (6)
Al30	0.0086 (8)	0.0052 (8)	0.0049 (8)	−0.0006 (7)	−0.0013 (6)	−0.0011 (7)
Al31	0.0067 (8)	0.0084 (9)	0.0066 (8)	0.0007 (7)	−0.0011 (6)	−0.0013 (7)
Al32	0.0078 (8)	0.0065 (8)	0.0059 (8)	−0.0012 (7)	−0.0021 (6)	0.0005 (7)
Al33	0.0069 (8)	0.0070 (8)	0.0062 (8)	0.0007 (7)	−0.0007 (6)	−0.0012 (7)
Al34	0.0089 (8)	0.0046 (8)	0.0052 (8)	−0.0008 (7)	−0.0006 (6)	0.0011 (7)
Al35	0.0066 (8)	0.0040 (8)	0.0075 (8)	0.0009 (7)	0.0001 (6)	−0.0008 (7)
Al36	0.0077 (8)	0.0070 (8)	0.0055 (8)	−0.0005 (7)	−0.0028 (6)	−0.0004 (7)
Al37	0.0089 (8)	0.0067 (8)	0.0062 (8)	−0.0004 (7)	−0.0008 (6)	0.0006 (7)
Al38	0.0079 (8)	0.0044 (8)	0.0081 (8)	0.0008 (7)	0.0005 (6)	0.0015 (7)
Al39	0.0082 (8)	0.0054 (8)	0.0061 (8)	0.0003 (7)	−0.0017 (6)	−0.0015 (7)
Al40	0.0091 (8)	0.0064 (8)	0.0057 (8)	0.0001 (7)	−0.0018 (6)	0.0002 (7)
Al41	0.0076 (8)	0.0074 (8)	0.0074 (8)	0.0008 (7)	−0.0007 (6)	−0.0020 (7)
Al42	0.0094 (8)	0.0042 (8)	0.0121 (8)	0.0013 (7)	0.0003 (7)	−0.0024 (7)
Al43	0.0109 (9)	0.0055 (8)	0.0063 (8)	0.0012 (7)	0.0004 (6)	0.0008 (7)
Al44	0.0118 (9)	0.0083 (9)	0.0114 (9)	−0.0024 (7)	0.0010 (7)	−0.0008 (7)
Al45	0.0080 (8)	0.0043 (8)	0.0096 (8)	0.0007 (7)	0.0007 (6)	0.0012 (7)
Al46	0.0068 (8)	0.0102 (9)	0.0069 (8)	0.0008 (7)	−0.0008 (6)	−0.0011 (7)
Al47	0.0078 (8)	0.0093 (9)	0.0066 (8)	0.0000 (7)	−0.0009 (6)	0.0015 (7)
Al48	0.0083 (8)	0.0050 (8)	0.0082 (8)	−0.0005 (7)	−0.0003 (6)	0.0015 (7)
Al49	0.0058 (8)	0.0093 (9)	0.0073 (8)	−0.0020 (7)	−0.0006 (6)	−0.0002 (7)
Al50	0.0182 (9)	0.0149 (10)	0.0100 (8)	−0.0062 (8)	0.0015 (7)	−0.0006 (7)
Al51	0.0108 (9)	0.0166 (10)	0.0156 (9)	0.0007 (8)	0.0029 (7)	0.0071 (8)
Al52	0.0089 (8)	0.0103 (9)	0.0045 (8)	−0.0019 (7)	0.0002 (6)	0.0018 (7)
Al53	0.0076 (8)	0.0045 (8)	0.0067 (8)	0.0002 (7)	0.0003 (6)	−0.0007 (7)
Al54	0.0045 (8)	0.0074 (8)	0.0063 (8)	0.0001 (7)	−0.0014 (6)	−0.0008 (7)
Al55	0.0073 (8)	0.0047 (8)	0.0068 (8)	0.0002 (7)	−0.0011 (6)	−0.0002 (7)
Al56	0.0074 (8)	0.0050 (8)	0.0054 (8)	0.0016 (7)	0.0003 (6)	−0.0005 (6)
Al57	0.0069 (8)	0.0074 (8)	0.0053 (8)	−0.0006 (7)	−0.0007 (6)	−0.0018 (7)
Al58	0.0081 (8)	0.0076 (8)	0.0075 (8)	0.0017 (7)	0.0011 (6)	0.0017 (7)
Al59	0.0084 (8)	0.0074 (8)	0.0059 (8)	−0.0004 (7)	0.0005 (6)	0.0004 (7)
Al60	0.0076 (11)	0.0074 (12)	0.0064 (11)	0.000	0.0000 (9)	0.000
Al61	0.0091 (8)	0.0060 (8)	0.0061 (8)	0.0001 (7)	−0.0031 (6)	0.0006 (7)
Al62	0.0072 (8)	0.0043 (8)	0.0074 (8)	−0.0005 (7)	−0.0025 (6)	−0.0013 (6)
Al63	0.0082 (8)	0.0037 (8)	0.0074 (8)	0.0009 (7)	0.0006 (6)	0.0013 (7)
Al64	0.0081 (8)	0.0063 (8)	0.0043 (8)	−0.0005 (7)	−0.0020 (6)	0.0010 (7)
Al65	0.0083 (8)	0.0051 (8)	0.0075 (8)	0.0006 (7)	−0.0034 (6)	0.0007 (7)
Al66	0.0058 (8)	0.0102 (8)	0.0051 (8)	0.0010 (7)	0.0001 (6)	−0.0013 (7)

### Geometric parameters (Å, º)

**Table:**

Cr1—Al66	2.5245 (14)	Al9—Al46	2.931 (2)
Cr1—Al66^i^	2.5246 (14)	Al9—Al18^xv^	2.959 (2)
Cr1—Al48^i^	2.5692 (14)	Al9—Al64	2.985 (2)
Cr1—Al48	2.5692 (14)	Al10—Al22^x^	2.813 (2)
Cr1—Al52^i^	2.6422 (14)	Al10—Al21^iv^	2.820 (2)
Cr1—Al52	2.6422 (14)	Al10—Al21^x^	2.850 (2)
Cr1—Al49	2.6443 (14)	Al10—Al22^iv^	2.858 (2)
Cr1—Al49^i^	2.6444 (14)	Al10—Al46^iv^	2.875 (2)
Cr2—Al66^i^	2.4874 (16)	Al10—Al47^x^	2.877 (2)
Cr2—Al53^i^	2.5254 (16)	Al10—Al41^iv^	2.885 (2)
Cr2—Al57	2.5726 (16)	Al10—Al25^x^	2.886 (2)
Cr2—Al54	2.5743 (16)	Al11—Al15^iv^	2.5550 (19)
Cr2—Al55	2.6673 (16)	Al11—Al13^iv^	2.562 (2)
Cr2—Al56^i^	2.6700 (16)	Al11—Al22	2.674 (2)
Cr2—Cr2^ii^	2.6719 (14)	Al11—Al21	2.675 (2)
Cr2—Al60^i^	2.740 (2)	Al11—Al64	2.681 (2)
Cr2—Al58^i^	2.7914 (16)	Al11—Al65^iv^	2.681 (2)
Cr2—Al58^iii^	2.7949 (16)	Cr17—Al22	2.674 (2)
Cr2—Al59^i^	2.8046 (16)	Cr17—Al21	2.675 (2)
Cr2—Al59^iii^	2.8058 (16)	Cr17—Al64	2.681 (2)
Cr3—Al36^iv^	2.4783 (15)	Cr17—Al65^iv^	2.681 (2)
Cr3—Al36	2.4783 (15)	Al12—Al40^xi^	2.802 (2)
Cr3—Al35^iv^	2.5289 (16)	Al12—Al40	2.802 (2)
Cr3—Al35	2.5289 (16)	Al12—Al38	2.803 (2)
Cr3—Al13^iv^	2.5778 (16)	Al12—Al38^xi^	2.803 (2)
Cr3—Al13	2.5779 (16)	Al12—Al23^vi^	2.8295 (15)
Cr3—Cr18	2.5779 (16)	Al12—Al23^ix^	2.8295 (15)
Cr3—Al31^iv^	2.8277 (15)	Al12—Al20^xi^	2.8317 (15)
Cr3—Al31	2.8277 (15)	Al12—Al20	2.8317 (15)
Cr3—Al45^iv^	2.8457 (14)	Al12—Al34^ix^	2.941 (2)
Cr3—Al45	2.8458 (14)	Al12—Al34^vi^	2.941 (2)
Cr3—Al44	2.9026 (15)	Al13—Al13^iv^	2.587 (3)
Cr4—Al34	2.4919 (16)	Al13—Al45^iv^	2.666 (2)
Cr4—Al34^iv^	2.4919 (16)	Al13—Al31^iv^	2.6772 (19)
Cr4—Al7^iv^	2.5045 (15)	Al13—Al44	2.713 (2)
Cr4—Al7	2.5045 (15)	Al13—Al31	2.7436 (19)
Cr4—Al15^iv^	2.5630 (16)	Al13—Al65	2.7639 (19)
Cr4—Al15	2.5630 (16)	Cr18—Al45^iv^	2.666 (2)
Cr4—Cr19	2.5630 (16)	Cr18—Al31^iv^	2.6772 (19)
Cr4—Al33^iv^	2.8159 (15)	Cr18—Al44	2.713 (2)
Cr4—Al33	2.8160 (16)	Cr18—Al31	2.7436 (19)
Cr4—Al37^iv^	2.8417 (14)	Cr18—Al65	2.7639 (19)
Cr4—Al37	2.8418 (14)	Al14—Al61	2.738 (2)
Cr4—Al9	2.9141 (15)	Al14—Al20	2.740 (2)
Cr5—Al6^v^	2.4835 (16)	Al14—Al29	2.792 (2)
Cr5—Al3^vi^	2.4927 (16)	Al14—Al19	2.834 (2)
Cr5—Al5	2.5599 (16)	Al14—Al20^vi^	2.861 (2)
Cr5—Cr16	2.5599 (16)	Al14—Al38	2.927 (2)
Cr5—Al2	2.6051 (16)	Al15—Al15^iv^	2.587 (3)
Cr5—Cr7^vi^	2.6161 (10)	Al15—Al37^iv^	2.663 (2)
Cr5—Cr6^i^	2.6525 (10)	Al15—Al33	2.6770 (19)
Cr5—Al39^v^	2.6670 (15)	Al15—Al33^iv^	2.7429 (19)
Cr5—Al32^vi^	2.6704 (15)	Al15—Al64^iv^	2.7626 (19)
Cr5—Al24	2.7469 (16)	Cr19—Al37^iv^	2.663 (2)
Cr5—Al26^v^	2.7500 (16)	Cr19—Al33	2.6770 (19)
Cr5—Al19	2.7626 (16)	Cr19—Al33^iv^	2.7429 (19)
Cr6—Al36	2.4614 (16)	Cr19—Al64^iv^	2.7626 (19)
Cr6—Al27^vii^	2.5103 (16)	Al16—Al23^ix^	2.730 (2)
Cr6—Al30^viii^	2.5175 (15)	Al16—Al62^i^	2.731 (2)
Cr6—Al2^i^	2.6122 (15)	Al16—Al28	2.785 (2)
Cr6—Al5^i^	2.6235 (15)	Al16—Al18	2.810 (2)
Cr6—Al39^vii^	2.6950 (16)	Al16—Al30^ix^	2.863 (2)
Cr6—Al28^i^	2.7324 (16)	Al16—Al40	2.919 (2)
Cr6—Al26^vii^	2.7591 (16)	Al16—Al36^i^	2.966 (2)
Cr6—Al17	2.7773 (16)	Al17—Al27	2.776 (2)
Cr6—Al16^i^	2.8134 (16)	Al17—Al44	2.815 (2)
Cr6—Al18^i^	2.8298 (16)	Al17—Al26^vii^	2.856 (2)
Cr7—Al23^vi^	2.4899 (15)	Al17—Al27^vii^	2.869 (2)
Cr7—Al7^vi^	2.4933 (16)	Al17—Al36	2.923 (2)
Cr7—Al20	2.4959 (16)	Al17—Al42^i^	2.948 (2)
Cr7—Al2^vi^	2.6012 (15)	Al17—Al63^i^	2.984 (2)
Cr7—Al5^vi^	2.6191 (15)	Al18—Al30^ix^	2.724 (2)
Cr7—Al32	2.7351 (16)	Al18—Al19	2.824 (2)
Cr7—Al29^vi^	2.7541 (16)	Al18—Al34^vi^	2.833 (2)
Cr7—Al24^vi^	2.7554 (16)	Al18—Al39^v^	2.899 (2)
Cr7—Al8^vi^	2.8019 (16)	Al18—Al23^ix^	2.916 (2)
Cr7—Al14^vi^	2.8253 (16)	Al19—Al20^vi^	2.7516 (19)
Cr7—Al19^vi^	2.8365 (16)	Al19—Al34^vi^	2.832 (2)
Cr8—Al1^i^	2.4777 (7)	Al19—Al32^vi^	2.913 (2)
Cr8—Al2	2.5654 (15)	Al19—Al20	2.938 (2)
Cr8—Al30	2.5671 (15)	Al20—Al34^vi^	2.800 (2)
Cr8—Al27^i^	2.5820 (16)	Al20—Al38	2.856 (2)
Cr8—Al35^v^	2.5890 (16)	Al20—Al32	2.899 (2)
Cr8—Al43	2.6460 (16)	Al20—Al23^vi^	2.987 (2)
Cr8—Al42	2.6539 (16)	Al21—Al57^xvi^	2.755 (2)
Cr8—Al63	2.6730 (16)	Al21—Al22	2.793 (2)
Cr8—Al8	2.8597 (16)	Al21—Al65^iv^	2.842 (2)
Cr8—Al17^i^	2.8915 (16)	Al21—Al22^xvii^	2.923 (2)
Cr8—Al26^v^	2.9333 (16)	Al21—Al41	2.928 (2)
Cr8—Al24	2.9467 (16)	Al22—Al54	2.758 (2)
Cr9—Al12	2.4493 (7)	Al22—Al64	2.835 (2)
Cr9—Al34^vi^	2.5095 (16)	Al22—Al25	2.930 (2)
Cr9—Al20	2.5300 (15)	Al23—Al24	2.739 (2)
Cr9—Al23^ix^	2.5403 (15)	Al23—Al34^iv^	2.783 (2)
Cr9—Al5	2.5701 (15)	Al23—Al40^xv^	2.846 (2)
Cr9—Cr16	2.5701 (15)	Al23—Al32^vi^	2.908 (2)
Cr9—Cr13	2.6024 (10)	Al24—Al35^v^	2.818 (2)
Cr9—Al38	2.7125 (16)	Al24—Al26^v^	2.819 (2)
Cr9—Al40	2.7163 (16)	Al24—Al30	2.912 (2)
Cr9—Al14	2.8006 (15)	Al24—Al32^vi^	2.917 (2)
Cr9—Al16	2.8329 (16)	Al25—Al54	2.856 (2)
Cr9—Al18	2.8853 (16)	Al25—Al32^iii^	2.870 (2)
Cr10—Al3^iv^	2.4760 (16)	Al25—Al62	2.876 (2)
Cr10—Al10^iv^	2.4859 (16)	Al25—Al41^xvii^	2.878 (2)
Cr10—Al15^iv^	2.5764 (16)	Al25—Al45	2.916 (2)
Cr10—Al46	2.5764 (16)	Al25—Al40^i^	2.932 (2)
Cr10—Cr17	2.5809 (16)	Al25—Al59^iii^	2.960 (2)
Cr10—Al11	2.5809 (16)	Al26—Al27	2.7243 (19)
Cr10—Cr14^iv^	2.6781 (10)	Al26—Al35	2.806 (2)
Cr10—Al41	2.7358 (16)	Al26—Al27^vii^	2.884 (2)
Cr10—Al64	2.7379 (16)	Al26—Al39	2.915 (2)
Cr10—Al9	2.7487 (16)	Al26—Al44	2.995 (2)
Cr10—Al37	2.7585 (16)	Al27—Al35^vii^	2.766 (2)
Cr10—Al22	2.7922 (16)	Al27—Al42^i^	2.790 (2)
Cr11—Al6^iv^	2.4712 (16)	Al27—Al39	2.865 (2)
Cr11—Al10^iii^	2.4852 (16)	Al27—Al36^vii^	2.921 (2)
Cr11—Al13^iv^	2.5693 (15)	Al28—Al50^i^	2.735 (2)
Cr11—Al47	2.5759 (16)	Al28—Al36^i^	2.7532 (19)
Cr11—Cr17	2.5784 (16)	Al28—Al48	2.814 (2)
Cr11—Al11	2.5784 (16)	Al28—Al62^i^	2.866 (2)
Cr11—Cr12	2.6768 (10)	Al28—Al29	2.919 (2)
Cr11—Al25	2.7299 (16)	Al29—Al51	2.736 (2)
Cr11—Al65^iv^	2.7359 (16)	Al29—Al48	2.805 (2)
Cr11—Al44^iv^	2.7504 (16)	Al29—Al61	2.866 (2)
Cr11—Al45	2.7653 (16)	Al30—Al35^v^	2.792 (2)
Cr11—Al21	2.8088 (16)	Al30—Al43	2.795 (2)
Cr12—Al52	2.4670 (16)	Al30—Al39^iii^	2.850 (2)
Cr12—Al54	2.4899 (16)	Al30—Al36^xviii^	2.885 (2)
Cr12—Al62	2.5041 (16)	Al31—Al50	2.769 (2)
Cr12—Cr17	2.5398 (16)	Al31—Al36	2.776 (2)
Cr12—Al11	2.5398 (16)	Al31—Al45	2.829 (2)
Cr12—Al13^iv^	2.6054 (15)	Al31—Al52	2.876 (2)
Cr12—Al25	2.6994 (16)	Al31—Al62	2.882 (2)
Cr12—Al31	2.7599 (16)	Al32—Al41^iv^	2.877 (2)
Cr12—Al64	2.7704 (16)	Al32—Al38	2.892 (2)
Cr12—Al45	2.7749 (16)	Al32—Al40^xi^	2.895 (2)
Cr12—Al22	2.7908 (16)	Al33—Al51	2.762 (2)
Cr13—Al48	2.4695 (16)	Al33—Al37^iv^	2.835 (2)
Cr13—Al53	2.4730 (16)	Al33—Al49	2.856 (2)
Cr13—Al61	2.5501 (15)	Al33—Al61	2.883 (2)
Cr13—Al62^i^	2.5615 (15)	Al34—Al37^iv^	2.791 (2)
Cr13—Cr16	2.5698 (16)	Al35—Al45^iv^	2.812 (2)
Cr13—Al5	2.5698 (16)	Al35—Al36^iv^	2.826 (2)
Cr13—Al40	2.7364 (16)	Al35—Al44	2.832 (2)
Cr13—Al38	2.7401 (16)	Al35—Al36	2.847 (2)
Cr13—Al14	2.8164 (16)	Al36—Al44	2.950 (2)
Cr13—Al29	2.8238 (16)	Al36—Al45	2.971 (2)
Cr13—Al28	2.8331 (16)	Al37—Al61^iv^	2.767 (2)
Cr14—Al49	2.4645 (16)	Al37—Al41	2.915 (2)
Cr14—Al57^i^	2.4833 (16)	Al38—Al53	2.792 (2)
Cr14—Al61	2.5073 (16)	Al38—Al61	2.833 (2)
Cr14—Al11^iv^	2.5480 (16)	Al38—Al59	2.913 (2)
Cr14—Cr19	2.6039 (15)	Al38—Al40^xi^	2.9138 (19)
Cr14—Al15	2.6039 (15)	Al38—Al41^iv^	2.927 (2)
Cr14—Al41^iv^	2.7054 (16)	Al38—Al40	2.940 (2)
Cr14—Al33	2.7557 (16)	Al39—Al43^x^	2.830 (2)
Cr14—Al37^iv^	2.7641 (16)	Al39—Al42^i^	2.833 (2)
Cr14—Al65	2.7765 (16)	Al39—Al47^iv^	2.875 (2)
Cr14—Al21^iv^	2.7841 (16)	Al39—Al46^x^	2.882 (2)
Al1—Al42^i^	2.784 (2)	Al40—Al53	2.793 (2)
Al1—Al42^x^	2.784 (2)	Al40—Al62^i^	2.823 (2)
Al1—Al43^x^	2.791 (2)	Al40—Al59^xi^	2.924 (2)
Al1—Al43^i^	2.791 (2)	Al41—Al57^xvi^	2.855 (2)
Al1—Al27^xi^	2.8583 (15)	Al41—Al61^iv^	2.884 (2)
Al1—Al27	2.8583 (15)	Al41—Al59^iv^	2.954 (2)
Al1—Al30^i^	2.8592 (15)	Al42—Al56^i^	2.653 (2)
Al1—Al30^x^	2.8592 (15)	Al42—Al47^xix^	2.836 (2)
Al1—Al35^xii^	2.938 (2)	Al42—Al63	2.843 (2)
Al1—Al35^vii^	2.938 (2)	Al42—Al43^ii^	2.860 (2)
Al2—Al5	2.6145 (19)	Al42—Al60^i^	2.942 (2)
Al2—Cr16	2.6145 (19)	Al43—Al55	2.652 (2)
Al2—Al26^v^	2.663 (2)	Al43—Al46	2.831 (2)
Al2—Al24	2.693 (2)	Al43—Al63	2.840 (2)
Al2—Al63	2.719 (2)	Al43—Al60^i^	2.937 (2)
Al2—Al17^i^	2.7382 (19)	Al44—Al45^iv^	2.851 (2)
Al2—Al8	2.748 (2)	Al44—Al47^iv^	2.945 (2)
Al2—Al28	2.809 (2)	Al45—Al62	2.764 (2)
Al3—Al18^vi^	2.779 (2)	Al46—Al55	2.713 (2)
Al3—Al19^vi^	2.791 (2)	Al46—Al47^xvii^	2.788 (2)
Al3—Al37^iv^	2.793 (2)	Al46—Al64	2.839 (2)
Al3—Al9^iv^	2.847 (2)	Al46—Al58^iii^	2.886 (2)
Al3—Al46^iv^	2.876 (2)	Al47—Al56^iv^	2.711 (2)
Al3—Al10	2.884 (2)	Al47—Al65^iv^	2.830 (2)
Al3—Al41^iv^	2.899 (2)	Al47—Al58^iv^	2.895 (2)
Al3—Al6^xiii^	2.899 (2)	Al48—Al50^i^	2.819 (2)
Al3—Al39^xiii^	2.908 (2)	Al48—Al61	2.821 (2)
Al3—Al32	2.935 (2)	Al48—Al51	2.822 (2)
Al4—Al49	2.8291 (19)	Al48—Al62^i^	2.827 (2)
Al4—Al52	2.830 (2)	Al48—Al52^i^	2.844 (2)
Al4—Al4^iv^	2.843 (3)	Al48—Al49	2.849 (2)
Al4—Al15^iv^	2.8928 (19)	Al48—Al66	2.925 (2)
Al4—Al13	2.8981 (19)	Al48—Al53	2.9324 (19)
Al4—Al33	2.937 (2)	Al49—Al52	2.634 (2)
Al4—Al31	2.939 (2)	Al49—Al66	2.806 (2)
Al4—Al15	2.9509 (19)	Al49—Al61	2.851 (2)
Al4—Al13^iv^	2.9521 (19)	Al49—Al57^i^	2.858 (2)
Al4—Al11	2.9536 (19)	Al49—Al65	2.888 (2)
Al4—Al11^iv^	2.9588 (19)	Al49—Al51	2.935 (2)
Cr15—Al49	2.8291 (19)	Al50—Al56	2.715 (2)
Cr15—Al52	2.830 (2)	Al50—Al63^i^	2.776 (2)
Cr15—Cr18	2.8981 (19)	Al50—Al65	2.776 (2)
Cr15—Al33	2.937 (2)	Al50—Al66	2.877 (2)
Cr15—Al31	2.939 (2)	Al50—Al52	2.939 (2)
Cr15—Cr19	2.9509 (19)	Al51—Al55	2.714 (2)
Cr15—Cr17	2.9536 (19)	Al51—Al64	2.773 (2)
Al5—Al18	2.652 (2)	Al51—Al63	2.774 (2)
Al5—Al14	2.6804 (19)	Al51—Al66^i^	2.875 (2)
Al5—Al16	2.6809 (19)	Al51—Al52	2.946 (2)
Al5—Al19	2.684 (2)	Al52—Al66^i^	2.802 (2)
Al5—Al28	2.712 (2)	Al52—Al62	2.851 (2)
Al5—Al29	2.719 (2)	Al52—Al54	2.853 (2)
Cr16—Al18	2.652 (2)	Al52—Al64	2.880 (2)
Cr16—Al14	2.6804 (19)	Al53—Al59	2.762 (2)
Cr16—Al16	2.6809 (19)	Al53—Al59^xi^	2.763 (2)
Cr16—Al19	2.684 (2)	Al53—Al54^i^	2.804 (2)
Cr16—Al28	2.712 (2)	Al53—Al57^i^	2.808 (2)
Cr16—Al29	2.719 (2)	Al53—Al61	2.8561 (19)
Al6—Al45^iv^	2.788 (2)	Al53—Al62^i^	2.8608 (19)
Al6—Al26	2.802 (2)	Al53—Al66	2.893 (2)
Al6—Al24^xiv^	2.812 (2)	Al54—Al58^iii^	2.739 (2)
Al6—Al44	2.834 (2)	Al54—Al55	2.788 (2)
Al6—Al47^iv^	2.884 (2)	Al54—Al59^iii^	2.805 (2)
Al6—Al10^xiii^	2.902 (2)	Al54—Al66^i^	2.842 (2)
Al6—Al25^iv^	2.903 (2)	Al54—Al64	2.892 (2)
Al6—Al39	2.909 (2)	Al54—Al62	2.919 (2)
Al6—Al32^xiii^	2.929 (2)	Al55—Al66^i^	2.725 (2)
Al7—Al29	2.7196 (19)	Al55—Al60^i^	2.8214 (14)
Al7—Al33	2.761 (2)	Al55—Al58^iii^	2.833 (2)
Al7—Al34	2.819 (2)	Al55—Al63	2.848 (2)
Al7—Al34^iv^	2.841 (2)	Al55—Al56^i^	2.883 (2)
Al7—Al20^vi^	2.874 (2)	Al55—Al64	2.893 (2)
Al7—Al23	2.910 (2)	Al56—Al66	2.7282 (19)
Al7—Al8	2.919 (2)	Al56—Al57^i^	2.788 (2)
Al7—Al9	2.952 (2)	Al56—Al60	2.8213 (14)
Al7—Al14	2.964 (2)	Al56—Al58	2.835 (2)
Al7—Al37^iv^	2.980 (2)	Al56—Al63^i^	2.853 (2)
Al8—Al30	2.773 (2)	Al56—Al65	2.892 (2)
Al8—Al9	2.816 (2)	Al57—Al58^i^	2.733 (2)
Al8—Al23	2.868 (2)	Al57—Al59^i^	2.809 (2)
Al8—Al24	2.885 (2)	Al57—Al66^i^	2.843 (2)
Al8—Al43	2.941 (2)	Al57—Al65^i^	2.900 (2)
Al8—Al63	2.972 (2)	Al57—Al61^i^	2.920 (2)
Al8—Al18^xv^	2.997 (2)	Al58—Al60	2.8546 (19)
Al9—Al15^iv^	2.721 (2)	Al58—Al59	2.890 (2)
Al9—Al34^iv^	2.831 (2)	Al59—Al59^xi^	2.890 (3)
Al9—Al37	2.853 (2)		
			
Al66—Cr1—Al66^i^	180.00 (4)	Al23—Al24—Cr7^vi^	53.90 (4)
Al66—Cr1—Al48^i^	109.91 (5)	Cr5—Al24—Cr7^vi^	56.78 (4)
Al66^i^—Cr1—Al48^i^	70.09 (5)	Al2—Al24—Al6^v^	101.98 (6)
Al66—Cr1—Al48	70.09 (5)	Al23—Al24—Al6^v^	121.46 (7)
Al66^i^—Cr1—Al48	109.91 (5)	Cr5—Al24—Al6^v^	53.06 (4)
Al48^i^—Cr1—Al48	180.0	Cr7^vi^—Al24—Al6^v^	103.80 (6)
Al66—Cr1—Al52^i^	65.63 (5)	Al2—Al24—Al35^v^	98.90 (6)
Al66^i^—Cr1—Al52^i^	114.37 (5)	Al23—Al24—Al35^v^	139.19 (7)
Al48^i^—Cr1—Al52^i^	113.88 (4)	Cr5—Al24—Al35^v^	117.52 (6)
Al48—Cr1—Al52^i^	66.12 (4)	Cr7^vi^—Al24—Al35^v^	155.28 (7)
Al66—Cr1—Al52	114.37 (5)	Al6^v^—Al24—Al35^v^	85.51 (6)
Al66^i^—Cr1—Al52	65.63 (5)	Al2—Al24—Al26^v^	57.72 (5)
Al48^i^—Cr1—Al52	66.12 (4)	Al23—Al24—Al26^v^	158.84 (7)
Al48—Cr1—Al52	113.88 (4)	Cr5—Al24—Al26^v^	59.21 (4)
Al52^i^—Cr1—Al52	180.0	Cr7^vi^—Al24—Al26^v^	104.96 (6)
Al66—Cr1—Al49	65.71 (5)	Al6^v^—Al24—Al26^v^	59.69 (5)
Al66^i^—Cr1—Al49	114.29 (5)	Al35^v^—Al24—Al26^v^	59.72 (5)
Al48^i^—Cr1—Al49	113.75 (4)	Al2—Al24—Al8	58.92 (5)
Al48—Cr1—Al49	66.24 (4)	Al23—Al24—Al8	61.26 (5)
Al52^i^—Cr1—Al49	120.23 (4)	Cr5—Al24—Al8	106.08 (6)
Al52—Cr1—Al49	59.77 (4)	Cr7^vi^—Al24—Al8	59.53 (4)
Al66—Cr1—Al49^i^	114.29 (5)	Al6^v^—Al24—Al8	158.96 (7)
Al66^i^—Cr1—Al49^i^	65.71 (5)	Al35^v^—Al24—Al8	105.06 (6)
Al48^i^—Cr1—Al49^i^	66.25 (4)	Al26^v^—Al24—Al8	109.63 (7)
Al48—Cr1—Al49^i^	113.76 (4)	Al2—Al24—Al30	98.34 (6)
Al52^i^—Cr1—Al49^i^	59.77 (4)	Al23—Al24—Al30	84.91 (6)
Al52—Cr1—Al49^i^	120.23 (4)	Cr5—Al24—Al30	155.29 (7)
Al49—Cr1—Al49^i^	180.0	Cr7^vi^—Al24—Al30	115.38 (6)
Al66^i^—Cr2—Al53^i^	70.48 (5)	Al6^v^—Al24—Al30	140.80 (7)
Al66^i^—Cr2—Al57	68.33 (5)	Al35^v^—Al24—Al30	58.29 (5)
Al53^i^—Cr2—Al57	66.82 (5)	Al26^v^—Al24—Al30	106.57 (6)
Al66^i^—Cr2—Al54	68.29 (5)	Al8—Al24—Al30	57.17 (5)
Al53^i^—Cr2—Al54	66.70 (5)	Al2—Al24—Al32^vi^	103.48 (6)
Al57—Cr2—Al54	124.15 (5)	Al23—Al24—Al32^vi^	61.79 (5)
Al66^i^—Cr2—Al55	63.73 (5)	Cr5—Al24—Al32^vi^	56.16 (4)
Al53^i^—Cr2—Al55	121.76 (5)	Cr7^vi^—Al24—Al32^vi^	57.57 (4)
Al57—Cr2—Al55	121.30 (5)	Al6^v^—Al24—Al32^vi^	61.47 (5)
Al54—Cr2—Al55	64.23 (5)	Al35^v^—Al24—Al32^vi^	143.09 (7)
Al66^i^—Cr2—Al56^i^	63.76 (5)	Al26^v^—Al24—Al32^vi^	109.78 (6)
Al53^i^—Cr2—Al56^i^	121.84 (5)	Al8—Al24—Al32^vi^	111.57 (6)
Al57—Cr2—Al56^i^	64.22 (5)	Al30—Al24—Al32^vi^	143.51 (6)
Al54—Cr2—Al56^i^	121.31 (5)	Al2—Al24—Cr8	53.89 (4)
Al55—Cr2—Al56^i^	65.39 (5)	Al23—Al24—Cr8	118.46 (6)
Al66^i^—Cr2—Cr2^ii^	176.29 (4)	Cr5—Al24—Cr8	104.69 (5)
Al53^i^—Cr2—Cr2^ii^	113.23 (4)	Cr7^vi^—Al24—Cr8	102.96 (5)
Al57—Cr2—Cr2^ii^	112.86 (5)	Al6^v^—Al24—Cr8	119.27 (6)
Al54—Cr2—Cr2^ii^	112.73 (5)	Al35^v^—Al24—Cr8	53.32 (4)
Al55—Cr2—Cr2^ii^	113.24 (4)	Al26^v^—Al24—Cr8	61.12 (4)
Al56^i^—Cr2—Cr2^ii^	113.31 (4)	Al8—Al24—Cr8	58.72 (4)
Al66^i^—Cr2—Al60^i^	115.47 (5)	Al30—Al24—Cr8	51.97 (4)
Al53^i^—Cr2—Al60^i^	174.05 (5)	Al32^vi^—Al24—Cr8	157.35 (6)
Al57—Cr2—Al60^i^	114.71 (4)	Cr12—Al25—Cr11	59.08 (4)
Al54—Cr2—Al60^i^	114.78 (4)	Cr12—Al25—Al54	53.17 (4)
Al55—Cr2—Al60^i^	62.88 (4)	Cr11—Al25—Al54	103.36 (6)
Al56^i^—Cr2—Al60^i^	62.85 (4)	Cr12—Al25—Al32^iii^	150.10 (7)
Cr2^ii^—Cr2—Al60^i^	60.82 (3)	Cr11—Al25—Al32^iii^	112.62 (6)
Al66^i^—Cr2—Al58^i^	117.40 (5)	Al54—Al25—Al32^iii^	143.99 (7)
Al53^i^—Cr2—Al58^i^	115.83 (5)	Cr12—Al25—Al62	53.26 (4)
Al57—Cr2—Al58^i^	61.10 (5)	Cr11—Al25—Al62	102.34 (5)
Al54—Cr2—Al58^i^	174.12 (5)	Al54—Al25—Al62	61.23 (5)
Al55—Cr2—Al58^i^	116.21 (5)	Al32^iii^—Al25—Al62	107.69 (6)
Al56^i^—Cr2—Al58^i^	62.49 (5)	Cr12—Al25—Al41^xvii^	149.13 (7)
Cr2^ii^—Cr2—Al58^i^	61.49 (4)	Cr11—Al25—Al41^xvii^	112.15 (6)
Al60^i^—Cr2—Al58^i^	62.12 (4)	Al54—Al25—Al41^xvii^	108.05 (6)
Al66^i^—Cr2—Al58^iii^	117.36 (5)	Al32^iii^—Al25—Al41^xvii^	60.06 (5)
Al53^i^—Cr2—Al58^iii^	115.82 (5)	Al62—Al25—Al41^xvii^	145.50 (7)
Al57—Cr2—Al58^iii^	174.10 (5)	Cr12—Al25—Al10^iii^	101.94 (6)
Al54—Cr2—Al58^iii^	61.18 (5)	Cr11—Al25—Al10^iii^	52.44 (4)
Al55—Cr2—Al58^iii^	62.44 (5)	Al54—Al25—Al10^iii^	113.29 (6)
Al56^i^—Cr2—Al58^iii^	116.11 (5)	Al32^iii^—Al25—Al10^iii^	90.76 (6)
Cr2^ii^—Cr2—Al58^iii^	61.36 (4)	Al62—Al25—Al10^iii^	153.84 (7)
Al60^i^—Cr2—Al58^iii^	62.08 (4)	Al41^xvii^—Al25—Al10^iii^	60.06 (5)
Al58^i^—Cr2—Al58^iii^	113.45 (5)	Cr12—Al25—Al6^iv^	102.14 (6)
Al66^i^—Cr2—Al59^i^	121.46 (5)	Cr11—Al25—Al6^iv^	51.93 (4)
Al53^i^—Cr2—Al59^i^	62.16 (5)	Al54—Al25—Al6^iv^	154.28 (7)
Al57—Cr2—Al59^i^	62.80 (5)	Al32^iii^—Al25—Al6^iv^	60.98 (5)
Al54—Cr2—Al59^i^	116.80 (5)	Al62—Al25—Al6^iv^	112.45 (6)
Al55—Cr2—Al59^i^	174.81 (5)	Al41^xvii^—Al25—Al6^iv^	90.35 (6)
Al56^i^—Cr2—Al59^i^	116.15 (5)	Al10^iii^—Al25—Al6^iv^	60.17 (5)
Cr2^ii^—Cr2—Al59^i^	61.58 (4)	Cr12—Al25—Al45	59.08 (4)
Al60^i^—Cr2—Al59^i^	112.97 (4)	Cr11—Al25—Al45	58.54 (4)
Al58^i^—Cr2—Al59^i^	62.19 (5)	Al54—Al25—Al45	106.21 (6)
Al58^iii^—Cr2—Al59^i^	113.20 (5)	Al32^iii^—Al25—Al45	91.59 (6)
Al66^i^—Cr2—Al59^iii^	121.39 (5)	Al62—Al25—Al45	56.99 (5)
Al53^i^—Cr2—Al59^iii^	62.17 (5)	Al41^xvii^—Al25—Al45	145.74 (7)
Al57—Cr2—Al59^iii^	116.97 (5)	Al10^iii^—Al25—Al45	105.41 (6)
Al54—Cr2—Al59^iii^	62.66 (5)	Al6^iv^—Al25—Al45	57.25 (5)
Al55—Cr2—Al59^iii^	115.98 (5)	Cr12—Al25—Al22	59.26 (4)
Al56^i^—Cr2—Al59^iii^	174.85 (5)	Cr11—Al25—Al22	60.71 (4)
Cr2^ii^—Cr2—Al59^iii^	61.54 (4)	Al54—Al25—Al22	56.92 (5)
Al60^i^—Cr2—Al59^iii^	112.94 (4)	Al32^iii^—Al25—Al22	145.88 (7)
Al58^i^—Cr2—Al59^iii^	113.27 (5)	Al62—Al25—Al22	106.39 (6)
Al58^iii^—Cr2—Al59^iii^	62.14 (5)	Al41^xvii^—Al25—Al22	90.22 (6)
Al59^i^—Cr2—Al59^iii^	62.02 (6)	Al10^iii^—Al25—Al22	57.84 (5)
Al36^iv^—Cr3—Al36	125.47 (8)	Al6^iv^—Al25—Al22	106.66 (6)
Al36^iv^—Cr3—Al35^iv^	69.30 (5)	Al45—Al25—Al22	108.37 (6)
Al36—Cr3—Al35^iv^	68.70 (5)	Cr12—Al25—Al40^i^	110.92 (6)
Al36^iv^—Cr3—Al35	68.70 (5)	Cr11—Al25—Al40^i^	149.12 (7)
Al36—Cr3—Al35	69.30 (5)	Al54—Al25—Al40^i^	88.25 (6)
Al35^iv^—Cr3—Al35	77.07 (7)	Al32^iii^—Al25—Al40^i^	59.85 (5)
Al36^iv^—Cr3—Al13^iv^	112.49 (5)	Al62—Al25—Al40^i^	58.16 (5)
Al36—Cr3—Al13^iv^	114.20 (5)	Al41^xvii^—Al25—Al40^i^	90.46 (6)
Al35^iv^—Cr3—Al13^iv^	111.37 (4)	Al10^iii^—Al25—Al40^i^	147.12 (7)
Al35—Cr3—Al13^iv^	171.48 (5)	Al6^iv^—Al25—Al40^i^	109.97 (6)
Al36^iv^—Cr3—Al13	114.20 (5)	Al45—Al25—Al40^i^	90.86 (6)
Al36—Cr3—Al13	112.50 (5)	Al22—Al25—Al40^i^	143.35 (7)
Al35^iv^—Cr3—Al13	171.48 (5)	Cr12—Al25—Al59^iii^	110.45 (6)
Al35—Cr3—Al13	111.37 (4)	Cr11—Al25—Al59^iii^	149.92 (7)
Al13^iv^—Cr3—Al13	60.23 (6)	Al54—Al25—Al59^iii^	57.64 (5)
Al36^iv^—Cr3—Cr18	114.20 (5)	Al32^iii^—Al25—Al59^iii^	89.72 (6)
Al36—Cr3—Cr18	112.50 (5)	Al62—Al25—Al59^iii^	88.78 (5)
Al35^iv^—Cr3—Cr18	171.48 (5)	Al41^xvii^—Al25—Al59^iii^	60.77 (5)
Al35—Cr3—Cr18	111.37 (4)	Al10^iii^—Al25—Al59^iii^	110.21 (6)
Al36^iv^—Cr3—Al31^iv^	62.69 (4)	Al6^iv^—Al25—Al59^iii^	147.39 (7)
Al36—Cr3—Al31^iv^	171.40 (6)	Al45—Al25—Al59^iii^	144.34 (7)
Al35^iv^—Cr3—Al31^iv^	119.19 (4)	Al22—Al25—Al59^iii^	89.43 (6)
Al35—Cr3—Al31^iv^	114.65 (4)	Al40^i^—Al25—Al59^iii^	59.52 (5)
Al13^iv^—Cr3—Al31^iv^	60.79 (5)	Al2^xiv^—Al26—Al27	105.18 (6)
Al13—Cr3—Al31^iv^	59.16 (4)	Al2^xiv^—Al26—Cr5^xiv^	57.51 (4)
Al36^iv^—Cr3—Al31	171.40 (6)	Al27—Al26—Cr5^xiv^	104.11 (6)
Al36—Cr3—Al31	62.69 (4)	Al2^xiv^—Al26—Cr6^vii^	57.58 (4)
Al35^iv^—Cr3—Al31	114.65 (4)	Al27—Al26—Cr6^vii^	54.49 (4)
Al35—Cr3—Al31	119.19 (4)	Cr5^xiv^—Al26—Cr6^vii^	57.56 (4)
Al13^iv^—Cr3—Al31	59.16 (4)	Al2^xiv^—Al26—Al6	103.03 (6)
Al13—Cr3—Al31	60.79 (5)	Al27—Al26—Al6	119.54 (7)
Cr18—Cr3—Al31	60.79 (5)	Cr5^xiv^—Al26—Al6	53.13 (4)
Al31^iv^—Cr3—Al31	109.33 (7)	Cr6^vii^—Al26—Al6	103.83 (6)
Al36^iv^—Cr3—Al45^iv^	67.42 (4)	Al2^xiv^—Al26—Al35	99.93 (6)
Al36—Cr3—Al45^iv^	118.88 (5)	Al27—Al26—Al35	137.96 (7)
Al35^iv^—Cr3—Al45^iv^	128.82 (5)	Cr5^xiv^—Al26—Al35	117.82 (6)
Al35—Cr3—Al45^iv^	62.76 (4)	Cr6^vii^—Al26—Al35	156.76 (7)
Al13^iv^—Cr3—Al45^iv^	109.48 (5)	Al6—Al26—Al35	85.92 (6)
Al13—Cr3—Al45^iv^	58.64 (4)	Al2^xiv^—Al26—Al24^xiv^	58.78 (5)
Al31^iv^—Cr3—Al45^iv^	59.83 (4)	Al27—Al26—Al24^xiv^	160.70 (7)
Al31—Cr3—Al45^iv^	112.18 (5)	Cr5^xiv^—Al26—Al24^xiv^	59.10 (4)
Al36^iv^—Cr3—Al45	118.88 (5)	Cr6^vii^—Al26—Al24^xiv^	106.23 (6)
Al36—Cr3—Al45	67.42 (4)	Al6—Al26—Al24^xiv^	60.04 (5)
Al35^iv^—Cr3—Al45	62.76 (4)	Al35—Al26—Al24^xiv^	60.13 (5)
Al35—Cr3—Al45	128.82 (5)	Al2^xiv^—Al26—Al17^vii^	59.37 (5)
Al13^iv^—Cr3—Al45	58.64 (4)	Al27—Al26—Al17^vii^	61.82 (5)
Al13—Cr3—Al45	109.47 (5)	Cr5^xiv^—Al26—Al17^vii^	106.62 (6)
Cr18—Cr3—Al45	109.47 (5)	Cr6^vii^—Al26—Al17^vii^	59.26 (4)
Al31^iv^—Cr3—Al45	112.18 (5)	Al6—Al26—Al17^vii^	159.74 (7)
Al31—Cr3—Al45	59.83 (4)	Al35—Al26—Al17^vii^	106.00 (6)
Al45^iv^—Cr3—Al45	167.59 (7)	Al24^xiv^—Al26—Al17^vii^	111.33 (6)
Al36^iv^—Cr3—Al44	119.24 (5)	Al2^xiv^—Al26—Al27^vii^	99.21 (6)
Al36—Cr3—Al44	65.95 (4)	Al27—Al26—Al27^vii^	84.66 (6)
Al35^iv^—Cr3—Al44	126.99 (5)	Cr5^xiv^—Al26—Al27^vii^	156.40 (7)
Al35—Cr3—Al44	62.40 (4)	Cr6^vii^—Al26—Al27^vii^	115.65 (6)
Al13^iv^—Cr3—Al44	111.23 (5)	Al6—Al26—Al27^vii^	140.50 (7)
Al13—Cr3—Al44	59.00 (4)	Al35—Al26—Al27^vii^	58.15 (5)
Cr18—Cr3—Al44	59.00 (4)	Al24^xiv^—Al26—Al27^vii^	107.40 (6)
Al31^iv^—Cr3—Al44	108.46 (5)	Al17^vii^—Al26—Al27^vii^	57.85 (5)
Al31—Cr3—Al44	65.24 (4)	Al2^xiv^—Al26—Al39	103.03 (6)
Al45^iv^—Cr3—Al44	59.46 (4)	Al27—Al26—Al39	60.95 (5)
Al45—Cr3—Al44	119.27 (4)	Cr5^xiv^—Al26—Al39	56.08 (4)
Al34—Cr4—Al34^iv^	76.11 (7)	Cr6^vii^—Al26—Al39	56.63 (4)
Al34—Cr4—Al7^iv^	69.30 (5)	Al6—Al26—Al39	61.14 (5)
Al34^iv^—Cr4—Al7^iv^	68.70 (5)	Al35—Al26—Al39	143.17 (7)
Al34—Cr4—Al7	68.70 (5)	Al24^xiv^—Al26—Al39	109.62 (6)
Al34^iv^—Cr4—Al7	69.30 (5)	Al17^vii^—Al26—Al39	110.41 (6)
Al7^iv^—Cr4—Al7	125.86 (8)	Al27^vii^—Al26—Al39	142.77 (6)
Al34—Cr4—Al15^iv^	172.14 (5)	Al2^xiv^—Al26—Cr8^xiv^	54.32 (4)
Al34^iv^—Cr4—Al15^iv^	111.65 (4)	Al27—Al26—Cr8^xiv^	119.92 (6)
Al7^iv^—Cr4—Al15^iv^	114.05 (5)	Cr5^xiv^—Al26—Cr8^xiv^	104.97 (5)
Al7—Cr4—Al15^iv^	112.22 (5)	Cr6^vii^—Al26—Cr8^xiv^	103.95 (5)
Al34—Cr4—Al15	111.65 (4)	Al6—Al26—Cr8^xiv^	120.08 (6)
Al34^iv^—Cr4—Al15	172.14 (5)	Al35—Al26—Cr8^xiv^	53.57 (4)
Al7^iv^—Cr4—Al15	112.22 (5)	Al24^xiv^—Al26—Cr8^xiv^	61.60 (4)
Al7—Cr4—Al15	114.05 (5)	Al17^vii^—Al26—Cr8^xiv^	59.91 (4)
Al15^iv^—Cr4—Al15	60.62 (6)	Al27^vii^—Al26—Cr8^xiv^	52.70 (4)
Al34—Cr4—Cr19	111.65 (4)	Al39—Al26—Cr8^xiv^	157.34 (6)
Al34^iv^—Cr4—Cr19	172.14 (5)	Al2^xiv^—Al26—Al44	149.93 (7)
Al7^iv^—Cr4—Cr19	112.22 (5)	Al27—Al26—Al44	104.71 (6)
Al7—Cr4—Cr19	114.05 (5)	Cr5^xiv^—Al26—Al44	111.41 (6)
Al34—Cr4—Al33^iv^	119.22 (4)	Cr6^vii^—Al26—Al44	144.53 (6)
Al34^iv^—Cr4—Al33^iv^	114.58 (4)	Al6—Al26—Al44	58.42 (5)
Al7^iv^—Cr4—Al33^iv^	62.20 (4)	Al35—Al26—Al44	58.32 (5)
Al7—Cr4—Al33^iv^	171.46 (6)	Al24^xiv^—Al26—Al44	91.26 (6)
Al15^iv^—Cr4—Al33^iv^	59.47 (4)	Al17^vii^—Al26—Al44	141.82 (7)
Al15—Cr4—Al33^iv^	61.10 (5)	Al27^vii^—Al26—Al44	86.72 (6)
Al34—Cr4—Al33	114.58 (4)	Al39—Al26—Al44	88.66 (6)
Al34^iv^—Cr4—Al33	119.21 (4)	Cr8^xiv^—Al26—Al44	111.52 (6)
Al7^iv^—Cr4—Al33	171.47 (6)	Cr6^vii^—Al27—Cr8^i^	163.34 (7)
Al7—Cr4—Al33	62.20 (4)	Cr6^vii^—Al27—Al26	63.46 (5)
Al15^iv^—Cr4—Al33	61.10 (5)	Cr8^i^—Al27—Al26	132.11 (7)
Al15—Cr4—Al33	59.47 (4)	Cr6^vii^—Al27—Al35^vii^	113.23 (6)
Cr19—Cr4—Al33	59.47 (4)	Cr8^i^—Al27—Al35^vii^	57.78 (5)
Al33^iv^—Cr4—Al33	109.93 (7)	Al26—Al27—Al35^vii^	148.26 (7)
Al34—Cr4—Al37^iv^	62.70 (4)	Cr6^vii^—Al27—Al17	130.54 (6)
Al34^iv^—Cr4—Al37^iv^	128.22 (5)	Cr8^i^—Al27—Al17	65.20 (5)
Al7^iv^—Cr4—Al37^iv^	118.45 (5)	Al26—Al27—Al17	67.11 (5)
Al7—Cr4—Al37^iv^	67.42 (4)	Al35^vii^—Al27—Al17	109.35 (6)
Al15^iv^—Cr4—Al37^iv^	110.06 (5)	Cr6^vii^—Al27—Al42^i^	119.23 (6)
Al15—Cr4—Al37^iv^	58.78 (4)	Cr8^i^—Al27—Al42^i^	59.06 (5)
Al33^iv^—Cr4—Al37^iv^	112.40 (5)	Al26—Al27—Al42^i^	96.02 (6)
Al33—Cr4—Al37^iv^	60.15 (4)	Al35^vii^—Al27—Al42^i^	110.90 (6)
Al34—Cr4—Al37	128.22 (5)	Al17—Al27—Al42^i^	63.97 (5)
Al34^iv^—Cr4—Al37	62.70 (4)	Cr6^vii^—Al27—Al1	110.04 (5)
Al7^iv^—Cr4—Al37	67.42 (4)	Cr8^i^—Al27—Al1	53.90 (3)
Al7—Cr4—Al37	118.45 (5)	Al26—Al27—Al1	148.74 (7)
Al15^iv^—Cr4—Al37	58.78 (4)	Al35^vii^—Al27—Al1	62.97 (5)
Al15—Cr4—Al37	110.06 (5)	Al17—Al27—Al1	110.96 (6)
Cr19—Cr4—Al37	110.06 (5)	Al42^i^—Al27—Al1	59.04 (6)
Al33^iv^—Cr4—Al37	60.15 (4)	Cr6^vii^—Al27—Al39	59.75 (4)
Al33—Cr4—Al37	112.40 (5)	Cr8^i^—Al27—Al39	118.42 (6)
Al37^iv^—Cr4—Al37	168.34 (7)	Al26—Al27—Al39	62.82 (5)
Al34—Cr4—Al9	126.25 (5)	Al35^vii^—Al27—Al39	145.81 (7)
Al34^iv^—Cr4—Al9	62.60 (4)	Al17—Al27—Al39	96.12 (6)
Al7^iv^—Cr4—Al9	119.34 (5)	Al42^i^—Al27—Al39	60.11 (5)
Al7—Cr4—Al9	65.52 (4)	Al1—Al27—Al39	87.12 (6)
Al15^iv^—Cr4—Al9	59.16 (4)	Cr6^vii^—Al27—Al17^vii^	61.74 (4)
Al15—Cr4—Al9	111.66 (5)	Cr8^i^—Al27—Al17^vii^	127.74 (6)
Cr19—Cr4—Al9	111.66 (5)	Al26—Al27—Al17^vii^	61.35 (5)
Al33^iv^—Cr4—Al9	108.86 (5)	Al35^vii^—Al27—Al17^vii^	88.68 (6)
Al33—Cr4—Al9	65.24 (4)	Al17—Al27—Al17^vii^	96.00 (6)
Al37^iv^—Cr4—Al9	119.46 (4)	Al42^i^—Al27—Al17^vii^	155.29 (7)
Al37—Cr4—Al9	59.42 (4)	Al1—Al27—Al17^vii^	145.64 (7)
Al6^v^—Cr5—Al3^vi^	71.27 (5)	Al39—Al27—Al17^vii^	111.50 (6)
Al6^v^—Cr5—Al5	175.13 (6)	Cr6^vii^—Al27—Al26^vii^	125.22 (6)
Al3^vi^—Cr5—Al5	113.47 (5)	Cr8^i^—Al27—Al26^vii^	64.64 (5)
Al6^v^—Cr5—Cr16	175.13 (6)	Al26—Al27—Al26^vii^	95.34 (6)
Al3^vi^—Cr5—Cr16	113.47 (5)	Al35^vii^—Al27—Al26^vii^	59.52 (5)
Al6^v^—Cr5—Al2	114.42 (5)	Al17—Al27—Al26^vii^	60.58 (5)
Al3^vi^—Cr5—Al2	174.20 (6)	Al42^i^—Al27—Al26^vii^	112.47 (6)
Al5—Cr5—Al2	60.81 (5)	Al1—Al27—Al26^vii^	110.99 (6)
Cr16—Cr5—Al2	60.81 (5)	Al39—Al27—Al26^vii^	154.01 (7)
Al6^v^—Cr5—Cr7^vi^	118.42 (5)	Al17^vii^—Al27—Al26^vii^	63.81 (5)
Al3^vi^—Cr5—Cr7^vi^	119.19 (4)	Cr6^vii^—Al27—Al36^vii^	53.25 (4)
Al5—Cr5—Cr7^vi^	60.78 (4)	Cr8^i^—Al27—Al36^vii^	116.40 (6)
Al2—Cr5—Cr7^vi^	59.76 (4)	Al26—Al27—Al36^vii^	107.79 (6)
Al6^v^—Cr5—Cr6^i^	116.87 (4)	Al35^vii^—Al27—Al36^vii^	60.01 (5)
Al3^vi^—Cr5—Cr6^i^	117.48 (4)	Al17—Al27—Al36^vii^	152.78 (7)
Al5—Cr5—Cr6^i^	60.41 (4)	Al42^i^—Al27—Al36^vii^	142.11 (7)
Al2—Cr5—Cr6^i^	59.58 (4)	Al1—Al27—Al36^vii^	87.24 (6)
Cr7^vi^—Cr5—Cr6^i^	109.44 (4)	Al39—Al27—Al36^vii^	105.15 (6)
Al6^v^—Cr5—Al39^v^	68.67 (5)	Al17^vii^—Al27—Al36^vii^	60.63 (5)
Al3^vi^—Cr5—Al39^v^	68.51 (5)	Al26^vii^—Al27—Al36^vii^	94.56 (6)
Al5—Cr5—Al39^v^	111.54 (5)	Al5—Al28—Cr6^i^	57.62 (4)
Al2—Cr5—Al39^v^	111.93 (5)	Al5—Al28—Al50^i^	150.55 (7)
Cr7^vi^—Cr5—Al39^v^	170.31 (5)	Cr6^i^—Al28—Al50^i^	140.91 (7)
Cr6^i^—Cr5—Al39^v^	60.88 (4)	Al5—Al28—Al36^i^	104.52 (6)
Al6^v^—Cr5—Al32^vi^	69.16 (5)	Cr6^i^—Al28—Al36^i^	53.32 (4)
Al3^vi^—Cr5—Al32^vi^	69.19 (5)	Al50^i^—Al28—Al36^i^	103.94 (6)
Al5—Cr5—Al32^vi^	113.12 (5)	Cr16—Al28—Al16	58.36 (5)
Al2—Cr5—Al32^vi^	113.31 (5)	Al5—Al28—Al16	58.36 (5)
Cr7^vi^—Cr5—Al32^vi^	62.30 (4)	Cr6^i^—Al28—Al16	61.30 (4)
Cr6^i^—Cr5—Al32^vi^	171.73 (5)	Al50^i^—Al28—Al16	144.20 (7)
Al39^v^—Cr5—Al32^vi^	127.38 (5)	Al36^i^—Al28—Al16	64.76 (5)
Al6^v^—Cr5—Al24	64.82 (5)	Cr16—Al28—Al2	56.50 (5)
Al3^vi^—Cr5—Al24	124.82 (5)	Al5—Al28—Al2	56.50 (5)
Al5—Cr5—Al24	111.83 (5)	Cr6^i^—Al28—Al2	56.23 (4)
Cr16—Cr5—Al24	111.83 (5)	Al50^i^—Al28—Al2	109.89 (6)
Al2—Cr5—Al24	60.35 (5)	Al36^i^—Al28—Al2	101.90 (6)
Cr7^vi^—Cr5—Al24	61.77 (4)	Al16—Al28—Al2	105.76 (6)
Cr6^i^—Cr5—Al24	111.42 (4)	Cr16—Al28—Al48	99.21 (6)
Al39^v^—Cr5—Al24	119.89 (5)	Al5—Al28—Al48	99.21 (6)
Al32^vi^—Cr5—Al24	65.14 (5)	Cr6^i^—Al28—Al48	156.66 (7)
Al6^v^—Cr5—Al26^v^	64.51 (5)	Al50^i^—Al28—Al48	61.05 (5)
Al3^vi^—Cr5—Al26^v^	124.34 (5)	Al36^i^—Al28—Al48	142.31 (7)
Al5—Cr5—Al26^v^	110.96 (5)	Al16—Al28—Al48	105.90 (6)
Al2—Cr5—Al26^v^	59.56 (5)	Al2—Al28—Al48	115.63 (6)
Cr7^vi^—Cr5—Al26^v^	110.92 (4)	Cr16—Al28—Cr13	55.17 (4)
Cr6^i^—Cr5—Al26^v^	61.39 (4)	Al5—Al28—Cr13	55.17 (4)
Al39^v^—Cr5—Al26^v^	65.09 (5)	Cr6^i^—Al28—Cr13	106.39 (5)
Al32^vi^—Cr5—Al26^v^	119.90 (5)	Al50^i^—Al28—Cr13	112.59 (6)
Al24—Cr5—Al26^v^	61.69 (4)	Al36^i^—Al28—Cr13	123.96 (6)
Al6^v^—Cr5—Al19	124.01 (5)	Al16—Al28—Cr13	60.60 (4)
Al3^vi^—Cr5—Al19	63.90 (5)	Al2—Al28—Cr13	103.66 (5)
Al5—Cr5—Al19	60.43 (5)	Al48—Al28—Cr13	51.86 (4)
Cr16—Cr5—Al19	60.43 (5)	Al5—Al28—Al62^i^	99.43 (6)
Al2—Cr5—Al19	111.95 (5)	Cr6^i^—Al28—Al62^i^	117.27 (6)
Cr7^vi^—Cr5—Al19	63.58 (4)	Al50^i^—Al28—Al62^i^	89.17 (6)
Cr6^i^—Cr5—Al19	112.75 (4)	Al36^i^—Al28—Al62^i^	87.55 (6)
Al39^v^—Cr5—Al19	119.08 (5)	Al16—Al28—Al62^i^	57.77 (5)
Al32^vi^—Cr5—Al19	64.82 (5)	Al2—Al28—Al62^i^	155.54 (7)
Al24—Cr5—Al19	117.82 (5)	Al48—Al28—Al62^i^	59.69 (5)
Al26^v^—Cr5—Al19	171.06 (5)	Cr13—Al28—Al62^i^	53.42 (4)
Al36—Cr6—Al27^vii^	71.95 (5)	Cr16—Al28—Al29	57.61 (5)
Al36—Cr6—Al30^viii^	70.82 (5)	Al5—Al28—Al29	57.61 (5)
Al27^vii^—Cr6—Al30^viii^	75.10 (5)	Cr6^i^—Al28—Al29	104.94 (6)
Al36—Cr6—Al2^i^	116.70 (5)	Al50^i^—Al28—Al29	92.95 (6)
Al27^vii^—Cr6—Al2^i^	113.28 (5)	Al36^i^—Al28—Al29	158.21 (7)
Al30^viii^—Cr6—Al2^i^	169.74 (5)	Al16—Al28—Al29	108.48 (6)
Al36—Cr6—Al5^i^	116.36 (5)	Al2—Al28—Al29	58.73 (5)
Al27^vii^—Cr6—Al5^i^	170.67 (5)	Al48—Al28—Al29	58.55 (5)
Al30^viii^—Cr6—Al5^i^	111.02 (5)	Cr13—Al28—Al29	58.78 (4)
Al2^i^—Cr6—Al5^i^	59.91 (4)	Al62^i^—Al28—Al29	106.63 (6)
Al36—Cr6—Cr5^i^	174.00 (5)	Cr16—Al29—Al7	104.90 (6)
Al27^vii^—Cr6—Cr5^i^	113.46 (5)	Al5—Al29—Al7	104.90 (6)
Al30^viii^—Cr6—Cr5^i^	112.57 (4)	Cr16—Al29—Al51	150.82 (7)
Al2^i^—Cr6—Cr5^i^	59.31 (4)	Al5—Al29—Al51	150.82 (7)
Al5^i^—Cr6—Cr5^i^	58.05 (4)	Al7—Al29—Al51	103.31 (6)
Al36—Cr6—Al39^vii^	125.96 (5)	Al5—Al29—Cr7^vi^	57.18 (4)
Al27^vii^—Cr6—Al39^vii^	66.68 (5)	Al7—Al29—Cr7^vi^	54.19 (4)
Al30^viii^—Cr6—Al39^vii^	66.20 (5)	Al51—Al29—Cr7^vi^	141.05 (7)
Al2^i^—Cr6—Al39^vii^	110.81 (5)	Cr16—Al29—Al14	58.19 (5)
Al5^i^—Cr6—Al39^vii^	108.70 (5)	Al5—Al29—Al14	58.19 (5)
Cr5^i^—Cr6—Al39^vii^	59.83 (4)	Al7—Al29—Al14	65.06 (5)
Al36—Cr6—Al28^i^	63.77 (5)	Al51—Al29—Al14	144.20 (7)
Al27^vii^—Cr6—Al28^i^	123.42 (5)	Cr7^vi^—Al29—Al14	61.25 (5)
Al30^viii^—Cr6—Al28^i^	117.81 (5)	Cr16—Al29—Al48	99.27 (6)
Al2^i^—Cr6—Al28^i^	63.37 (5)	Al5—Al29—Al48	99.27 (6)
Al5^i^—Cr6—Al28^i^	60.80 (4)	Al7—Al29—Al48	141.85 (7)
Cr5^i^—Cr6—Al28^i^	110.33 (4)	Al51—Al29—Al48	61.23 (5)
Al39^vii^—Cr6—Al28^i^	169.35 (5)	Cr7^vi^—Al29—Al48	156.29 (7)
Al36—Cr6—Al26^vii^	121.73 (5)	Al14—Al29—Al48	105.84 (6)
Al27^vii^—Cr6—Al26^vii^	62.05 (5)	Cr16—Al29—Al2	56.41 (5)
Al30^viii^—Cr6—Al26^vii^	123.94 (5)	Al5—Al29—Al2	56.41 (5)
Al2^i^—Cr6—Al26^vii^	59.36 (5)	Al7—Al29—Al2	102.08 (6)
Al5^i^—Cr6—Al26^vii^	108.76 (5)	Al51—Al29—Al2	110.17 (6)
Cr5^i^—Cr6—Al26^vii^	61.05 (4)	Cr7^vi^—Al29—Al2	55.73 (4)
Al39^vii^—Cr6—Al26^vii^	64.61 (5)	Al14—Al29—Al2	105.46 (6)
Al28^i^—Cr6—Al26^vii^	115.55 (5)	Al48—Al29—Al2	115.88 (6)
Al36—Cr6—Al17	67.51 (5)	Cr16—Al29—Cr13	55.21 (4)
Al27^vii^—Cr6—Al17	65.49 (5)	Al5—Al29—Cr13	55.21 (4)
Al30^viii^—Cr6—Al17	129.24 (5)	Al7—Al29—Cr13	123.69 (6)
Al2^i^—Cr6—Al17	60.98 (4)	Al51—Al29—Cr13	112.91 (6)
Al5^i^—Cr6—Al17	112.68 (5)	Cr7^vi^—Al29—Cr13	105.94 (5)
Cr5^i^—Cr6—Al17	111.77 (4)	Al14—Al29—Cr13	60.20 (4)
Al39^vii^—Cr6—Al17	120.01 (5)	Al48—Al29—Cr13	52.05 (4)
Al28^i^—Cr6—Al17	66.10 (5)	Al2—Al29—Cr13	103.87 (5)
Al26^vii^—Cr6—Al17	62.11 (5)	Cr16—Al29—Al61	99.48 (6)
Al36—Cr6—Al16^i^	68.06 (5)	Al5—Al29—Al61	99.48 (6)
Al27^vii^—Cr6—Al16^i^	130.20 (5)	Al7—Al29—Al61	87.27 (6)
Al30^viii^—Cr6—Al16^i^	64.68 (5)	Al51—Al29—Al61	89.10 (6)
Al2^i^—Cr6—Al16^i^	110.55 (5)	Cr7^vi^—Al29—Al61	117.37 (6)
Al5^i^—Cr6—Al16^i^	58.97 (4)	Al14—Al29—Al61	57.86 (5)
Cr5^i^—Cr6—Al16^i^	108.48 (4)	Al48—Al29—Al61	59.65 (5)
Al39^vii^—Cr6—Al16^i^	117.12 (5)	Al2—Al29—Al61	155.54 (7)
Al28^i^—Cr6—Al16^i^	60.27 (4)	Cr13—Al29—Al61	53.24 (4)
Al26^vii^—Cr6—Al16^i^	167.72 (5)	Cr16—Al29—Al28	57.37 (5)
Al17—Cr6—Al16^i^	120.92 (5)	Al5—Al29—Al28	57.37 (5)
Al36—Cr6—Al18^i^	119.46 (5)	Al7—Al29—Al28	158.38 (7)
Al27^vii^—Cr6—Al18^i^	122.82 (5)	Al51—Al29—Al28	93.46 (6)
Al30^viii^—Cr6—Al18^i^	60.91 (5)	Cr7^vi^—Al29—Al28	104.24 (6)
Al2^i^—Cr6—Al18^i^	108.85 (5)	Al14—Al29—Al28	108.15 (6)
Al5^i^—Cr6—Al18^i^	58.05 (4)	Al48—Al29—Al28	58.86 (5)
Cr5^i^—Cr6—Al18^i^	60.44 (4)	Al2—Al29—Al28	58.69 (5)
Al39^vii^—Cr6—Al18^i^	63.24 (4)	Cr13—Al29—Al28	59.10 (4)
Al28^i^—Cr6—Al18^i^	109.18 (5)	Al61—Al29—Al28	106.79 (6)
Al26^vii^—Cr6—Al18^i^	114.93 (5)	Cr6^xviii^—Al30—Cr8	163.20 (7)
Al17—Cr6—Al18^i^	169.75 (5)	Cr6^xviii^—Al30—Al18^xv^	65.22 (5)
Al16^i^—Cr6—Al18^i^	59.73 (4)	Cr8—Al30—Al18^xv^	130.44 (7)
Al23^vi^—Cr7—Al7^vi^	71.47 (5)	Cr6^xviii^—Al30—Al8	131.26 (6)
Al23^vi^—Cr7—Al20	73.60 (5)	Cr8—Al30—Al8	64.62 (5)
Al7^vi^—Cr7—Al20	70.35 (5)	Al18^xv^—Al30—Al8	66.06 (5)
Al23^vi^—Cr7—Al2^vi^	114.10 (5)	Cr6^xviii^—Al30—Al35^v^	113.24 (6)
Al7^vi^—Cr7—Al2^vi^	115.15 (5)	Cr8—Al30—Al35^v^	57.60 (4)
Al20—Cr7—Al2^vi^	171.34 (5)	Al18^xv^—Al30—Al35^v^	149.01 (7)
Al23^vi^—Cr7—Cr5^vi^	114.77 (5)	Al8—Al30—Al35^v^	108.84 (6)
Al7^vi^—Cr7—Cr5^vi^	172.94 (5)	Cr6^xviii^—Al30—Al43	119.36 (6)
Al20—Cr7—Cr5^vi^	113.94 (5)	Cr8—Al30—Al43	58.95 (4)
Al2^vi^—Cr7—Cr5^vi^	59.91 (4)	Al18^xv^—Al30—Al43	94.79 (6)
Al23^vi^—Cr7—Al5^vi^	172.41 (5)	Al8—Al30—Al43	63.76 (5)
Al7^vi^—Cr7—Al5^vi^	114.98 (5)	Al35^v^—Al30—Al43	110.56 (6)
Al20—Cr7—Al5^vi^	111.84 (5)	Cr6^xviii^—Al30—Al39^iii^	59.89 (4)
Al2^vi^—Cr7—Al5^vi^	60.11 (4)	Cr8—Al30—Al39^iii^	118.43 (6)
Cr5^vi^—Cr7—Al5^vi^	58.55 (4)	Al18^xv^—Al30—Al39^iii^	62.62 (5)
Al23^vi^—Cr7—Al32	67.44 (5)	Al8—Al30—Al39^iii^	96.16 (6)
Al7^vi^—Cr7—Al32	127.06 (5)	Al35^v^—Al30—Al39^iii^	145.95 (7)
Al20—Cr7—Al32	67.14 (5)	Al43—Al30—Al39^iii^	60.17 (5)
Al2^vi^—Cr7—Al32	111.33 (5)	Cr6^xviii^—Al30—Al1^i^	109.80 (5)
Cr5^vi^—Cr7—Al32	59.82 (4)	Cr8—Al30—Al1^i^	54.01 (3)
Al5^vi^—Cr7—Al32	109.20 (5)	Al18^xv^—Al30—Al1^i^	148.33 (7)
Al23^vi^—Cr7—Al29^vi^	122.31 (5)	Al8—Al30—Al1^i^	110.65 (6)
Al7^vi^—Cr7—Al29^vi^	62.20 (5)	Al35^v^—Al30—Al1^i^	62.65 (5)
Al20—Cr7—Al29^vi^	116.80 (5)	Al43—Al30—Al1^i^	59.14 (6)
Al2^vi^—Cr7—Al29^vi^	63.24 (4)	Al39^iii^—Al30—Al1^i^	87.38 (6)
Cr5^vi^—Cr7—Al29^vi^	110.81 (4)	Cr6^xviii^—Al30—Al16^xv^	62.66 (4)
Al5^vi^—Cr7—Al29^vi^	60.74 (4)	Cr8—Al30—Al16^xv^	127.57 (6)
Al32—Cr7—Al29^vi^	169.82 (5)	Al18^xv^—Al30—Al16^xv^	60.33 (5)
Al23^vi^—Cr7—Al24^vi^	62.71 (5)	Al8—Al30—Al16^xv^	93.99 (6)
Al7^vi^—Cr7—Al24^vi^	121.58 (5)	Al35^v^—Al30—Al16^xv^	90.70 (6)
Al20—Cr7—Al24^vi^	123.54 (5)	Al43—Al30—Al16^xv^	152.81 (7)
Al2^vi^—Cr7—Al24^vi^	60.28 (5)	Al39^iii^—Al30—Al16^xv^	110.70 (6)
Cr5^vi^—Cr7—Al24^vi^	61.45 (4)	Al1^i^—Al30—Al16^xv^	147.97 (7)
Al5^vi^—Cr7—Al24^vi^	109.75 (5)	Cr6^xviii^—Al30—Al36^xviii^	53.68 (4)
Al32—Cr7—Al24^vi^	64.18 (5)	Cr8—Al30—Al36^xviii^	116.20 (6)
Al29^vi^—Cr7—Al24^vi^	116.20 (5)	Al18^xv^—Al30—Al36^xviii^	109.22 (6)
Al23^vi^—Cr7—Al8^vi^	65.32 (5)	Al8—Al30—Al36^xviii^	151.49 (7)
Al7^vi^—Cr7—Al8^vi^	66.60 (5)	Al35^v^—Al30—Al36^xviii^	59.67 (5)
Al20—Cr7—Al8^vi^	127.45 (5)	Al43—Al30—Al36^xviii^	143.30 (7)
Al2^vi^—Cr7—Al8^vi^	61.01 (4)	Al39^iii^—Al30—Al36^xviii^	106.47 (6)
Cr5^vi^—Cr7—Al8^vi^	112.29 (4)	Al1^i^—Al30—Al36^xviii^	87.90 (6)
Al5^vi^—Cr7—Al8^vi^	112.75 (5)	Al16^xv^—Al30—Al36^xviii^	62.13 (5)
Al32—Cr7—Al8^vi^	120.09 (5)	Cr6^xviii^—Al30—Al24	125.02 (6)
Al29^vi^—Cr7—Al8^vi^	65.90 (5)	Cr8—Al30—Al24	64.71 (5)
Al24^vi^—Cr7—Al8^vi^	62.53 (5)	Al18^xv^—Al30—Al24	95.20 (6)
Al23^vi^—Cr7—Al14^vi^	128.69 (5)	Al8—Al30—Al24	60.92 (5)
Al7^vi^—Cr7—Al14^vi^	67.41 (5)	Al35^v^—Al30—Al24	59.17 (5)
Al20—Cr7—Al14^vi^	64.69 (5)	Al43—Al30—Al24	112.78 (6)
Al2^vi^—Cr7—Al14^vi^	110.42 (5)	Al39^iii^—Al30—Al24	154.21 (7)
Cr5^vi^—Cr7—Al14^vi^	108.79 (4)	Al1^i^—Al30—Al24	110.82 (6)
Al5^vi^—Cr7—Al14^vi^	58.84 (4)	Al16^xv^—Al30—Al24	63.13 (5)
Al32—Cr7—Al14^vi^	117.39 (5)	Al36^xviii^—Al30—Al24	92.76 (6)
Al29^vi^—Cr7—Al14^vi^	60.04 (4)	Al13^iv^—Al31—Al13	56.99 (6)
Al24^vi^—Cr7—Al14^vi^	168.59 (5)	Al13^iv^—Al31—Cr12	57.24 (4)
Al8^vi^—Cr7—Al14^vi^	120.37 (5)	Cr18—Al31—Cr12	107.09 (6)
Al23^vi^—Cr7—Al19^vi^	122.48 (5)	Al13—Al31—Cr12	107.09 (6)
Al7^vi^—Cr7—Al19^vi^	119.48 (5)	Al13^iv^—Al31—Al50	154.20 (8)
Al20—Cr7—Al19^vi^	61.74 (5)	Cr18—Al31—Al50	111.30 (6)
Al2^vi^—Cr7—Al19^vi^	109.75 (5)	Al13—Al31—Al50	111.30 (6)
Cr5^vi^—Cr7—Al19^vi^	60.73 (4)	Cr12—Al31—Al50	113.87 (6)
Al5^vi^—Cr7—Al19^vi^	58.79 (4)	Al13^iv^—Al31—Al36	102.24 (6)
Al32—Cr7—Al19^vi^	63.02 (4)	Cr18—Al31—Al36	99.22 (6)
Al29^vi^—Cr7—Al19^vi^	109.72 (5)	Al13—Al31—Al36	99.22 (6)
Al24^vi^—Cr7—Al19^vi^	115.07 (5)	Cr12—Al31—Al36	121.94 (6)
Al8^vi^—Cr7—Al19^vi^	170.68 (5)	Al50—Al31—Al36	102.44 (6)
Al14^vi^—Cr7—Al19^vi^	60.08 (4)	Al13^iv^—Al31—Cr3	55.77 (4)
Al1^i^—Cr8—Al2	179.12 (6)	Cr18—Al31—Cr3	55.10 (4)
Al1^i^—Cr8—Al30	69.02 (4)	Al13—Al31—Cr3	55.10 (4)
Al2—Cr8—Al30	111.53 (5)	Cr12—Al31—Cr3	105.76 (5)
Al1^i^—Cr8—Al27^i^	68.76 (4)	Al50—Al31—Cr3	140.35 (6)
Al2—Cr8—Al27^i^	110.38 (5)	Al36—Al31—Cr3	52.48 (4)
Al30—Cr8—Al27^i^	122.36 (5)	Al13^iv^—Al31—Al45	57.84 (5)
Al1^i^—Cr8—Al35^v^	70.86 (6)	Cr18—Al31—Al45	105.32 (6)
Al2—Cr8—Al35^v^	108.68 (5)	Al13—Al31—Al45	105.32 (6)
Al30—Cr8—Al35^v^	65.56 (5)	Cr12—Al31—Al45	59.52 (4)
Al27^i^—Cr8—Al35^v^	64.67 (5)	Al50—Al31—Al45	142.68 (7)
Al1^i^—Cr8—Al43	65.91 (5)	Al36—Al31—Al45	64.00 (5)
Al2—Cr8—Al43	114.91 (5)	Cr3—Al31—Al45	60.40 (4)
Al30—Cr8—Al43	64.82 (5)	Al13^iv^—Al31—Al52	100.95 (6)
Al27^i^—Cr8—Al43	125.72 (5)	Cr18—Al31—Al52	118.78 (7)
Al35^v^—Cr8—Al43	122.59 (5)	Al13—Al31—Al52	118.78 (7)
Al1^i^—Cr8—Al42	65.60 (5)	Cr12—Al31—Al52	51.87 (4)
Al2—Cr8—Al42	114.27 (5)	Al50—Al31—Al52	62.70 (5)
Al30—Cr8—Al42	125.85 (5)	Al36—Al31—Al52	141.88 (7)
Al27^i^—Cr8—Al42	64.38 (5)	Cr3—Al31—Al52	156.31 (6)
Al35^v^—Cr8—Al42	121.57 (5)	Al45—Al31—Al52	105.33 (6)
Al43—Cr8—Al42	70.69 (5)	Al13^iv^—Al31—Al62	100.62 (6)
Al1^i^—Cr8—Al63	117.95 (7)	Cr18—Al31—Al62	157.52 (7)
Al2—Cr8—Al63	62.49 (5)	Al13—Al31—Al62	157.52 (7)
Al30—Cr8—Al63	117.15 (5)	Cr12—Al31—Al62	52.65 (4)
Al27^i^—Cr8—Al63	116.57 (5)	Al50—Al31—Al62	88.18 (6)
Al35^v^—Cr8—Al63	171.16 (6)	Al36—Al31—Al62	86.80 (6)
Al43—Cr8—Al63	64.53 (5)	Cr3—Al31—Al62	116.28 (6)
Al42—Cr8—Al63	64.52 (5)	Al45—Al31—Al62	57.87 (5)
Al1^i^—Cr8—Al8	120.26 (4)	Al52—Al31—Al62	59.36 (5)
Al2—Cr8—Al8	60.58 (4)	Cr18—Al31—Cr15	61.21 (5)
Al30—Cr8—Al8	61.19 (5)	Cr12—Al31—Cr15	64.00 (4)
Al27^i^—Cr8—Al8	169.76 (5)	Al50—Al31—Cr15	90.99 (6)
Al35^v^—Cr8—Al8	112.24 (5)	Al36—Al31—Cr15	159.59 (7)
Al43—Cr8—Al8	64.44 (5)	Cr3—Al31—Cr15	107.71 (5)
Al42—Cr8—Al8	122.60 (5)	Al45—Al31—Cr15	113.22 (6)
Al63—Cr8—Al8	64.87 (5)	Al52—Al31—Cr15	58.24 (5)
Al1^i^—Cr8—Al17^i^	119.50 (4)	Al62—Al31—Cr15	109.18 (6)
Al2—Cr8—Al17^i^	59.88 (4)	Al13^iv^—Al31—Al4	63.22 (5)
Al30—Cr8—Al17^i^	170.05 (5)	Al13—Al31—Al4	61.21 (5)
Al27^i^—Cr8—Al17^i^	60.65 (5)	Cr12—Al31—Al4	64.00 (4)
Al35^v^—Cr8—Al17^i^	111.08 (5)	Al50—Al31—Al4	90.99 (6)
Al43—Cr8—Al17^i^	122.31 (5)	Al36—Al31—Al4	159.59 (7)
Al42—Cr8—Al17^i^	64.07 (5)	Cr3—Al31—Al4	107.71 (5)
Al63—Cr8—Al17^i^	64.72 (5)	Al45—Al31—Al4	113.22 (6)
Al8—Cr8—Al17^i^	114.12 (5)	Al52—Al31—Al4	58.24 (5)
Al1^i^—Cr8—Al26^v^	121.74 (6)	Al62—Al31—Al4	109.18 (6)
Al2—Cr8—Al26^v^	57.46 (4)	Cr5^vi^—Al32—Cr7	57.88 (4)
Al30—Cr8—Al26^v^	113.10 (5)	Cr5^vi^—Al32—Al25^x^	112.22 (6)
Al27^i^—Cr8—Al26^v^	62.67 (5)	Cr7—Al32—Al25^x^	149.02 (6)
Al35^v^—Cr8—Al26^v^	60.70 (5)	Cr5^vi^—Al32—Al41^iv^	112.16 (6)
Al43—Cr8—Al26^v^	171.48 (5)	Cr7—Al32—Al41^iv^	149.69 (6)
Al42—Cr8—Al26^v^	115.09 (5)	Al25^x^—Al32—Al41^iv^	60.12 (5)
Al63—Cr8—Al26^v^	111.47 (5)	Cr5^vi^—Al32—Al38	148.81 (7)
Al8—Cr8—Al26^v^	107.14 (5)	Cr7—Al32—Al38	111.08 (6)
Al17^i^—Cr8—Al26^v^	58.72 (4)	Al25^x^—Al32—Al38	91.13 (6)
Al1^i^—Cr8—Al24	122.05 (6)	Al41^iv^—Al32—Al38	60.98 (5)
Al2—Cr8—Al24	58.00 (4)	Cr5^vi^—Al32—Al40^xi^	148.91 (7)
Al30—Cr8—Al24	63.31 (5)	Cr7—Al32—Al40^xi^	110.57 (6)
Al27^i^—Cr8—Al24	112.28 (5)	Al25^x^—Al32—Al40^xi^	61.14 (5)
Al35^v^—Cr8—Al24	60.79 (5)	Al41^iv^—Al32—Al40^xi^	91.24 (6)
Al43—Cr8—Al24	116.30 (5)	Al38—Al32—Al40^xi^	60.47 (5)
Al42—Cr8—Al24	170.82 (5)	Cr5^vi^—Al32—Al20	100.55 (6)
Al63—Cr8—Al24	112.11 (5)	Cr7—Al32—Al20	52.49 (4)
Al8—Cr8—Al24	59.55 (4)	Al25^x^—Al32—Al20	147.22 (7)
Al17^i^—Cr8—Al24	106.76 (4)	Al41^iv^—Al32—Al20	109.01 (6)
Al26^v^—Cr8—Al24	57.28 (4)	Al38—Al32—Al20	59.10 (5)
Al12—Cr9—Al34^vi^	72.73 (6)	Al40^xi^—Al32—Al20	90.11 (5)
Al12—Cr9—Al20	69.30 (4)	Cr5^vi^—Al32—Al23^vi^	100.82 (6)
Al34^vi^—Cr9—Al20	67.52 (5)	Cr7—Al32—Al23^vi^	52.26 (4)
Al12—Cr9—Al23^ix^	69.07 (4)	Al25^x^—Al32—Al23^vi^	108.86 (6)
Al34^vi^—Cr9—Al23^ix^	66.88 (5)	Al41^iv^—Al32—Al23^vi^	147.00 (6)
Al20—Cr9—Al23^ix^	124.95 (5)	Al38—Al32—Al23^vi^	90.10 (6)
Al12—Cr9—Al5	175.95 (7)	Al40^xi^—Al32—Al23^vi^	58.75 (5)
Al34^vi^—Cr9—Al5	111.24 (5)	Al20—Al32—Al23^vi^	61.90 (5)
Al20—Cr9—Al5	112.63 (5)	Cr5^vi^—Al32—Al19^vi^	59.12 (4)
Al23^ix^—Cr9—Al5	111.42 (5)	Cr7—Al32—Al19^vi^	60.19 (4)
Al12—Cr9—Cr16	175.95 (7)	Al25^x^—Al32—Al19^vi^	144.36 (7)
Al34^vi^—Cr9—Cr16	111.24 (5)	Al41^iv^—Al32—Al19^vi^	89.79 (6)
Al20—Cr9—Cr16	112.63 (5)	Al38—Al32—Al19^vi^	89.81 (6)
Al23^ix^—Cr9—Cr16	111.42 (5)	Al40^xi^—Al32—Al19^vi^	144.74 (7)
Al12—Cr9—Cr13	116.44 (6)	Al20—Al32—Al19^vi^	56.51 (5)
Al34^vi^—Cr9—Cr13	170.80 (5)	Al23^vi^—Al32—Al19^vi^	106.77 (6)
Al20—Cr9—Cr13	115.32 (4)	Cr5^vi^—Al32—Al24^vi^	58.70 (4)
Al23^ix^—Cr9—Cr13	114.69 (4)	Cr7—Al32—Al24^vi^	58.25 (4)
Al5—Cr9—Cr13	59.58 (4)	Al25^x^—Al32—Al24^vi^	91.09 (6)
Cr16—Cr9—Cr13	59.58 (4)	Al41^iv^—Al32—Al24^vi^	145.50 (7)
Al12—Cr9—Al38	65.56 (5)	Al38—Al32—Al24^vi^	144.75 (7)
Al34^vi^—Cr9—Al38	125.48 (5)	Al40^xi^—Al32—Al24^vi^	90.37 (6)
Al20—Cr9—Al38	65.92 (5)	Al20—Al32—Al24^vi^	105.45 (6)
Al23^ix^—Cr9—Al38	123.13 (5)	Al23^vi^—Al32—Al24^vi^	56.09 (5)
Al5—Cr9—Al38	111.65 (5)	Al19^vi^—Al32—Al24^vi^	108.05 (6)
Cr16—Cr9—Al38	111.65 (5)	Cr5^vi^—Al32—Al6^xiii^	52.41 (4)
Cr13—Cr9—Al38	62.03 (4)	Cr7—Al32—Al6^xiii^	101.28 (5)
Al12—Cr9—Al40	65.45 (5)	Al25^x^—Al32—Al6^xiii^	60.08 (5)
Al34^vi^—Cr9—Al40	124.75 (5)	Al41^iv^—Al32—Al6^xiii^	89.86 (6)
Al20—Cr9—Al40	123.46 (5)	Al38—Al32—Al6^xiii^	147.57 (7)
Al23^ix^—Cr9—Al40	65.47 (5)	Al40^xi^—Al32—Al6^xiii^	110.28 (6)
Al5—Cr9—Al40	110.89 (5)	Al20—Al32—Al6^xiii^	152.19 (7)
Cr16—Cr9—Al40	110.89 (5)	Al23^vi^—Al32—Al6^xiii^	112.11 (6)
Cr13—Cr9—Al40	61.88 (4)	Al19^vi^—Al32—Al6^xiii^	104.97 (6)
Al38—Cr9—Al40	65.58 (5)	Al24^vi^—Al32—Al6^xiii^	57.50 (5)
Al12—Cr9—Al14	119.87 (4)	Cr5^vi^—Al32—Al3	52.55 (4)
Al34^vi^—Cr9—Al14	114.23 (5)	Cr7—Al32—Al3	101.95 (5)
Al20—Cr9—Al14	61.62 (5)	Al25^x^—Al32—Al3	89.53 (6)
Al23^ix^—Cr9—Al14	171.06 (5)	Al41^iv^—Al32—Al3	59.84 (5)
Al5—Cr9—Al14	59.69 (4)	Al38—Al32—Al3	110.11 (6)
Cr16—Cr9—Al14	59.69 (4)	Al40^xi^—Al32—Al3	147.39 (7)
Cr13—Cr9—Al14	62.71 (4)	Al20—Al32—Al3	112.30 (6)
Al38—Cr9—Al14	64.12 (4)	Al23^vi^—Al32—Al3	152.68 (7)
Al40—Cr9—Al14	117.26 (5)	Al19^vi^—Al32—Al3	57.00 (5)
Al12—Cr9—Al16	118.87 (4)	Al24^vi^—Al32—Al3	105.08 (6)
Al34^vi^—Cr9—Al16	113.14 (5)	Al6^xiii^—Al32—Al3	59.26 (5)
Al20—Cr9—Al16	171.80 (5)	Al15—Al33—Al15^iv^	57.00 (6)
Al23^ix^—Cr9—Al16	60.78 (5)	Cr19—Al33—Cr14	57.26 (4)
Al5—Cr9—Al16	59.26 (4)	Al15—Al33—Cr14	57.26 (4)
Cr16—Cr9—Al16	59.26 (4)	Al15^iv^—Al33—Cr14	107.26 (6)
Cr13—Cr9—Al16	62.71 (4)	Cr19—Al33—Al7	102.84 (6)
Al38—Cr9—Al16	117.04 (5)	Al15—Al33—Al7	102.84 (6)
Al40—Cr9—Al16	63.44 (4)	Al15^iv^—Al33—Al7	99.69 (6)
Al14—Cr9—Al16	112.03 (5)	Cr14—Al33—Al7	121.80 (6)
Al12—Cr9—Al18	124.93 (6)	Cr19—Al33—Al51	154.47 (8)
Al34^vi^—Cr9—Al18	62.90 (5)	Al15—Al33—Al51	154.47 (8)
Al20—Cr9—Al18	116.77 (5)	Al15^iv^—Al33—Al51	111.30 (6)
Al23^ix^—Cr9—Al18	64.66 (5)	Cr14—Al33—Al51	114.27 (6)
Al5—Cr9—Al18	57.83 (4)	Al7—Al33—Al51	101.55 (6)
Cr16—Cr9—Al18	57.83 (4)	Cr19—Al33—Cr4	55.56 (4)
Cr13—Cr9—Al18	109.02 (4)	Al15—Al33—Cr4	55.56 (4)
Al38—Cr9—Al18	169.48 (5)	Al15^iv^—Al33—Cr4	54.89 (4)
Al40—Cr9—Al18	116.32 (5)	Cr14—Al33—Cr4	105.52 (5)
Al14—Cr9—Al18	107.48 (5)	Al7—Al33—Cr4	53.36 (4)
Al16—Cr9—Al18	58.85 (4)	Al51—Al33—Cr4	140.19 (6)
Al3^iv^—Cr10—Al10^iv^	71.08 (5)	Al15—Al33—Al37^iv^	57.70 (5)
Al3^iv^—Cr10—Al15^iv^	115.27 (5)	Al15^iv^—Al33—Al37^iv^	105.20 (6)
Al10^iv^—Cr10—Al15^iv^	172.37 (6)	Cr14—Al33—Al37^iv^	59.24 (4)
Al3^iv^—Cr10—Al46	69.35 (5)	Al7—Al33—Al37^iv^	64.34 (5)
Al10^iv^—Cr10—Al46	69.18 (5)	Al51—Al33—Al37^iv^	142.75 (7)
Al15^iv^—Cr10—Al46	116.43 (5)	Cr4—Al33—Al37^iv^	60.38 (4)
Al3^iv^—Cr10—Cr17	172.78 (6)	Cr19—Al33—Al49	101.04 (6)
Al10^iv^—Cr10—Cr17	113.87 (5)	Al15—Al33—Al49	101.04 (6)
Al46—Cr10—Cr17	116.96 (5)	Al15^iv^—Al33—Al49	118.91 (7)
Al3^iv^—Cr10—Al11	172.78 (6)	Cr14—Al33—Al49	52.06 (4)
Al10^iv^—Cr10—Al11	113.87 (5)	Al7—Al33—Al49	141.25 (7)
Al15^iv^—Cr10—Al11	59.40 (5)	Al51—Al33—Al49	62.96 (5)
Al46—Cr10—Al11	116.96 (5)	Cr4—Al33—Al49	156.21 (6)
Al3^iv^—Cr10—Cr14^iv^	115.59 (4)	Al37^iv^—Al33—Al49	105.35 (6)
Al10^iv^—Cr10—Cr14^iv^	114.62 (4)	Cr19—Al33—Al61	100.75 (6)
Al15^iv^—Cr10—Cr14^iv^	59.38 (4)	Al15—Al33—Al61	100.75 (6)
Al46—Cr10—Cr14^iv^	174.29 (5)	Al15^iv^—Al33—Al61	157.70 (7)
Al11—Cr10—Cr14^iv^	57.92 (4)	Cr14—Al33—Al61	52.74 (4)
Al3^iv^—Cr10—Al41	67.39 (5)	Al7—Al33—Al61	86.16 (6)
Al10^iv^—Cr10—Al41	66.89 (5)	Al51—Al33—Al61	88.25 (6)
Al15^iv^—Cr10—Al41	110.86 (5)	Cr4—Al33—Al61	116.20 (6)
Al46—Cr10—Al41	125.68 (5)	Al37^iv^—Al33—Al61	57.88 (5)
Cr17—Cr10—Al41	109.10 (5)	Al49—Al33—Al61	59.58 (5)
Al11—Cr10—Al41	109.10 (5)	Cr19—Al33—Cr15	63.23 (5)
Cr14^iv^—Cr10—Al41	59.95 (4)	Cr14—Al33—Cr15	64.38 (4)
Al3^iv^—Cr10—Al64	122.67 (5)	Al7—Al33—Cr15	160.09 (7)
Al10^iv^—Cr10—Al64	118.41 (5)	Al51—Al33—Cr15	91.26 (6)
Al15^iv^—Cr10—Al64	62.56 (4)	Cr4—Al33—Cr15	107.40 (5)
Al46—Cr10—Al64	64.51 (5)	Al37^iv^—Al33—Cr15	113.18 (6)
Cr17—Cr10—Al64	60.45 (5)	Al49—Al33—Cr15	58.45 (5)
Al11—Cr10—Al64	60.45 (5)	Al61—Al33—Cr15	109.65 (6)
Cr14^iv^—Cr10—Al64	109.81 (4)	Al15—Al33—Al4	63.23 (5)
Al41—Cr10—Al64	169.24 (5)	Al15^iv^—Al33—Al4	61.13 (5)
Al3^iv^—Cr10—Al9	65.79 (5)	Cr14—Al33—Al4	64.38 (4)
Al10^iv^—Cr10—Al9	126.25 (5)	Al7—Al33—Al4	160.09 (7)
Al15^iv^—Cr10—Al9	61.35 (5)	Al51—Al33—Al4	91.26 (6)
Al46—Cr10—Al9	66.69 (5)	Cr4—Al33—Al4	107.40 (5)
Cr17—Cr10—Al9	112.60 (5)	Al37^iv^—Al33—Al4	113.18 (6)
Al11—Cr10—Al9	112.60 (5)	Al49—Al33—Al4	58.45 (5)
Cr14^iv^—Cr10—Al9	112.09 (4)	Al61—Al33—Al4	109.65 (6)
Al41—Cr10—Al9	119.63 (5)	Cr4—Al34—Cr9^vi^	163.14 (7)
Al64—Cr10—Al9	65.91 (5)	Cr4—Al34—Al23^iv^	117.52 (6)
Al3^iv^—Cr10—Al37	64.24 (5)	Cr9^vi^—Al34—Al23^iv^	57.09 (4)
Al10^iv^—Cr10—Al37	122.76 (5)	Cr4—Al34—Al37^iv^	64.79 (5)
Al15^iv^—Cr10—Al37	59.78 (4)	Cr9^vi^—Al34—Al37^iv^	128.66 (6)
Al46—Cr10—Al37	121.08 (5)	Al23^iv^—Al34—Al37^iv^	150.71 (7)
Cr17—Cr10—Al37	108.62 (5)	Cr4—Al34—Al20^vi^	116.90 (6)
Al11—Cr10—Al37	108.62 (5)	Cr9^vi^—Al34—Al20^vi^	56.59 (4)
Cr14^iv^—Cr10—Al37	61.10 (4)	Al23^iv^—Al34—Al20^vi^	107.28 (6)
Al41—Cr10—Al37	64.09 (4)	Al37^iv^—Al34—Al20^vi^	95.32 (6)
Al64—Cr10—Al37	115.21 (5)	Cr4—Al34—Al7	55.86 (4)
Al9—Cr10—Al37	62.41 (5)	Cr9^vi^—Al34—Al7	117.33 (6)
Al3^iv^—Cr10—Al22	127.57 (5)	Al23^iv^—Al34—Al7	143.51 (7)
Al10^iv^—Cr10—Al22	65.28 (5)	Al37^iv^—Al34—Al7	64.17 (5)
Al15^iv^—Cr10—Al22	111.05 (5)	Al20^vi^—Al34—Al7	61.52 (5)
Al46—Cr10—Al22	69.03 (5)	Cr4—Al34—Al9^iv^	66.02 (5)
Cr17—Cr10—Al22	59.53 (5)	Cr9^vi^—Al34—Al9^iv^	127.55 (6)
Al11—Cr10—Al22	59.53 (5)	Al23^iv^—Al34—Al9^iv^	92.29 (6)
Cr14^iv^—Cr10—Al22	108.22 (4)	Al37^iv^—Al34—Al9^iv^	60.99 (5)
Al41—Cr10—Al22	116.57 (5)	Al20^vi^—Al34—Al9^iv^	153.46 (7)
Al64—Cr10—Al22	61.67 (4)	Al7—Al34—Al9^iv^	111.89 (6)
Al9—Cr10—Al22	121.64 (5)	Cr4—Al34—Al19^vi^	128.03 (6)
Al37—Cr10—Al22	168.07 (5)	Cr9^vi^—Al34—Al19^vi^	65.23 (5)
Al6^iv^—Cr11—Al10^iii^	71.68 (5)	Al23^iv^—Al34—Al19^vi^	110.32 (6)
Al6^iv^—Cr11—Al13^iv^	115.00 (5)	Al37^iv^—Al34—Al19^vi^	63.63 (5)
Al10^iii^—Cr11—Al13^iv^	172.13 (6)	Al20^vi^—Al34—Al19^vi^	62.89 (5)
Al6^iv^—Cr11—Al47	69.65 (5)	Al7—Al34—Al19^vi^	95.41 (6)
Al10^iii^—Cr11—Al47	69.24 (5)	Al9^iv^—Al34—Al19^vi^	93.89 (6)
Al13^iv^—Cr11—Al47	116.36 (5)	Cr4—Al34—Al18^vi^	128.90 (6)
Al6^iv^—Cr11—Cr17	172.76 (6)	Cr9^vi^—Al34—Al18^vi^	65.05 (5)
Al10^iii^—Cr11—Cr17	113.24 (5)	Al23^iv^—Al34—Al18^vi^	62.56 (5)
Al47—Cr11—Cr17	116.71 (5)	Al37^iv^—Al34—Al18^vi^	92.61 (6)
Al6^iv^—Cr11—Al11	172.76 (6)	Al20^vi^—Al34—Al18^vi^	110.03 (6)
Al10^iii^—Cr11—Al11	113.24 (5)	Al7—Al34—Al18^vi^	152.75 (7)
Al13^iv^—Cr11—Al11	59.70 (5)	Al9^iv^—Al34—Al18^vi^	62.98 (5)
Al47—Cr11—Al11	116.71 (5)	Al19^vi^—Al34—Al18^vi^	59.82 (5)
Al6^iv^—Cr11—Cr12	115.72 (4)	Cr4—Al34—Al7^iv^	55.56 (4)
Al10^iii^—Cr11—Cr12	114.42 (4)	Cr9^vi^—Al34—Al7^iv^	118.37 (6)
Al13^iv^—Cr11—Cr12	59.51 (4)	Al23^iv^—Al34—Al7^iv^	62.33 (5)
Al47—Cr11—Cr12	174.00 (5)	Al37^iv^—Al34—Al7^iv^	109.42 (6)
Cr17—Cr11—Cr12	57.76 (4)	Al20^vi^—Al34—Al7^iv^	142.47 (7)
Al11—Cr11—Cr12	57.76 (4)	Al7—Al34—Al7^iv^	104.00 (6)
Al6^iv^—Cr11—Al25	67.65 (5)	Al9^iv^—Al34—Al7^iv^	62.73 (5)
Al10^iii^—Cr11—Al25	67.02 (5)	Al19^vi^—Al34—Al7^iv^	153.81 (7)
Al13^iv^—Cr11—Al25	110.87 (5)	Al18^vi^—Al34—Al7^iv^	96.92 (6)
Al47—Cr11—Al25	125.95 (5)	Cr4—Al34—Al12^vi^	110.46 (6)
Cr17—Cr11—Al25	108.79 (5)	Cr9^vi^—Al34—Al12^vi^	52.69 (4)
Al11—Cr11—Al25	108.79 (5)	Al23^iv^—Al34—Al12^vi^	59.18 (4)
Cr12—Cr11—Al25	59.89 (4)	Al37^iv^—Al34—Al12^vi^	149.89 (6)
Al6^iv^—Cr11—Al65^iv^	122.68 (5)	Al20^vi^—Al34—Al12^vi^	59.05 (4)
Al10^iii^—Cr11—Al65^iv^	118.02 (5)	Al7—Al34—Al12^vi^	88.16 (5)
Al13^iv^—Cr11—Al65^iv^	62.70 (5)	Al9^iv^—Al34—Al12^vi^	147.05 (6)
Al47—Cr11—Al65^iv^	64.29 (5)	Al19^vi^—Al34—Al12^vi^	110.57 (6)
Al11—Cr11—Al65^iv^	60.51 (5)	Al18^vi^—Al34—Al12^vi^	110.17 (6)
Cr12—Cr11—Al65^iv^	109.78 (4)	Al7^iv^—Al34—Al12^vi^	87.76 (5)
Al25—Cr11—Al65^iv^	169.06 (5)	Cr3—Al35—Cr8^xiv^	161.83 (7)
Al6^iv^—Cr11—Al44^iv^	65.49 (5)	Cr3—Al35—Al27^vii^	116.90 (6)
Al10^iii^—Cr11—Al44^iv^	126.60 (5)	Cr8^xiv^—Al35—Al27^vii^	57.54 (4)
Al13^iv^—Cr11—Al44^iv^	61.22 (5)	Cr3—Al35—Al30^xiv^	116.19 (6)
Al47—Cr11—Al44^iv^	67.03 (5)	Cr8^xiv^—Al35—Al30^xiv^	56.85 (4)
Al11—Cr11—Al44^iv^	112.92 (5)	Al27^vii^—Al35—Al30^xiv^	108.54 (6)
Cr12—Cr11—Al44^iv^	112.09 (4)	Cr3—Al35—Al26	129.35 (6)
Al25—Cr11—Al44^iv^	119.58 (5)	Cr8^xiv^—Al35—Al26	65.73 (5)
Al65^iv^—Cr11—Al44^iv^	66.30 (5)	Al27^vii^—Al35—Al26	62.33 (5)
Al6^iv^—Cr11—Al45	64.04 (5)	Al30^xiv^—Al35—Al26	110.31 (6)
Al10^iii^—Cr11—Al45	123.00 (5)	Cr3—Al35—Al45^iv^	64.14 (5)
Al13^iv^—Cr11—Al45	59.83 (4)	Cr8^xiv^—Al35—Al45^iv^	130.16 (6)
Al47—Cr11—Al45	121.36 (5)	Al27^vii^—Al35—Al45^iv^	150.80 (7)
Cr17—Cr11—Al45	108.81 (5)	Al30^xiv^—Al35—Al45^iv^	94.88 (6)
Al11—Cr11—Al45	108.81 (5)	Al26—Al35—Al45^iv^	93.69 (6)
Cr12—Cr11—Al45	61.29 (4)	Cr3—Al35—Al24^xiv^	128.28 (6)
Al25—Cr11—Al45	64.10 (4)	Cr8^xiv^—Al35—Al24^xiv^	65.89 (5)
Al65^iv^—Cr11—Al45	115.52 (5)	Al27^vii^—Al35—Al24^xiv^	110.78 (6)
Al44^iv^—Cr11—Al45	62.25 (5)	Al30^xiv^—Al35—Al24^xiv^	62.54 (5)
Al6^iv^—Cr11—Al21	127.74 (5)	Al26—Al35—Al24^xiv^	60.16 (5)
Al10^iii^—Cr11—Al21	64.80 (5)	Al45^iv^—Al35—Al24^xiv^	64.56 (5)
Al13^iv^—Cr11—Al21	111.19 (5)	Cr3—Al35—Al36^iv^	54.80 (4)
Al47—Cr11—Al21	68.91 (5)	Cr8^xiv^—Al35—Al36^iv^	117.55 (6)
Cr17—Cr11—Al21	59.37 (5)	Al27^vii^—Al35—Al36^iv^	143.28 (7)
Al11—Cr11—Al21	59.37 (5)	Al30^xiv^—Al35—Al36^iv^	61.81 (5)
Cr12—Cr11—Al21	107.88 (4)	Al26—Al35—Al36^iv^	153.57 (7)
Al25—Cr11—Al21	116.21 (5)	Al45^iv^—Al35—Al36^iv^	63.60 (5)
Al65^iv^—Cr11—Al21	61.66 (4)	Al24^xiv^—Al35—Al36^iv^	96.09 (6)
Al44^iv^—Cr11—Al21	122.10 (5)	Cr3—Al35—Al44	65.28 (5)
Al45—Cr11—Al21	168.05 (5)	Cr8^xiv^—Al35—Al44	129.37 (6)
Al52—Cr12—Al54	70.26 (5)	Al27^vii^—Al35—Al44	92.30 (6)
Al52—Cr12—Al62	69.99 (5)	Al30^xiv^—Al35—Al44	153.19 (7)
Al54—Cr12—Al62	71.54 (5)	Al26—Al35—Al44	64.19 (5)
Al52—Cr12—Cr17	115.45 (5)	Al45^iv^—Al35—Al44	60.70 (5)
Al54—Cr12—Cr17	114.67 (5)	Al24^xiv^—Al35—Al44	94.79 (6)
Al62—Cr12—Cr17	172.54 (6)	Al36^iv^—Al35—Al44	110.45 (6)
Al52—Cr12—Al11	115.45 (5)	Cr3—Al35—Al36	54.51 (4)
Al54—Cr12—Al11	114.67 (5)	Cr8^xiv^—Al35—Al36	118.77 (6)
Al62—Cr12—Al11	172.54 (6)	Al27^vii^—Al35—Al36	62.69 (5)
Al52—Cr12—Al13^iv^	115.28 (5)	Al30^xiv^—Al35—Al36	142.08 (7)
Al54—Cr12—Al13^iv^	173.10 (5)	Al26—Al35—Al36	97.94 (6)
Al62—Cr12—Al13^iv^	113.76 (5)	Al45^iv^—Al35—Al36	108.36 (6)
Al11—Cr12—Al13^iv^	59.72 (5)	Al24^xiv^—Al35—Al36	154.89 (7)
Al52—Cr12—Cr11	172.71 (5)	Al36^iv^—Al35—Al36	101.91 (6)
Al54—Cr12—Cr11	116.00 (5)	Al44—Al35—Al36	62.60 (5)
Al62—Cr12—Cr11	114.90 (5)	Cr3—Al35—Al1^vii^	109.04 (6)
Cr17—Cr12—Cr11	59.17 (4)	Cr8^xiv^—Al35—Al1^vii^	52.80 (4)
Al11—Cr12—Cr11	59.17 (4)	Al27^vii^—Al35—Al1^vii^	60.05 (4)
Al13^iv^—Cr12—Cr11	58.19 (4)	Al30^xiv^—Al35—Al1^vii^	59.80 (4)
Al52—Cr12—Al25	126.19 (5)	Al26—Al35—Al1^vii^	110.90 (6)
Al54—Cr12—Al25	66.63 (5)	Al45^iv^—Al35—Al1^vii^	149.12 (6)
Al62—Cr12—Al25	66.99 (5)	Al24^xiv^—Al35—Al1^vii^	111.23 (6)
Cr17—Cr12—Al25	110.93 (5)	Al36^iv^—Al35—Al1^vii^	87.51 (5)
Al11—Cr12—Al25	110.93 (5)	Al44—Al35—Al1^vii^	146.89 (6)
Al13^iv^—Cr12—Al25	110.72 (5)	Al36—Al35—Al1^vii^	87.11 (5)
Cr11—Cr12—Al25	61.03 (4)	Cr6—Al36—Cr3	166.10 (7)
Al52—Cr12—Al31	66.49 (5)	Cr6—Al36—Al28^i^	62.91 (5)
Al54—Cr12—Al31	127.11 (5)	Cr3—Al36—Al28^i^	130.95 (7)
Al62—Cr12—Al31	66.18 (5)	Cr6—Al36—Al31	129.03 (6)
Cr17—Cr12—Al31	110.56 (5)	Cr3—Al36—Al31	64.82 (5)
Al11—Cr12—Al31	110.56 (5)	Al28^i^—Al36—Al31	66.13 (5)
Al13^iv^—Cr12—Al31	59.78 (4)	Cr6—Al36—Al35^iv^	113.90 (6)
Cr11—Cr12—Al31	109.86 (4)	Cr3—Al36—Al35^iv^	56.50 (4)
Al25—Cr12—Al31	119.63 (5)	Al28^i^—Al36—Al35^iv^	143.98 (7)
Al52—Cr12—Al64	66.44 (5)	Al31—Al36—Al35^iv^	107.29 (6)
Al54—Cr12—Al64	66.44 (5)	Cr6—Al36—Al35	112.07 (6)
Al62—Cr12—Al64	126.97 (5)	Cr3—Al36—Al35	56.19 (4)
Cr17—Cr12—Al64	60.46 (5)	Al28^i^—Al36—Al35	148.52 (7)
Al11—Cr12—Al64	60.46 (5)	Al31—Al36—Al35	110.57 (6)
Al13^iv^—Cr12—Al64	111.29 (5)	Al35^iv^—Al36—Al35	67.48 (6)
Cr11—Cr12—Al64	111.84 (4)	Cr6—Al36—Al30^viii^	55.50 (4)
Al25—Cr12—Al64	119.63 (5)	Cr3—Al36—Al30^viii^	114.60 (6)
Al31—Cr12—Al64	118.41 (5)	Al28^i^—Al36—Al30^viii^	105.77 (6)
Al52—Cr12—Al45	119.75 (5)	Al31—Al36—Al30^viii^	145.72 (7)
Al54—Cr12—Al45	122.41 (5)	Al35^iv^—Al36—Al30^viii^	58.51 (5)
Al62—Cr12—Al45	62.89 (5)	Al35—Al36—Al30^viii^	93.15 (6)
Cr17—Cr12—Al45	109.66 (5)	Cr6—Al36—Al27^vii^	54.80 (4)
Al11—Cr12—Al45	109.66 (5)	Cr3—Al36—Al27^vii^	113.20 (6)
Al13^iv^—Cr12—Al45	59.31 (4)	Al28^i^—Al36—Al27^vii^	108.91 (6)
Cr11—Cr12—Al45	60.93 (4)	Al31—Al36—Al27^vii^	150.28 (7)
Al25—Cr12—Al45	64.36 (5)	Al35^iv^—Al36—Al27^vii^	93.15 (6)
Al31—Cr12—Al45	61.49 (5)	Al35—Al36—Al27^vii^	57.29 (5)
Al64—Cr12—Al45	169.91 (5)	Al30^viii^—Al36—Al27^vii^	63.71 (5)
Al52—Cr12—Al22	119.51 (5)	Cr6—Al36—Al17	61.40 (5)
Al54—Cr12—Al22	62.67 (5)	Cr3—Al36—Al17	120.75 (6)
Al62—Cr12—Al22	122.74 (5)	Al28^i^—Al36—Al17	63.85 (5)
Cr17—Cr12—Al22	59.99 (5)	Al31—Al36—Al17	95.52 (6)
Al11—Cr12—Al22	59.99 (5)	Al35^iv^—Al36—Al17	149.65 (7)
Al13^iv^—Cr12—Al22	110.45 (5)	Al35—Al36—Al17	86.10 (6)
Cr11—Cr12—Al22	63.18 (4)	Al30^viii^—Al36—Al17	110.95 (6)
Al25—Cr12—Al22	64.50 (5)	Al27^vii^—Al36—Al17	58.81 (5)
Al31—Cr12—Al22	169.97 (5)	Cr6—Al36—Al44	118.24 (6)
Al64—Cr12—Al22	61.30 (4)	Cr3—Al36—Al44	63.95 (4)
Al45—Cr12—Al22	116.83 (5)	Al28^i^—Al36—Al44	95.14 (6)
Al48—Cr13—Al53	72.78 (5)	Al31—Al36—Al44	65.22 (5)
Al48—Cr13—Al61	68.37 (5)	Al35^iv^—Al36—Al44	114.69 (6)
Al53—Cr13—Al61	69.29 (5)	Al35—Al36—Al44	58.44 (5)
Al48—Cr13—Al62^i^	68.35 (5)	Al30^viii^—Al36—Al44	148.00 (7)
Al53—Cr13—Al62^i^	69.23 (5)	Al27^vii^—Al36—Al44	86.88 (6)
Al61—Cr13—Al62^i^	126.61 (5)	Al17—Al36—Al44	57.27 (5)
Al48—Cr13—Cr16	113.26 (5)	Cr6—Al36—Al16^i^	61.61 (5)
Al53—Cr13—Cr16	173.94 (6)	Cr3—Al36—Al16^i^	123.92 (6)
Al61—Cr13—Cr16	112.76 (5)	Al28^i^—Al36—Al16^i^	58.14 (5)
Al62^i^—Cr13—Cr16	112.07 (5)	Al31—Al36—Al16^i^	92.20 (6)
Al48—Cr13—Al5	113.26 (5)	Al35^iv^—Al36—Al16^i^	87.95 (6)
Al53—Cr13—Al5	173.94 (6)	Al35—Al36—Al16^i^	150.20 (7)
Al61—Cr13—Al5	112.76 (5)	Al30^viii^—Al36—Al16^i^	58.56 (5)
Al62^i^—Cr13—Al5	112.07 (5)	Al27^vii^—Al36—Al16^i^	110.28 (6)
Al48—Cr13—Cr9	172.85 (5)	Al17—Al36—Al16^i^	111.37 (6)
Al53—Cr13—Cr9	114.37 (5)	Al44—Al36—Al16^i^	151.33 (7)
Al61—Cr13—Cr9	113.43 (4)	Cr6—Al36—Al45	123.43 (6)
Al62^i^—Cr13—Cr9	113.46 (4)	Cr3—Al36—Al45	62.20 (4)
Cr16—Cr13—Cr9	59.59 (4)	Al28^i^—Al36—Al45	92.52 (6)
Al5—Cr13—Cr9	59.59 (4)	Al31—Al36—Al45	58.87 (5)
Al48—Cr13—Al40	124.42 (5)	Al35^iv^—Al36—Al45	57.97 (5)
Al53—Cr13—Al40	64.62 (5)	Al35—Al36—Al45	112.92 (6)
Al61—Cr13—Al40	122.06 (5)	Al30^viii^—Al36—Al45	89.61 (6)
Al62^i^—Cr13—Al40	64.30 (5)	Al27^vii^—Al36—Al45	149.10 (7)
Cr16—Cr13—Al40	110.27 (5)	Al17—Al36—Al45	151.66 (7)
Al5—Cr13—Al40	110.27 (5)	Al44—Al36—Al45	113.79 (6)
Cr9—Cr13—Al40	61.11 (4)	Al16^i^—Al36—Al45	62.20 (5)
Al48—Cr13—Al38	124.60 (5)	Al15^iv^—Al37—Cr10	56.71 (4)
Al53—Cr13—Al38	64.52 (5)	Al15^iv^—Al37—Cr14^iv^	57.31 (4)
Al61—Cr13—Al38	64.63 (5)	Cr10—Al37—Cr14^iv^	58.02 (4)
Al62^i^—Cr13—Al38	121.72 (5)	Al15^iv^—Al37—Al61^iv^	104.17 (6)
Cr16—Cr13—Al38	110.78 (5)	Cr10—Al37—Al61^iv^	104.74 (6)
Al5—Cr13—Al38	110.78 (5)	Cr14^iv^—Al37—Al61^iv^	53.91 (4)
Cr9—Cr13—Al38	60.96 (4)	Al15^iv^—Al37—Al34^iv^	100.10 (6)
Al40—Cr13—Al38	64.94 (5)	Cr10—Al37—Al34^iv^	117.61 (6)
Al48—Cr13—Al14	115.12 (5)	Cr14^iv^—Al37—Al34^iv^	156.47 (7)
Al53—Cr13—Al14	119.02 (5)	Al61^iv^—Al37—Al34^iv^	137.63 (7)
Al61—Cr13—Al14	61.12 (5)	Al15^iv^—Al37—Al3^iv^	102.89 (6)
Al62^i^—Cr13—Al14	171.45 (5)	Cr10—Al37—Al3^iv^	52.97 (4)
Cr16—Cr13—Al14	59.48 (4)	Cr14^iv^—Al37—Al3^iv^	103.44 (6)
Al5—Cr13—Al14	59.48 (4)	Al61^iv^—Al37—Al3^iv^	119.81 (7)
Cr9—Cr13—Al14	62.09 (4)	Al34^iv^—Al37—Al3^iv^	87.08 (6)
Al40—Cr13—Al14	116.05 (5)	Al15^iv^—Al37—Al33^iv^	58.17 (5)
Al38—Cr13—Al14	63.56 (4)	Cr10—Al37—Al33^iv^	105.58 (6)
Al48—Cr13—Al29	63.58 (5)	Cr14^iv^—Al37—Al33^iv^	58.95 (4)
Al53—Cr13—Al29	124.70 (5)	Al61^iv^—Al37—Al33^iv^	61.93 (5)
Al61—Cr13—Al29	64.24 (5)	Al34^iv^—Al37—Al33^iv^	105.20 (6)
Al62^i^—Cr13—Al29	118.87 (5)	Al3^iv^—Al37—Al33^iv^	158.53 (7)
Cr16—Cr13—Al29	60.33 (5)	Al15^iv^—Al37—Cr4	55.38 (4)
Al5—Cr13—Al29	60.33 (5)	Cr10—Al37—Cr4	104.79 (5)
Cr9—Cr13—Al29	110.49 (4)	Cr14^iv^—Al37—Cr4	104.61 (5)
Al40—Cr13—Al29	170.53 (5)	Al61^iv^—Al37—Cr4	119.23 (6)
Al38—Cr13—Al29	115.99 (5)	Al34^iv^—Al37—Cr4	52.50 (4)
Al14—Cr13—Al29	59.34 (4)	Al3^iv^—Al37—Cr4	120.49 (6)
Al48—Cr13—Al28	63.67 (5)	Al33^iv^—Al37—Cr4	59.47 (4)
Al53—Cr13—Al28	124.53 (5)	Al15^iv^—Al37—Al9	58.98 (5)
Al61—Cr13—Al28	119.15 (5)	Cr10—Al37—Al9	58.63 (4)
Al62^i^—Cr13—Al28	63.95 (5)	Cr14^iv^—Al37—Al9	106.51 (6)
Cr16—Cr13—Al28	60.02 (4)	Al61^iv^—Al37—Al9	160.42 (7)
Al5—Cr13—Al28	60.02 (4)	Al34^iv^—Al37—Al9	60.21 (5)
Cr9—Cr13—Al28	110.43 (4)	Al3^iv^—Al37—Al9	60.54 (5)
Al40—Cr13—Al28	115.27 (5)	Al33^iv^—Al37—Al9	110.05 (6)
Al38—Cr13—Al28	170.64 (5)	Cr4—Al37—Al9	61.55 (4)
Al14—Cr13—Al28	109.90 (5)	Al15^iv^—Al37—Al41	103.22 (6)
Al29—Cr13—Al28	62.12 (4)	Cr10—Al37—Al41	57.58 (4)
Al49—Cr14—Al57^i^	70.57 (5)	Cr14^iv^—Al37—Al41	56.82 (4)
Al49—Cr14—Al61	69.98 (5)	Al61^iv^—Al37—Al41	60.94 (5)
Al57^i^—Cr14—Al61	71.63 (5)	Al34^iv^—Al37—Al41	143.79 (7)
Al49—Cr14—Al11^iv^	115.29 (6)	Al3^iv^—Al37—Al41	61.00 (5)
Al57^i^—Cr14—Al11^iv^	114.72 (5)	Al33^iv^—Al37—Al41	110.57 (6)
Al61—Cr14—Al11^iv^	172.52 (6)	Cr4—Al37—Al41	158.57 (7)
Al49—Cr14—Cr19	114.87 (5)	Al9—Al37—Al41	110.54 (6)
Al57^i^—Cr14—Cr19	173.02 (5)	Al15^iv^—Al37—Al19^xv^	149.60 (7)
Al61—Cr14—Cr19	113.92 (5)	Cr10—Al37—Al19^xv^	110.81 (6)
Al49—Cr14—Al15	114.87 (5)	Cr14^iv^—Al37—Al19^xv^	144.31 (6)
Al57^i^—Cr14—Al15	173.02 (5)	Al61^iv^—Al37—Al19^xv^	105.96 (6)
Al61—Cr14—Al15	113.92 (5)	Al34^iv^—Al37—Al19^xv^	58.85 (5)
Al11^iv^—Cr14—Al15	59.45 (5)	Al3^iv^—Al37—Al19^xv^	57.89 (5)
Al49—Cr14—Cr10^iv^	172.46 (5)	Al33^iv^—Al37—Al19^xv^	143.57 (7)
Al57^i^—Cr14—Cr10^iv^	115.90 (5)	Cr4—Al37—Al19^xv^	111.08 (6)
Al61—Cr14—Cr10^iv^	115.08 (5)	Al9—Al37—Al19^xv^	90.66 (6)
Al11^iv^—Cr14—Cr10^iv^	59.13 (4)	Al41—Al37—Al19^xv^	88.06 (6)
Al15—Cr14—Cr10^iv^	58.37 (4)	Al15^iv^—Al37—Al7^iv^	97.59 (6)
Al49—Cr14—Al41^iv^	126.39 (5)	Cr10—Al37—Al7^iv^	153.98 (6)
Al57^i^—Cr14—Al41^iv^	66.60 (5)	Cr14^iv^—Al37—Al7^iv^	114.05 (6)
Al61—Cr14—Al41^iv^	67.07 (5)	Al61^iv^—Al37—Al7^iv^	84.19 (6)
Al11^iv^—Cr14—Al41^iv^	111.06 (5)	Al34^iv^—Al37—Al7^iv^	58.38 (5)
Al15—Cr14—Al41^iv^	110.97 (5)	Al3^iv^—Al37—Al7^iv^	142.51 (7)
Cr10^iv^—Cr14—Al41^iv^	61.08 (4)	Al33^iv^—Al37—Al7^iv^	56.62 (5)
Al49—Cr14—Al33	66.06 (5)	Cr4—Al37—Al7^iv^	50.89 (4)
Al57^i^—Cr14—Al33	127.13 (5)	Al9—Al37—Al7^iv^	106.72 (6)
Al61—Cr14—Al33	66.24 (5)	Al41—Al37—Al7^iv^	142.67 (6)
Al11^iv^—Cr14—Al33	110.32 (5)	Al19^xv^—Al37—Al7^iv^	89.36 (6)
Cr19—Cr14—Al33	59.85 (4)	Cr9—Al38—Cr13	57.01 (4)
Al15—Cr14—Al33	59.85 (4)	Cr9—Al38—Al53	101.61 (6)
Cr10^iv^—Cr14—Al33	110.14 (4)	Cr13—Al38—Al53	53.10 (4)
Al41^iv^—Cr14—Al33	119.90 (5)	Cr9—Al38—Al12	52.69 (4)
Al49—Cr14—Al37^iv^	119.79 (5)	Cr13—Al38—Al12	101.57 (5)
Al57^i^—Cr14—Al37^iv^	122.58 (5)	Al53—Al38—Al12	114.81 (6)
Al61—Cr14—Al37^iv^	63.11 (5)	Cr9—Al38—Al61	101.91 (6)
Al11^iv^—Cr14—Al37^iv^	109.43 (5)	Cr13—Al38—Al61	54.43 (4)
Al15—Cr14—Al37^iv^	59.40 (4)	Al53—Al38—Al61	61.03 (5)
Cr10^iv^—Cr14—Al37^iv^	60.88 (4)	Al12—Al38—Al61	154.20 (6)
Al41^iv^—Cr14—Al37^iv^	64.41 (5)	Cr9—Al38—Al20	53.97 (4)
Al33—Cr14—Al37^iv^	61.82 (4)	Cr13—Al38—Al20	101.57 (6)
Al49—Cr14—Al65	66.57 (5)	Al53—Al38—Al20	154.07 (7)
Al57^i^—Cr14—Al65	66.65 (5)	Al12—Al38—Al20	60.04 (5)
Al61—Cr14—Al65	127.13 (5)	Al61—Al38—Al20	111.38 (6)
Al11^iv^—Cr14—Al65	60.29 (5)	Cr9—Al38—Al32	113.95 (6)
Cr19—Cr14—Al65	110.80 (5)	Cr13—Al38—Al32	151.56 (7)
Al15—Cr14—Al65	110.80 (5)	Al53—Al38—Al32	144.44 (7)
Cr10^iv^—Cr14—Al65	111.62 (4)	Al12—Al38—Al32	88.59 (5)
Al41^iv^—Cr14—Al65	119.79 (5)	Al61—Al38—Al32	108.73 (6)
Al33—Cr14—Al65	118.02 (5)	Al20—Al38—Al32	60.58 (5)
Al37^iv^—Cr14—Al65	169.50 (5)	Cr9—Al38—Al59	146.72 (6)
Al49—Cr14—Al21^iv^	119.88 (5)	Cr13—Al38—Al59	110.89 (6)
Al57^i^—Cr14—Al21^iv^	62.77 (5)	Al53—Al38—Al59	57.86 (5)
Al61—Cr14—Al21^iv^	122.88 (5)	Al12—Al38—Al59	108.81 (6)
Al11^iv^—Cr14—Al21^iv^	60.04 (5)	Al61—Al38—Al59	90.59 (6)
Al15—Cr14—Al21^iv^	110.25 (5)	Al20—Al38—Al59	147.40 (7)
Cr10^iv^—Cr14—Al21^iv^	62.89 (4)	Al32—Al38—Al59	90.22 (6)
Al41^iv^—Cr14—Al21^iv^	64.45 (5)	Cr9—Al38—Al40^xi^	111.28 (6)
Al33—Cr14—Al21^iv^	169.81 (5)	Cr13—Al38—Al40^xi^	147.31 (6)
Al37^iv^—Cr14—Al21^iv^	116.59 (5)	Al53—Al38—Al40^xi^	108.45 (6)
Al65—Cr14—Al21^iv^	61.48 (4)	Al12—Al38—Al40^xi^	58.65 (4)
Cr8^x^—Al1—Cr8^i^	177.52 (11)	Al61—Al38—Al40^xi^	146.73 (7)
Cr8^x^—Al1—Al42^i^	121.65 (7)	Al20—Al38—Al40^xi^	90.59 (6)
Cr8^i^—Al1—Al42^i^	60.25 (4)	Al32—Al38—Al40^xi^	59.82 (5)
Cr8^x^—Al1—Al42^x^	60.25 (4)	Al59—Al38—Al40^xi^	60.25 (5)
Cr8^i^—Al1—Al42^x^	121.65 (6)	Cr9—Al38—Al41^iv^	151.19 (6)
Al42^i^—Al1—Al42^x^	97.63 (9)	Cr13—Al38—Al41^iv^	113.95 (6)
Cr8^x^—Al1—Al43^x^	59.94 (4)	Al53—Al38—Al41^iv^	89.18 (6)
Cr8^i^—Al1—Al43^x^	121.97 (6)	Al12—Al38—Al41^iv^	144.44 (6)
Al42^i^—Al1—Al43^x^	61.72 (5)	Al61—Al38—Al41^iv^	60.08 (5)
Al42^x^—Al1—Al43^x^	66.73 (6)	Al20—Al38—Al41^iv^	108.81 (6)
Cr8^x^—Al1—Al43^i^	121.97 (6)	Al32—Al38—Al41^iv^	59.26 (5)
Cr8^i^—Al1—Al43^i^	59.94 (4)	Al59—Al38—Al41^iv^	60.78 (5)
Al42^i^—Al1—Al43^i^	66.73 (6)	Al40^xi^—Al38—Al41^iv^	89.86 (6)
Al42^x^—Al1—Al43^i^	61.72 (5)	Cr9—Al38—Al14	59.40 (4)
Al43^x^—Al1—Al43^i^	97.43 (9)	Cr13—Al38—Al14	59.49 (4)
Cr8^x^—Al1—Al27^xi^	57.35 (3)	Al53—Al38—Al14	105.77 (6)
Cr8^i^—Al1—Al27^xi^	121.77 (4)	Al12—Al38—Al14	105.00 (6)
Al42^i^—Al1—Al27^xi^	155.18 (8)	Al61—Al38—Al14	56.74 (5)
Al42^x^—Al1—Al27^xi^	59.26 (4)	Al20—Al38—Al14	56.55 (5)
Al43^x^—Al1—Al27^xi^	110.88 (5)	Al32—Al38—Al14	92.32 (6)
Al43^i^—Al1—Al27^xi^	92.34 (4)	Al59—Al38—Al14	146.15 (7)
Cr8^x^—Al1—Al27	121.77 (4)	Al40^xi^—Al38—Al14	145.74 (7)
Cr8^i^—Al1—Al27	57.35 (3)	Al41^iv^—Al38—Al14	92.05 (6)
Al42^i^—Al1—Al27	59.26 (4)	Cr9—Al38—Al40	57.27 (4)
Al42^x^—Al1—Al27	155.18 (8)	Cr13—Al38—Al40	57.47 (4)
Al43^x^—Al1—Al27	92.34 (4)	Al53—Al38—Al40	58.26 (5)
Al43^i^—Al1—Al27	110.88 (5)	Al12—Al38—Al40	58.33 (4)
Al27^xi^—Al1—Al27	144.96 (10)	Al61—Al38—Al40	106.50 (6)
Cr8^x^—Al1—Al30^i^	122.15 (4)	Al20—Al38—Al40	105.74 (6)
Cr8^i^—Al1—Al30^i^	56.97 (3)	Al32—Al38—Al40	144.77 (7)
Al42^i^—Al1—Al30^i^	110.94 (5)	Al59—Al38—Al40	89.69 (6)
Al42^x^—Al1—Al30^i^	92.11 (4)	Al40^xi^—Al38—Al40	90.12 (6)
Al43^x^—Al1—Al30^i^	154.94 (8)	Al41^iv^—Al38—Al40	145.45 (7)
Al43^i^—Al1—Al30^i^	59.28 (4)	Al14—Al38—Al40	106.81 (6)
Al27^xi^—Al1—Al30^i^	64.82 (4)	Cr5^xiv^—Al39—Cr6^vii^	59.29 (4)
Al27—Al1—Al30^i^	104.19 (5)	Cr5^xiv^—Al39—Al43^x^	149.27 (7)
Cr8^x^—Al1—Al30^x^	56.97 (3)	Cr6^vii^—Al39—Al43^x^	112.25 (6)
Cr8^i^—Al1—Al30^x^	122.15 (4)	Cr5^xiv^—Al39—Al42^i^	149.32 (7)
Al42^i^—Al1—Al30^x^	92.11 (4)	Cr6^vii^—Al39—Al42^i^	111.67 (6)
Al42^x^—Al1—Al30^x^	110.94 (5)	Al43^x^—Al39—Al42^i^	60.65 (5)
Al43^x^—Al1—Al30^x^	59.28 (4)	Cr5^xiv^—Al39—Al30^x^	102.39 (6)
Al43^i^—Al1—Al30^x^	154.94 (8)	Cr6^vii^—Al39—Al30^x^	53.91 (4)
Al27^xi^—Al1—Al30^x^	104.19 (5)	Al43^x^—Al39—Al30^x^	58.95 (5)
Al27—Al1—Al30^x^	64.82 (4)	Al42^i^—Al39—Al30^x^	91.28 (6)
Al30^i^—Al1—Al30^x^	145.16 (10)	Cr5^xiv^—Al39—Al27	102.54 (6)
Cr8^x^—Al1—Al35^xii^	56.34 (4)	Cr6^vii^—Al39—Al27	53.58 (4)
Cr8^i^—Al1—Al35^xii^	121.18 (8)	Al43^x^—Al39—Al27	91.39 (6)
Al42^i^—Al1—Al35^xii^	146.42 (5)	Al42^i^—Al39—Al27	58.64 (5)
Al42^x^—Al1—Al35^xii^	106.18 (4)	Al30^x^—Al39—Al27	64.85 (5)
Al43^x^—Al1—Al35^xii^	106.51 (4)	Cr5^xiv^—Al39—Al47^iv^	112.07 (6)
Al43^i^—Al1—Al35^xii^	146.07 (5)	Cr6^vii^—Al39—Al47^iv^	150.19 (7)
Al27^xi^—Al1—Al35^xii^	56.98 (5)	Al43^x^—Al39—Al47^iv^	88.89 (6)
Al27—Al1—Al35^xii^	92.11 (6)	Al42^i^—Al39—Al47^iv^	59.57 (5)
Al30^i^—Al1—Al35^xii^	91.78 (6)	Al30^x^—Al39—Al47^iv^	145.50 (7)
Al30^x^—Al1—Al35^xii^	57.55 (5)	Al27—Al39—Al47^iv^	107.59 (6)
Cr8^x^—Al1—Al35^vii^	121.18 (8)	Cr5^xiv^—Al39—Al46^x^	112.08 (6)
Cr8^i^—Al1—Al35^vii^	56.34 (4)	Cr6^vii^—Al39—Al46^x^	151.02 (6)
Al42^i^—Al1—Al35^vii^	106.18 (4)	Al43^x^—Al39—Al46^x^	59.40 (5)
Al42^x^—Al1—Al35^vii^	146.42 (5)	Al42^i^—Al39—Al46^x^	88.96 (6)
Al43^x^—Al1—Al35^vii^	146.07 (5)	Al30^x^—Al39—Al46^x^	107.81 (6)
Al43^i^—Al1—Al35^vii^	106.51 (4)	Al27—Al39—Al46^x^	145.33 (7)
Al27^xi^—Al1—Al35^vii^	92.10 (6)	Al47^iv^—Al39—Al46^x^	57.92 (5)
Al27—Al1—Al35^vii^	56.98 (5)	Cr5^xiv^—Al39—Al18^xiv^	59.37 (4)
Al30^i^—Al1—Al35^vii^	57.55 (5)	Cr6^vii^—Al39—Al18^xiv^	60.65 (4)
Al30^x^—Al1—Al35^vii^	91.78 (6)	Al43^x^—Al39—Al18^xiv^	90.31 (6)
Al35^xii^—Al1—Al35^vii^	64.84 (7)	Al42^i^—Al39—Al18^xiv^	145.94 (7)
Cr8—Al2—Cr7^vi^	119.49 (6)	Al30^x^—Al39—Al18^xiv^	56.55 (5)
Cr8—Al2—Cr5	121.40 (6)	Al27—Al39—Al18^xiv^	108.98 (6)
Cr7^vi^—Al2—Cr5	60.33 (4)	Al47^iv^—Al39—Al18^xiv^	143.43 (7)
Cr8—Al2—Cr6^i^	120.06 (6)	Al46^x^—Al39—Al18^xiv^	90.77 (6)
Cr7^vi^—Al2—Cr6^i^	111.17 (5)	Cr5^xiv^—Al39—Al3^xiii^	52.90 (4)
Cr5—Al2—Cr6^i^	61.11 (4)	Cr6^vii^—Al39—Al3^xiii^	103.39 (5)
Cr8—Al2—Al5	179.68 (8)	Al43^x^—Al39—Al3^xiii^	108.27 (6)
Cr7^vi^—Al2—Al5	60.28 (5)	Al42^i^—Al39—Al3^xiii^	144.85 (7)
Cr5—Al2—Al5	58.74 (5)	Al30^x^—Al39—Al3^xiii^	111.88 (6)
Cr6^i^—Al2—Al5	60.26 (5)	Al27—Al39—Al3^xiii^	155.01 (7)
Cr8—Al2—Cr16	179.68 (8)	Al47^iv^—Al39—Al3^xiii^	88.56 (6)
Cr7^vi^—Al2—Cr16	60.28 (5)	Al46^x^—Al39—Al3^xiii^	59.55 (5)
Cr5—Al2—Cr16	58.74 (5)	Al18^xiv^—Al39—Al3^xiii^	57.18 (5)
Cr6^i^—Al2—Cr16	60.26 (5)	Cr5^xiv^—Al39—Al6	52.68 (4)
Cr8—Al2—Al26^v^	68.23 (5)	Cr6^vii^—Al39—Al6	102.65 (5)
Cr7^vi^—Al2—Al26^v^	114.25 (6)	Al43^x^—Al39—Al6	145.05 (7)
Cr5—Al2—Al26^v^	62.93 (5)	Al42^i^—Al39—Al6	108.50 (6)
Cr6^i^—Al2—Al26^v^	63.07 (5)	Al30^x^—Al39—Al6	154.56 (7)
Al5—Al2—Al26^v^	112.05 (7)	Al27—Al39—Al6	111.58 (6)
Cr8—Al2—Al24	68.11 (5)	Al47^iv^—Al39—Al6	59.80 (5)
Cr7^vi^—Al2—Al24	62.69 (5)	Al46^x^—Al39—Al6	88.89 (6)
Cr5—Al2—Al24	62.43 (5)	Al18^xiv^—Al39—Al6	105.55 (6)
Cr6^i^—Al2—Al24	114.46 (6)	Al3^xiii^—Al39—Al6	59.79 (5)
Al5—Al2—Al24	111.84 (7)	Cr5^xiv^—Al39—Al26	58.83 (4)
Cr16—Al2—Al24	111.84 (7)	Cr6^vii^—Al39—Al26	58.76 (4)
Al26^v^—Al2—Al24	63.51 (5)	Al43^x^—Al39—Al26	146.15 (7)
Cr8—Al2—Al63	60.69 (5)	Al42^i^—Al39—Al26	90.96 (6)
Cr7^vi^—Al2—Al63	118.68 (6)	Al30^x^—Al39—Al26	107.82 (6)
Cr5—Al2—Al63	177.88 (7)	Al27—Al39—Al26	56.23 (5)
Cr6^i^—Al2—Al63	118.48 (6)	Al47^iv^—Al39—Al26	91.89 (6)
Al5—Al2—Al63	119.17 (7)	Al46^x^—Al39—Al26	144.37 (7)
Cr16—Al2—Al63	119.17 (7)	Al18^xiv^—Al39—Al26	108.29 (6)
Al26^v^—Al2—Al63	118.94 (7)	Al3^xiii^—Al39—Al26	105.58 (6)
Al24—Al2—Al63	119.06 (7)	Al6—Al39—Al26	57.51 (5)
Cr8—Al2—Al17^i^	65.98 (5)	Cr9—Al40—Cr13	57.01 (4)
Cr7^vi^—Al2—Al17^i^	173.66 (7)	Cr9—Al40—Al53	101.48 (5)
Cr5—Al2—Al17^i^	114.56 (6)	Cr13—Al40—Al53	53.12 (4)
Cr6^i^—Al2—Al17^i^	62.49 (5)	Cr9—Al40—Al12	52.68 (4)
Al5—Al2—Al17^i^	114.26 (7)	Cr13—Al40—Al12	101.70 (5)
Al26^v^—Al2—Al17^i^	63.84 (5)	Al53—Al40—Al12	114.82 (6)
Al24—Al2—Al17^i^	119.26 (7)	Cr9—Al40—Al62^i^	102.40 (6)
Al63—Al2—Al17^i^	66.29 (5)	Cr13—Al40—Al62^i^	54.84 (4)
Cr8—Al2—Al8	65.01 (5)	Al53—Al40—Al62^i^	61.24 (5)
Cr7^vi^—Al2—Al8	63.10 (5)	Al12—Al40—Al62^i^	154.72 (6)
Cr5—Al2—Al8	114.41 (6)	Cr9—Al40—Al23^ix^	54.28 (4)
Cr6^i^—Al2—Al8	174.26 (7)	Cr13—Al40—Al23^ix^	101.70 (6)
Al5—Al2—Al8	114.68 (7)	Al53—Al40—Al23^ix^	154.23 (7)
Cr16—Al2—Al8	114.68 (7)	Al12—Al40—Al23^ix^	60.12 (5)
Al26^v^—Al2—Al8	118.96 (7)	Al62^i^—Al40—Al23^ix^	111.41 (6)
Al24—Al2—Al8	64.02 (5)	Cr9—Al40—Al32^xi^	114.46 (6)
Al63—Al2—Al8	65.85 (5)	Cr13—Al40—Al32^xi^	151.96 (6)
Al17^i^—Al2—Al8	123.23 (7)	Al53—Al40—Al32^xi^	144.06 (7)
Cr8—Al2—Al28	120.19 (6)	Al12—Al40—Al32^xi^	88.56 (5)
Cr7^vi^—Al2—Al28	111.69 (6)	Al62^i^—Al40—Al32^xi^	108.45 (6)
Cr5—Al2—Al28	109.39 (6)	Al23^ix^—Al40—Al32^xi^	60.85 (5)
Cr6^i^—Al2—Al28	60.40 (4)	Cr9—Al40—Al38^xi^	111.34 (6)
Al5—Al2—Al28	59.87 (5)	Cr13—Al40—Al38^xi^	147.16 (6)
Cr16—Al2—Al28	59.87 (5)	Al53—Al40—Al38^xi^	108.08 (6)
Al26^v^—Al2—Al28	116.18 (6)	Al12—Al40—Al38^xi^	58.70 (4)
Al24—Al2—Al28	171.42 (7)	Al62^i^—Al40—Al38^xi^	146.17 (7)
Al63—Al2—Al28	69.04 (5)	Al23^ix^—Al40—Al38^xi^	90.87 (6)
Al17^i^—Al2—Al28	65.59 (5)	Al32^xi^—Al40—Al38^xi^	59.71 (5)
Al8—Al2—Al28	120.33 (7)	Cr9—Al40—Al16	60.23 (4)
Cr10^iv^—Al3—Cr5^vi^	168.96 (7)	Cr13—Al40—Al16	60.06 (4)
Cr10^iv^—Al3—Al18^vi^	125.02 (7)	Al53—Al40—Al16	106.14 (6)
Cr5^vi^—Al3—Al18^vi^	62.96 (5)	Al12—Al40—Al16	105.44 (6)
Cr10^iv^—Al3—Al19^vi^	126.85 (6)	Al62^i^—Al40—Al16	56.75 (5)
Cr5^vi^—Al3—Al19^vi^	62.76 (5)	Al23^ix^—Al40—Al16	56.50 (5)
Al18^vi^—Al3—Al19^vi^	60.95 (5)	Al32^xi^—Al40—Al16	92.15 (6)
Cr10^iv^—Al3—Al37^iv^	62.80 (5)	Al38^xi^—Al40—Al16	145.77 (7)
Cr5^vi^—Al3—Al37^iv^	126.79 (7)	Cr9—Al40—Al59^xi^	146.22 (6)
Al18^vi^—Al3—Al37^iv^	93.74 (6)	Cr13—Al40—Al59^xi^	110.80 (6)
Al19^vi^—Al3—Al37^iv^	64.13 (5)	Al53—Al40—Al59^xi^	57.74 (5)
Cr10^iv^—Al3—Al9^iv^	61.72 (5)	Al12—Al40—Al59^xi^	108.54 (6)
Cr5^vi^—Al3—Al9^iv^	126.25 (7)	Al62^i^—Al40—Al59^xi^	90.52 (6)
Al18^vi^—Al3—Al9^iv^	63.45 (5)	Al23^ix^—Al40—Al59^xi^	147.35 (7)
Al19^vi^—Al3—Al9^iv^	94.45 (6)	Al32^xi^—Al40—Al59^xi^	89.93 (6)
Al37^iv^—Al3—Al9^iv^	60.77 (5)	Al38^xi^—Al40—Al59^xi^	59.86 (5)
Cr10^iv^—Al3—Al46^iv^	56.97 (4)	Al16—Al40—Al59^xi^	146.01 (7)
Cr5^vi^—Al3—Al46^iv^	117.93 (6)	Cr9—Al40—Al25^i^	151.78 (6)
Al18^vi^—Al3—Al46^iv^	93.38 (6)	Cr13—Al40—Al25^i^	114.17 (6)
Al19^vi^—Al3—Al46^iv^	151.89 (7)	Al53—Al40—Al25^i^	89.16 (6)
Al37^iv^—Al3—Al46^iv^	110.07 (6)	Al12—Al40—Al25^i^	144.09 (6)
Al9^iv^—Al3—Al46^iv^	61.62 (5)	Al62^i^—Al40—Al25^i^	59.93 (5)
Cr10^iv^—Al3—Al10	54.62 (4)	Al23^ix^—Al40—Al25^i^	108.83 (6)
Cr5^vi^—Al3—Al10	114.44 (6)	Al32^xi^—Al40—Al25^i^	59.01 (5)
Al18^vi^—Al3—Al10	149.55 (7)	Al38^xi^—Al40—Al25^i^	89.45 (6)
Al19^vi^—Al3—Al10	147.85 (7)	Al16—Al40—Al25^i^	91.79 (6)
Al37^iv^—Al3—Al10	108.42 (6)	Al59^xi^—Al40—Al25^i^	60.72 (5)
Al9^iv^—Al3—Al10	109.18 (6)	Cr9—Al40—Al38	57.15 (4)
Al46^iv^—Al3—Al10	59.89 (5)	Cr13—Al40—Al38	57.59 (4)
Cr10^iv^—Al3—Al41^iv^	60.58 (5)	Al53—Al40—Al38	58.21 (5)
Cr5^vi^—Al3—Al41^iv^	117.09 (6)	Al12—Al40—Al38	58.39 (4)
Al18^vi^—Al3—Al41^iv^	150.31 (7)	Al62^i^—Al40—Al38	106.93 (6)
Al19^vi^—Al3—Al41^iv^	91.80 (6)	Al23^ix^—Al40—Al38	105.94 (6)
Al37^iv^—Al3—Al41^iv^	61.58 (5)	Al32^xi^—Al40—Al38	144.61 (7)
Al9^iv^—Al3—Al41^iv^	111.19 (6)	Al38^xi^—Al40—Al38	89.88 (6)
Al46^iv^—Al3—Al41^iv^	109.88 (6)	Al16—Al40—Al38	107.64 (6)
Al10—Al3—Al41^iv^	59.85 (5)	Al59^xi^—Al40—Al38	89.33 (6)
Cr10^iv^—Al3—Al6^xiii^	114.81 (6)	Al25^i^—Al40—Al38	145.23 (7)
Cr5^vi^—Al3—Al6^xiii^	54.21 (4)	Cr14^iv^—Al41—Cr10	58.97 (4)
Al18^vi^—Al3—Al6^xiii^	109.05 (6)	Cr14^iv^—Al41—Al57^xvi^	52.97 (4)
Al19^vi^—Al3—Al6^xiii^	109.05 (6)	Cr10—Al41—Al57^xvi^	103.01 (6)
Al37^iv^—Al3—Al6^xiii^	149.42 (7)	Cr14^iv^—Al41—Al32^iv^	150.11 (7)
Al9^iv^—Al3—Al6^xiii^	148.00 (7)	Cr10—Al41—Al32^iv^	112.83 (6)
Al46^iv^—Al3—Al6^xiii^	89.21 (6)	Al57^xvi^—Al41—Al32^iv^	144.12 (7)
Al10—Al3—Al6^xiii^	60.24 (5)	Cr14^iv^—Al41—Al25^xvii^	149.37 (7)
Al41^iv^—Al3—Al6^xiii^	90.01 (6)	Cr10—Al41—Al25^xvii^	112.18 (6)
Cr10^iv^—Al3—Al39^xiii^	116.63 (6)	Al57^xvi^—Al41—Al25^xvii^	108.43 (6)
Cr5^vi^—Al3—Al39^xiii^	58.58 (4)	Al32^iv^—Al41—Al25^xvii^	59.81 (5)
Al18^vi^—Al3—Al39^xiii^	61.24 (5)	Cr14^iv^—Al41—Al61^iv^	53.19 (4)
Al19^vi^—Al3—Al39^xiii^	110.42 (6)	Cr10—Al41—Al61^iv^	102.25 (5)
Al37^iv^—Al3—Al39^xiii^	150.20 (7)	Al57^xvi^—Al41—Al61^iv^	61.17 (5)
Al9^iv^—Al3—Al39^xiii^	91.86 (6)	Al32^iv^—Al41—Al61^iv^	107.72 (6)
Al46^iv^—Al3—Al39^xiii^	59.78 (5)	Al25^xvii^—Al41—Al61^iv^	145.56 (6)
Al10—Al3—Al39^xiii^	90.80 (6)	Cr14^iv^—Al41—Al10^iv^	102.03 (6)
Al41^iv^—Al3—Al39^xiii^	146.87 (7)	Cr10—Al41—Al10^iv^	52.41 (4)
Al6^xiii^—Al3—Al39^xiii^	60.12 (5)	Al57^xvi^—Al41—Al10^iv^	113.44 (6)
Cr10^iv^—Al3—Al32	119.35 (6)	Al32^iv^—Al41—Al10^iv^	90.63 (6)
Cr5^vi^—Al3—Al32	58.26 (4)	Al25^xvii^—Al41—Al10^iv^	60.10 (5)
Al18^vi^—Al3—Al32	110.35 (6)	Al61^iv^—Al41—Al10^iv^	153.80 (7)
Al19^vi^—Al3—Al32	61.11 (5)	Cr14^iv^—Al41—Al3^iv^	102.17 (5)
Al37^iv^—Al3—Al32	93.22 (6)	Cr10—Al41—Al3^iv^	52.03 (4)
Al9^iv^—Al3—Al32	151.34 (7)	Al57^xvi^—Al41—Al3^iv^	154.06 (7)
Al46^iv^—Al3—Al32	145.70 (7)	Al32^iv^—Al41—Al3^iv^	61.08 (5)
Al10—Al3—Al32	89.50 (6)	Al25^xvii^—Al41—Al3^iv^	90.06 (6)
Al41^iv^—Al3—Al32	59.08 (5)	Al61^iv^—Al41—Al3^iv^	112.58 (6)
Al6^xiii^—Al3—Al32	60.27 (5)	Al10^iv^—Al41—Al3^iv^	59.81 (5)
Al39^xiii^—Al3—Al32	109.93 (6)	Cr14^iv^—Al41—Al37	58.77 (4)
Al49—Al4—Al52	55.48 (5)	Cr10—Al41—Al37	58.33 (4)
Al49—Al4—Al4^iv^	152.09 (8)	Al57^xvi^—Al41—Al37	105.87 (6)
Al52—Al4—Al4^iv^	152.44 (8)	Al32^iv^—Al41—Al37	91.93 (6)
Al49—Al4—Al15^iv^	114.87 (6)	Al25^xvii^—Al41—Al37	145.69 (7)
Al52—Al4—Al15^iv^	114.15 (6)	Al61^iv^—Al41—Al37	57.00 (5)
Al4^iv^—Al4—Al15^iv^	61.91 (4)	Al10^iv^—Al41—Al37	105.12 (6)
Al49—Al4—Al13	114.35 (6)	Al3^iv^—Al41—Al37	57.42 (5)
Al52—Al4—Al13	115.20 (6)	Cr14^iv^—Al41—Al38^iv^	111.02 (6)
Al4^iv^—Al4—Al13	61.88 (4)	Cr10—Al41—Al38^iv^	149.33 (6)
Al15^iv^—Al4—Al13	123.79 (6)	Al57^xvi^—Al41—Al38^iv^	88.39 (5)
Al49—Al4—Al33	59.35 (5)	Al32^iv^—Al41—Al38^iv^	59.76 (5)
Al52—Al4—Al33	88.55 (5)	Al25^xvii^—Al41—Al38^iv^	90.24 (6)
Al4^iv^—Al4—Al33	107.38 (4)	Al61^iv^—Al41—Al38^iv^	58.34 (5)
Al15^iv^—Al4—Al33	56.13 (4)	Al10^iv^—Al41—Al38^iv^	146.93 (7)
Al13—Al4—Al33	147.03 (6)	Al3^iv^—Al41—Al38^iv^	110.13 (6)
Al49—Al4—Al31	88.89 (5)	Al37—Al41—Al38^iv^	91.28 (6)
Al52—Al4—Al31	59.76 (5)	Cr14^iv^—Al41—Al21	59.07 (4)
Al4^iv^—Al4—Al31	107.40 (4)	Cr10—Al41—Al21	60.32 (4)
Al15^iv^—Al4—Al31	147.13 (6)	Al57^xvi^—Al41—Al21	56.89 (5)
Al13—Al4—Al31	56.06 (4)	Al32^iv^—Al41—Al21	146.02 (6)
Al33—Al4—Al31	145.22 (6)	Al25^xvii^—Al41—Al21	90.65 (6)
Al49—Al4—Al15	95.29 (6)	Al61^iv^—Al41—Al21	106.24 (6)
Al52—Al4—Al15	142.23 (6)	Al10^iv^—Al41—Al21	58.03 (5)
Al4^iv^—Al4—Al15	59.87 (4)	Al3^iv^—Al41—Al21	106.38 (6)
Al15^iv^—Al4—Al15	52.54 (6)	Al37—Al41—Al21	107.75 (6)
Al13—Al4—Al15	97.80 (5)	Al38^iv^—Al41—Al21	143.48 (7)
Al33—Al4—Al15	54.09 (4)	Cr14^iv^—Al41—Al59^iv^	110.40 (6)
Al31—Al4—Al15	152.33 (6)	Cr10—Al41—Al59^iv^	149.80 (7)
Al49—Al4—Al13^iv^	142.53 (6)	Al57^xvi^—Al41—Al59^iv^	57.80 (5)
Al52—Al4—Al13^iv^	95.62 (5)	Al32^iv^—Al41—Al59^iv^	89.69 (6)
Al4^iv^—Al4—Al13^iv^	59.97 (4)	Al25^xvii^—Al41—Al59^iv^	60.97 (5)
Al15^iv^—Al4—Al13^iv^	97.89 (5)	Al61^iv^—Al41—Al59^iv^	88.76 (5)
Al13—Al4—Al13^iv^	52.47 (5)	Al10^iv^—Al41—Al59^iv^	110.40 (6)
Al33—Al4—Al13^iv^	152.52 (6)	Al3^iv^—Al41—Al59^iv^	147.40 (7)
Al31—Al4—Al13^iv^	54.05 (4)	Al37—Al41—Al59^iv^	144.43 (6)
Al15—Al4—Al13^iv^	119.84 (6)	Al38^iv^—Al41—Al59^iv^	59.38 (5)
Al49—Al4—Al11	141.27 (6)	Al21—Al41—Al59^iv^	89.68 (6)
Al52—Al4—Al11	94.07 (6)	Al56^i^—Al42—Cr8	120.05 (6)
Al4^iv^—Al4—Al11	61.35 (5)	Al56^i^—Al42—Al1^i^	141.38 (7)
Al15^iv^—Al4—Al11	51.82 (4)	Cr8—Al42—Al1^i^	54.15 (4)
Al13—Al4—Al11	99.54 (6)	Al56^i^—Al42—Al27^i^	153.40 (7)
Al33—Al4—Al11	101.25 (6)	Cr8—Al42—Al27^i^	56.56 (4)
Al31—Al4—Al11	95.38 (6)	Al1^i^—Al42—Al27^i^	61.70 (5)
Al15—Al4—Al11	98.22 (5)	Al56^i^—Al42—Al39^i^	119.38 (7)
Al13^iv^—Al4—Al11	51.43 (4)	Cr8—Al42—Al39^i^	117.08 (6)
Al49—Al4—Al11^iv^	94.02 (6)	Al1^i^—Al42—Al39^i^	89.19 (5)
Al52—Al4—Al11^iv^	141.38 (6)	Al27^i^—Al42—Al39^i^	61.25 (5)
Al4^iv^—Al4—Al11^iv^	61.17 (5)	Al56^i^—Al42—Al47^xix^	59.09 (5)
Al15^iv^—Al4—Al11^iv^	99.42 (6)	Cr8—Al42—Al47^xix^	153.27 (7)
Al13—Al4—Al11^iv^	51.87 (5)	Al1^i^—Al42—Al47^xix^	145.53 (6)
Al33—Al4—Al11^iv^	95.23 (6)	Al27^i^—Al42—Al47^xix^	110.83 (6)
Al31—Al4—Al11^iv^	101.25 (6)	Al39^i^—Al42—Al47^xix^	60.95 (5)
Al15—Al4—Al11^iv^	51.23 (4)	Al56^i^—Al42—Al63	62.43 (5)
Al13^iv^—Al4—Al11^iv^	98.20 (5)	Cr8—Al42—Al63	58.07 (4)
Al11—Al4—Al11^iv^	122.51 (5)	Al1^i^—Al42—Al63	103.37 (5)
Al49—Cr15—Al52	55.48 (5)	Al27^i^—Al42—Al63	105.04 (6)
Al49—Cr15—Cr18	114.35 (6)	Al39^i^—Al42—Al63	154.41 (7)
Al52—Cr15—Cr18	115.20 (6)	Al47^xix^—Al42—Al63	110.92 (6)
Al49—Cr15—Al33	59.35 (5)	Al56^i^—Al42—Al43^ii^	111.02 (7)
Al52—Cr15—Al33	88.55 (5)	Cr8—Al42—Al43^ii^	113.41 (6)
Cr18—Cr15—Al33	147.03 (6)	Al1^i^—Al42—Al43^ii^	59.26 (4)
Al49—Cr15—Al31	88.89 (5)	Al27^i^—Al42—Al43^ii^	92.33 (6)
Al52—Cr15—Al31	59.76 (5)	Al39^i^—Al42—Al43^ii^	59.63 (5)
Cr18—Cr15—Al31	56.06 (4)	Al47^xix^—Al42—Al43^ii^	89.09 (6)
Al33—Cr15—Al31	145.22 (6)	Al63—Al42—Al43^ii^	145.79 (7)
Al49—Cr15—Cr19	95.29 (6)	Al56^i^—Al42—Al60^i^	60.31 (5)
Al52—Cr15—Cr19	142.23 (6)	Cr8—Al42—Al60^i^	112.82 (6)
Cr18—Cr15—Cr19	97.80 (5)	Al1^i^—Al42—Al60^i^	85.77 (6)
Al33—Cr15—Cr19	54.09 (4)	Al27^i^—Al42—Al60^i^	146.16 (7)
Al31—Cr15—Cr19	152.33 (6)	Al39^i^—Al42—Al60^i^	112.77 (6)
Al49—Cr15—Cr17	141.27 (6)	Al47^xix^—Al42—Al60^i^	90.31 (6)
Al52—Cr15—Cr17	94.07 (6)	Al63—Al42—Al60^i^	90.61 (5)
Cr18—Cr15—Cr17	99.54 (6)	Al43^ii^—Al42—Al60^i^	60.82 (4)
Al33—Cr15—Cr17	101.25 (6)	Al56^i^—Al42—Al17^i^	96.43 (6)
Al31—Cr15—Cr17	95.38 (6)	Cr8—Al42—Al17^i^	61.88 (4)
Cr19—Cr15—Cr17	98.22 (5)	Al1^i^—Al42—Al17^i^	108.15 (6)
Cr5—Al5—Cr13	178.05 (7)	Al27^i^—Al42—Al17^i^	57.79 (5)
Cr5—Al5—Cr9	121.05 (6)	Al39^i^—Al42—Al17^i^	93.06 (6)
Cr13—Al5—Cr9	60.84 (4)	Al47^xix^—Al42—Al17^i^	91.38 (6)
Cr5—Al5—Al2	60.45 (5)	Al63—Al42—Al17^i^	61.99 (5)
Cr13—Al5—Al2	117.66 (6)	Al43^ii^—Al42—Al17^i^	148.01 (7)
Cr9—Al5—Al2	178.42 (7)	Al60^i^—Al42—Al17^i^	151.14 (6)
Cr5—Al5—Cr7^vi^	60.67 (4)	Cr8—Al43—Al55	120.15 (6)
Cr13—Al5—Cr7^vi^	118.23 (6)	Cr8—Al43—Al1^i^	54.14 (4)
Cr9—Al5—Cr7^vi^	120.41 (6)	Al55—Al43—Al1^i^	141.28 (7)
Al2—Al5—Cr7^vi^	59.61 (4)	Cr8—Al43—Al30	56.22 (4)
Cr5—Al5—Cr6^i^	61.54 (4)	Al55—Al43—Al30	153.54 (7)
Cr13—Al5—Cr6^i^	118.21 (6)	Al1^i^—Al43—Al30	61.58 (5)
Cr9—Al5—Cr6^i^	121.08 (6)	Cr8—Al43—Al39^iii^	116.44 (6)
Al2—Al5—Cr6^i^	59.83 (5)	Al55—Al43—Al39^iii^	119.78 (7)
Cr7^vi^—Al5—Cr6^i^	110.25 (5)	Al1^i^—Al43—Al39^iii^	89.11 (5)
Cr5—Al5—Al18	64.01 (5)	Al30—Al43—Al39^iii^	60.88 (5)
Cr13—Al5—Al18	117.79 (6)	Cr8—Al43—Al46	152.90 (7)
Cr9—Al5—Al18	67.06 (5)	Al55—Al43—Al46	59.22 (5)
Al2—Al5—Al18	114.45 (7)	Al1^i^—Al43—Al46	145.68 (6)
Cr7^vi^—Al5—Al18	116.13 (6)	Al30—Al43—Al46	110.84 (6)
Cr6^i^—Al5—Al18	64.87 (5)	Al39^iii^—Al43—Al46	61.21 (5)
Cr5—Al5—Al14	115.23 (6)	Cr8—Al43—Al63	58.19 (4)
Cr13—Al5—Al14	64.84 (5)	Al55—Al43—Al63	62.36 (5)
Cr9—Al5—Al14	64.43 (5)	Al1^i^—Al43—Al63	103.28 (5)
Al2—Al5—Al14	114.69 (7)	Al30—Al43—Al63	105.05 (6)
Cr7^vi^—Al5—Al14	64.42 (5)	Al39^iii^—Al43—Al63	154.16 (7)
Cr6^i^—Al5—Al14	174.36 (7)	Al46—Al43—Al63	110.87 (6)
Al18—Al5—Al14	118.60 (7)	Cr8—Al43—Al42^ii^	113.15 (6)
Cr5—Al5—Al16	115.64 (6)	Al55—Al43—Al42^ii^	111.45 (7)
Cr13—Al5—Al16	65.31 (5)	Al1^i^—Al43—Al42^ii^	59.02 (4)
Cr9—Al5—Al16	65.26 (5)	Al30—Al43—Al42^ii^	91.87 (6)
Al2—Al5—Al16	114.78 (7)	Al39^iii^—Al43—Al42^ii^	59.72 (5)
Cr7^vi^—Al5—Al16	174.10 (7)	Al46—Al43—Al42^ii^	89.45 (6)
Cr6^i^—Al5—Al16	64.05 (5)	Al63—Al43—Al42^ii^	145.88 (7)
Al18—Al5—Al16	63.58 (5)	Cr8—Al43—Al60^i^	113.19 (5)
Al14—Al5—Al16	121.21 (7)	Al55—Al43—Al60^i^	60.38 (5)
Cr5—Al5—Al19	63.53 (5)	Al1^i^—Al43—Al60^i^	85.72 (6)
Cr13—Al5—Al19	117.72 (6)	Al30—Al43—Al60^i^	145.94 (7)
Cr9—Al5—Al19	66.74 (5)	Al39^iii^—Al43—Al60^i^	112.98 (6)
Al2—Al5—Al19	114.22 (7)	Al46—Al43—Al60^i^	90.40 (6)
Cr7^vi^—Al5—Al19	64.65 (5)	Al63—Al43—Al60^i^	90.77 (5)
Cr6^i^—Al5—Al19	116.33 (6)	Al42^ii^—Al43—Al60^i^	60.97 (4)
Al18—Al5—Al19	63.91 (5)	Cr8—Al43—Al8	61.30 (4)
Al14—Al5—Al19	63.78 (5)	Al55—Al43—Al8	96.60 (6)
Al16—Al5—Al19	118.63 (7)	Al1^i^—Al43—Al8	107.81 (6)
Cr5—Al5—Al28	113.93 (6)	Al30—Al43—Al8	57.76 (5)
Cr13—Al5—Al28	64.82 (5)	Al39^iii^—Al43—Al8	92.93 (6)
Cr9—Al5—Al28	115.44 (6)	Al46—Al43—Al8	91.59 (6)
Al2—Al5—Al28	63.63 (5)	Al63—Al43—Al8	61.84 (5)
Cr7^vi^—Al5—Al28	114.30 (6)	Al42^ii^—Al43—Al8	147.70 (7)
Cr6^i^—Al5—Al28	61.59 (5)	Al60^i^—Al43—Al8	151.26 (6)
Al18—Al5—Al28	115.38 (6)	Al13—Al44—Cr11^iv^	56.09 (4)
Al14—Al5—Al28	118.12 (7)	Cr18—Al44—Al17	147.10 (7)
Al16—Al5—Al28	62.19 (5)	Al13—Al44—Al17	147.10 (7)
Al19—Al5—Al28	177.46 (7)	Cr11^iv^—Al44—Al17	145.51 (7)
Cr5—Al5—Al29	113.73 (6)	Cr18—Al44—Al35	99.03 (6)
Cr13—Al5—Al29	64.47 (5)	Al13—Al44—Al35	99.03 (6)
Cr9—Al5—Al29	114.94 (6)	Cr11^iv^—Al44—Al35	117.01 (6)
Al2—Al5—Al29	63.57 (5)	Al17—Al44—Al35	88.48 (6)
Cr7^vi^—Al5—Al29	62.09 (4)	Cr18—Al44—Al6	100.03 (6)
Cr6^i^—Al5—Al29	114.09 (6)	Al13—Al44—Al6	100.03 (6)
Al18—Al5—Al29	177.73 (7)	Cr11^iv^—Al44—Al6	52.50 (4)
Al14—Al5—Al29	62.27 (5)	Al17—Al44—Al6	112.58 (7)
Al16—Al5—Al29	118.02 (7)	Al35—Al44—Al6	84.84 (6)
Al19—Al5—Al29	115.58 (6)	Al13—Al44—Al45^iv^	57.19 (5)
Al28—Al5—Al29	65.02 (5)	Cr11^iv^—Al44—Al45^iv^	59.13 (4)
Cr5—Cr16—Cr13	178.05 (7)	Al17—Al44—Al45^iv^	146.21 (7)
Cr5—Cr16—Cr9	121.05 (6)	Al35—Al44—Al45^iv^	59.31 (5)
Cr13—Cr16—Cr9	60.84 (4)	Al6—Al44—Al45^iv^	58.72 (5)
Cr5—Cr16—Al2	60.45 (5)	Cr18—Al44—Cr3	54.52 (4)
Cr13—Cr16—Al2	117.66 (6)	Al13—Al44—Cr3	54.52 (4)
Cr9—Cr16—Al2	178.42 (7)	Cr11^iv^—Al44—Cr3	103.58 (5)
Cr5—Cr16—Cr7^vi^	60.67 (4)	Al17—Al44—Cr3	110.59 (6)
Cr13—Cr16—Cr7^vi^	118.23 (6)	Al35—Al44—Cr3	52.32 (4)
Cr9—Cr16—Cr7^vi^	120.41 (6)	Al6—Al44—Cr3	116.49 (6)
Al2—Cr16—Cr7^vi^	59.61 (4)	Al45^iv^—Al44—Cr3	59.28 (4)
Cr5—Cr16—Cr6^i^	61.54 (4)	Al13—Al44—Al47^iv^	101.11 (6)
Cr13—Cr16—Cr6^i^	118.21 (6)	Cr11^iv^—Al44—Al47^iv^	53.65 (4)
Cr9—Cr16—Cr6^i^	121.08 (6)	Al17—Al44—Al47^iv^	91.88 (6)
Al2—Cr16—Cr6^i^	59.83 (5)	Al35—Al44—Al47^iv^	141.72 (7)
Cr7^vi^—Cr16—Cr6^i^	110.25 (5)	Al6—Al44—Al47^iv^	59.83 (5)
Cr5—Cr16—Al18	64.01 (5)	Al45^iv^—Al44—Al47^iv^	106.95 (6)
Cr13—Cr16—Al18	117.79 (6)	Cr3—Al44—Al47^iv^	155.42 (7)
Cr9—Cr16—Al18	67.06 (5)	Cr18—Al44—Al36	95.77 (6)
Al2—Cr16—Al18	114.45 (7)	Al13—Al44—Al36	95.77 (6)
Cr7^vi^—Cr16—Al18	116.13 (6)	Cr11^iv^—Al44—Al36	151.62 (7)
Cr6^i^—Cr16—Al18	64.87 (5)	Al17—Al44—Al36	60.87 (5)
Cr5—Cr16—Al14	115.23 (6)	Al35—Al44—Al36	58.96 (5)
Cr13—Cr16—Al14	64.84 (5)	Al6—Al44—Al36	142.38 (7)
Cr9—Cr16—Al14	64.43 (5)	Al45^iv^—Al44—Al36	104.52 (6)
Al2—Cr16—Al14	114.69 (7)	Cr3—Al44—Al36	50.09 (4)
Cr7^vi^—Cr16—Al14	64.42 (5)	Al47^iv^—Al44—Al36	148.53 (7)
Cr6^i^—Cr16—Al14	174.36 (7)	Cr18—Al44—Al26	146.14 (7)
Al18—Cr16—Al14	118.60 (7)	Al13—Al44—Al26	146.14 (7)
Cr5—Cr16—Al16	115.64 (6)	Cr11^iv^—Al44—Al26	109.76 (6)
Cr13—Cr16—Al16	65.31 (5)	Al17—Al44—Al26	63.02 (5)
Cr9—Cr16—Al16	65.26 (5)	Al35—Al44—Al26	57.49 (5)
Al2—Cr16—Al16	114.78 (7)	Al6—Al44—Al26	57.38 (5)
Cr7^vi^—Cr16—Al16	174.10 (7)	Al45^iv^—Al44—Al26	88.97 (6)
Cr6^i^—Cr16—Al16	64.05 (5)	Cr3—Al44—Al26	109.73 (6)
Al18—Cr16—Al16	63.58 (5)	Al47^iv^—Al44—Al26	88.94 (6)
Al14—Cr16—Al16	121.21 (7)	Al36—Al44—Al26	91.65 (6)
Cr5—Cr16—Al19	63.53 (5)	Al13^iv^—Al45—Al62	104.02 (6)
Cr13—Cr16—Al19	117.72 (6)	Al13^iv^—Al45—Cr11	56.43 (4)
Cr9—Cr16—Al19	66.74 (5)	Al62—Al45—Cr11	104.38 (6)
Al2—Cr16—Al19	114.22 (7)	Al13^iv^—Al45—Cr12	57.18 (4)
Cr7^vi^—Cr16—Al19	64.65 (5)	Al62—Al45—Cr12	53.76 (4)
Cr6^i^—Cr16—Al19	116.33 (6)	Cr11—Al45—Cr12	57.78 (4)
Al18—Cr16—Al19	63.91 (5)	Al13^iv^—Al45—Al6^iv^	102.41 (6)
Al14—Cr16—Al19	63.78 (5)	Al62—Al45—Al6^iv^	119.86 (7)
Al16—Cr16—Al19	118.63 (7)	Cr11—Al45—Al6^iv^	52.85 (4)
Cr5—Cr16—Al28	113.93 (6)	Cr12—Al45—Al6^iv^	103.23 (6)
Cr13—Cr16—Al28	64.82 (5)	Al13^iv^—Al45—Al35^iv^	100.68 (6)
Cr9—Cr16—Al28	115.44 (6)	Al62—Al45—Al35^iv^	138.37 (7)
Al2—Cr16—Al28	63.63 (5)	Cr11—Al45—Al35^iv^	117.18 (6)
Cr7^vi^—Cr16—Al28	114.30 (6)	Cr12—Al45—Al35^iv^	157.14 (7)
Cr6^i^—Cr16—Al28	61.59 (5)	Al6^iv^—Al45—Al35^iv^	86.09 (6)
Al18—Cr16—Al28	115.38 (6)	Al13^iv^—Al45—Al31	58.22 (5)
Al14—Cr16—Al28	118.12 (7)	Al62—Al45—Al31	62.02 (5)
Al16—Cr16—Al28	62.19 (5)	Cr11—Al45—Al31	105.38 (6)
Al19—Cr16—Al28	177.46 (7)	Cr12—Al45—Al31	59.00 (4)
Cr5—Cr16—Al29	113.73 (6)	Al6^iv^—Al45—Al31	158.20 (7)
Cr13—Cr16—Al29	64.47 (5)	Al35^iv^—Al45—Al31	106.23 (6)
Cr9—Cr16—Al29	114.94 (6)	Al13^iv^—Al45—Cr3	55.65 (4)
Al2—Cr16—Al29	63.57 (5)	Al62—Al45—Cr3	119.65 (6)
Cr7^vi^—Cr16—Al29	62.09 (4)	Cr11—Al45—Cr3	104.70 (5)
Cr6^i^—Cr16—Al29	114.09 (6)	Cr12—Al45—Cr3	104.87 (5)
Al18—Cr16—Al29	177.73 (7)	Al6^iv^—Al45—Cr3	119.97 (6)
Al14—Cr16—Al29	62.27 (5)	Al35^iv^—Al45—Cr3	53.10 (4)
Al16—Cr16—Al29	118.02 (7)	Al31—Al45—Cr3	59.77 (4)
Al19—Cr16—Al29	115.58 (6)	Al13^iv^—Al45—Al44^iv^	58.80 (5)
Al28—Cr16—Al29	65.02 (5)	Al62—Al45—Al44^iv^	160.05 (7)
Cr11^iv^—Al6—Cr5^xiv^	168.38 (7)	Cr11—Al45—Al44^iv^	58.62 (4)
Cr11^iv^—Al6—Al45^iv^	63.11 (5)	Cr12—Al45—Al44^iv^	106.29 (6)
Cr5^xiv^—Al6—Al45^iv^	126.94 (7)	Al6^iv^—Al45—Al44^iv^	60.33 (5)
Cr11^iv^—Al6—Al26	126.05 (7)	Al35^iv^—Al45—Al44^iv^	60.00 (5)
Cr5^xiv^—Al6—Al26	62.36 (5)	Al31—Al45—Al44^iv^	109.87 (6)
Al45^iv^—Al6—Al26	94.30 (6)	Cr3—Al45—Al44^iv^	61.26 (4)
Cr11^iv^—Al6—Al24^xiv^	127.98 (6)	Al13^iv^—Al45—Al25	102.79 (6)
Cr5^xiv^—Al6—Al24^xiv^	62.12 (5)	Al62—Al45—Al25	60.78 (5)
Al45^iv^—Al6—Al24^xiv^	64.94 (5)	Cr11—Al45—Al25	57.36 (4)
Al26—Al6—Al24^xiv^	60.27 (5)	Cr12—Al45—Al25	56.57 (4)
Cr11^iv^—Al6—Al44	62.01 (5)	Al6^iv^—Al45—Al25	61.15 (5)
Cr5^xiv^—Al6—Al44	126.38 (6)	Al35^iv^—Al45—Al25	142.96 (7)
Al45^iv^—Al6—Al44	60.95 (5)	Al31—Al45—Al25	110.44 (6)
Al26—Al6—Al44	64.21 (5)	Cr3—Al45—Al25	158.43 (7)
Al24^xiv^—Al6—Al44	94.85 (6)	Al44^iv^—Al45—Al25	110.40 (6)
Cr11^iv^—Al6—Al47^iv^	56.88 (4)	Al13^iv^—Al45—Al36	97.60 (6)
Cr5^xiv^—Al6—Al47^iv^	117.70 (6)	Al62—Al45—Al36	85.31 (6)
Al45^iv^—Al6—Al47^iv^	110.43 (6)	Cr11—Al45—Al36	153.59 (6)
Al26—Al6—Al47^iv^	94.08 (6)	Cr12—Al45—Al36	114.77 (6)
Al24^xiv^—Al6—Al47^iv^	152.22 (7)	Al6^iv^—Al45—Al36	141.99 (7)
Al44—Al6—Al47^iv^	61.99 (5)	Al35^iv^—Al45—Al36	58.43 (5)
Cr11^iv^—Al6—Al3^xiii^	113.94 (6)	Al31—Al45—Al36	57.13 (5)
Cr5^xiv^—Al6—Al3^xiii^	54.51 (4)	Cr3—Al45—Al36	50.38 (4)
Al45^iv^—Al6—Al3^xiii^	149.12 (7)	Al44^iv^—Al45—Al36	105.90 (6)
Al26—Al6—Al3^xiii^	108.85 (6)	Al25—Al45—Al36	143.64 (6)
Al24^xiv^—Al6—Al3^xiii^	108.83 (6)	Cr10—Al46—Al55	123.24 (6)
Al44—Al6—Al3^xiii^	147.89 (7)	Cr10—Al46—Al47^xvii^	114.76 (6)
Al47^iv^—Al6—Al3^xiii^	88.57 (6)	Al55—Al46—Al47^xvii^	111.69 (6)
Cr11^iv^—Al6—Al10^xiii^	54.38 (4)	Cr10—Al46—Al43	147.49 (7)
Cr5^xiv^—Al6—Al10^xiii^	114.12 (6)	Al55—Al46—Al43	57.10 (5)
Al45^iv^—Al6—Al10^xiii^	108.43 (6)	Al47^xvii^—Al46—Al43	90.64 (6)
Al26—Al6—Al10^xiii^	149.64 (7)	Cr10—Al46—Al64	60.50 (4)
Al24^xiv^—Al6—Al10^xiii^	147.97 (7)	Al55—Al46—Al64	62.75 (5)
Al44—Al6—Al10^xiii^	109.30 (6)	Al47^xvii^—Al46—Al64	147.28 (7)
Al47^iv^—Al6—Al10^xiii^	59.63 (5)	Al43—Al46—Al64	108.65 (6)
Al3^xiii^—Al6—Al10^xiii^	59.62 (5)	Cr10—Al46—Al10^iv^	53.92 (4)
Cr11^iv^—Al6—Al25^iv^	60.42 (5)	Al55—Al46—Al10^iv^	144.62 (7)
Cr5^xiv^—Al6—Al25^iv^	117.07 (6)	Al47^xvii^—Al46—Al10^iv^	61.04 (5)
Al45^iv^—Al6—Al25^iv^	61.61 (5)	Al43—Al46—Al10^iv^	147.80 (7)
Al26—Al6—Al25^iv^	150.58 (7)	Al64—Al46—Al10^iv^	103.55 (6)
Al24^xiv^—Al6—Al25^iv^	92.55 (6)	Cr10—Al46—Al3^iv^	53.68 (4)
Al44—Al6—Al25^iv^	111.26 (6)	Al55—Al46—Al3^iv^	152.35 (7)
Al47^iv^—Al6—Al25^iv^	109.54 (6)	Al47^xvii^—Al46—Al3^iv^	90.94 (6)
Al3^xiii^—Al6—Al25^iv^	89.57 (6)	Al43—Al46—Al3^iv^	109.17 (6)
Al10^xiii^—Al6—Al25^iv^	59.63 (5)	Al64—Al46—Al3^iv^	106.42 (6)
Cr11^iv^—Al6—Al39	116.27 (6)	Al10^iv^—Al46—Al3^iv^	60.21 (5)
Cr5^xiv^—Al6—Al39	58.65 (4)	Cr10—Al46—Al39^iii^	114.25 (6)
Al45^iv^—Al6—Al39	150.61 (7)	Al55—Al46—Al39^iii^	115.90 (6)
Al26—Al6—Al39	61.35 (5)	Al47^xvii^—Al46—Al39^iii^	60.91 (5)
Al24^xiv^—Al6—Al39	109.97 (6)	Al43—Al46—Al39^iii^	59.39 (5)
Al44—Al6—Al39	91.97 (6)	Al64—Al46—Al39^iii^	151.79 (7)
Al47^iv^—Al6—Al39	59.51 (5)	Al10^iv^—Al46—Al39^iii^	91.51 (6)
Al3^xiii^—Al6—Al39	60.08 (5)	Al3^iv^—Al46—Al39^iii^	60.67 (5)
Al10^xiii^—Al6—Al39	90.42 (6)	Cr10—Al46—Al58^iii^	119.32 (6)
Al25^iv^—Al6—Al39	146.35 (7)	Al55—Al46—Al58^iii^	60.70 (5)
Cr11^iv^—Al6—Al32^xiii^	119.02 (6)	Al47^xvii^—Al46—Al58^iii^	61.34 (5)
Cr5^xiv^—Al6—Al32^xiii^	58.43 (4)	Al43—Al46—Al58^iii^	90.05 (6)
Al45^iv^—Al6—Al32^xiii^	92.98 (6)	Al64—Al46—Al58^iii^	91.66 (6)
Al26—Al6—Al32^xiii^	109.88 (6)	Al10^iv^—Al46—Al58^iii^	89.08 (6)
Al24^xiv^—Al6—Al32^xiii^	61.03 (5)	Al3^iv^—Al46—Al58^iii^	146.93 (7)
Al44—Al6—Al32^xiii^	151.34 (7)	Al39^iii^—Al46—Al58^iii^	112.59 (6)
Al47^iv^—Al6—Al32^xiii^	145.25 (7)	Cr10—Al46—Al9	59.47 (4)
Al3^xiii^—Al6—Al32^xiii^	60.47 (5)	Al55—Al46—Al9	95.09 (6)
Al10^xiii^—Al6—Al32^xiii^	89.26 (6)	Al47^xvii^—Al46—Al9	147.04 (7)
Al25^iv^—Al6—Al32^xiii^	58.94 (5)	Al43—Al46—Al9	88.12 (6)
Al39—Al6—Al32^xiii^	110.06 (6)	Al64—Al46—Al9	62.27 (5)
Cr7^vi^—Al7—Cr4	164.76 (7)	Al10^iv^—Al46—Al9	107.12 (6)
Cr7^vi^—Al7—Al29	63.61 (5)	Al3^iv^—Al46—Al9	58.71 (5)
Cr4—Al7—Al29	131.62 (7)	Al39^iii^—Al46—Al9	90.68 (6)
Cr7^vi^—Al7—Al33	130.78 (7)	Al58^iii^—Al46—Al9	151.55 (7)
Cr4—Al7—Al33	64.45 (5)	Cr11—Al47—Al56^iv^	123.47 (6)
Al29—Al7—Al33	67.17 (5)	Cr11—Al47—Al46^xvii^	114.65 (6)
Cr7^vi^—Al7—Al34	113.65 (6)	Al56^iv^—Al47—Al46^xvii^	111.43 (6)
Cr4—Al7—Al34	55.44 (4)	Cr11—Al47—Al65^iv^	60.60 (5)
Al29—Al7—Al34	144.70 (7)	Al56^iv^—Al47—Al65^iv^	62.89 (5)
Al33—Al7—Al34	106.44 (6)	Al46^xvii^—Al47—Al65^iv^	146.99 (7)
Cr7^vi^—Al7—Al34^iv^	112.02 (6)	Cr11—Al47—Al42^xvi^	147.41 (7)
Cr4—Al7—Al34^iv^	55.14 (4)	Al56^iv^—Al47—Al42^xvi^	57.09 (5)
Al29—Al7—Al34^iv^	149.53 (7)	Al46^xvii^—Al47—Al42^xvi^	90.81 (6)
Al33—Al7—Al34^iv^	109.73 (6)	Al65^iv^—Al47—Al42^xvi^	108.67 (6)
Al34—Al7—Al34^iv^	65.74 (6)	Cr11—Al47—Al39^iv^	114.03 (6)
Cr7^vi^—Al7—Al20^vi^	54.87 (4)	Al56^iv^—Al47—Al39^iv^	115.95 (6)
Cr4—Al7—Al20^vi^	113.90 (6)	Al46^xvii^—Al47—Al39^iv^	61.17 (5)
Al29—Al7—Al20^vi^	106.18 (6)	Al65^iv^—Al47—Al39^iv^	151.82 (7)
Al33—Al7—Al20^vi^	146.43 (7)	Al42^xvi^—Al47—Al39^iv^	59.48 (5)
Al34—Al7—Al20^vi^	58.92 (5)	Cr11—Al47—Al10^iii^	53.89 (4)
Al34^iv^—Al7—Al20^vi^	92.09 (6)	Al56^iv^—Al47—Al10^iii^	144.33 (7)
Cr7^vi^—Al7—Al23	54.21 (4)	Al46^xvii^—Al47—Al10^iii^	60.98 (5)
Cr4—Al7—Al23	112.67 (6)	Al65^iv^—Al47—Al10^iii^	103.39 (6)
Al29—Al7—Al23	109.36 (6)	Al42^xvi^—Al47—Al10^iii^	147.94 (7)
Al33—Al7—Al23	151.31 (7)	Al39^iv^—Al47—Al10^iii^	91.62 (6)
Al34—Al7—Al23	92.06 (6)	Cr11—Al47—Al6^iv^	53.47 (4)
Al34^iv^—Al7—Al23	57.86 (5)	Al56^iv^—Al47—Al6^iv^	152.34 (7)
Al20^vi^—Al7—Al23	62.16 (5)	Al46^xvii^—Al47—Al6^iv^	91.28 (6)
Cr7^vi^—Al7—Al8	61.77 (5)	Al65^iv^—Al47—Al6^iv^	106.27 (6)
Cr4—Al7—Al8	120.67 (6)	Al42^xvi^—Al47—Al6^iv^	109.15 (6)
Al29—Al7—Al8	64.72 (5)	Al39^iv^—Al47—Al6^iv^	60.69 (5)
Al33—Al7—Al8	96.73 (6)	Al10^iii^—Al47—Al6^iv^	60.51 (5)
Al34—Al7—Al8	148.24 (7)	Cr11—Al47—Al58^iv^	119.47 (6)
Al34^iv^—Al7—Al8	86.31 (6)	Al56^iv^—Al47—Al58^iv^	60.64 (5)
Al20^vi^—Al7—Al8	110.25 (6)	Al46^xvii^—Al47—Al58^iv^	61.00 (5)
Al23—Al7—Al8	58.94 (5)	Al65^iv^—Al47—Al58^iv^	91.76 (6)
Cr7^vi^—Al7—Al9	118.62 (6)	Al42^xvi^—Al47—Al58^iv^	90.05 (6)
Cr4—Al7—Al9	63.94 (4)	Al39^iv^—Al47—Al58^iv^	112.52 (6)
Al29—Al7—Al9	95.77 (6)	Al10^iii^—Al47—Al58^iv^	88.86 (6)
Al33—Al7—Al9	65.38 (5)	Al6^iv^—Al47—Al58^iv^	147.00 (7)
Al34—Al7—Al9	113.44 (6)	Cr11—Al47—Al44^iv^	59.31 (4)
Al34^iv^—Al7—Al9	58.48 (5)	Al56^iv^—Al47—Al44^iv^	95.59 (6)
Al20^vi^—Al7—Al9	146.77 (7)	Al46^xvii^—Al47—Al44^iv^	146.93 (7)
Al23—Al7—Al9	87.35 (6)	Al65^iv^—Al47—Al44^iv^	62.57 (5)
Al8—Al7—Al9	57.33 (5)	Al42^xvi^—Al47—Al44^iv^	88.19 (6)
Cr7^vi^—Al7—Al14	61.64 (4)	Al39^iv^—Al47—Al44^iv^	90.43 (6)
Cr4—Al7—Al14	123.94 (6)	Al10^iii^—Al47—Al44^iv^	106.97 (6)
Al29—Al7—Al14	58.65 (5)	Al6^iv^—Al47—Al44^iv^	58.18 (5)
Al33—Al7—Al14	93.27 (6)	Al58^iv^—Al47—Al44^iv^	152.02 (7)
Al34—Al7—Al14	88.27 (6)	Cr13—Al48—Cr1	167.09 (7)
Al34^iv^—Al7—Al14	148.92 (7)	Cr13—Al48—Al29	64.38 (5)
Al20^vi^—Al7—Al14	58.65 (5)	Cr1—Al48—Al29	125.83 (6)
Al23—Al7—Al14	109.36 (6)	Cr13—Al48—Al28	64.47 (5)
Al8—Al7—Al14	112.18 (6)	Cr1—Al48—Al28	125.85 (6)
Al9—Al7—Al14	152.56 (7)	Al29—Al48—Al28	62.59 (5)
Cr7^vi^—Al7—Al37^iv^	123.92 (6)	Cr13—Al48—Al50^i^	122.17 (6)
Cr4—Al7—Al37^iv^	61.69 (4)	Cr1—Al48—Al50^i^	67.79 (5)
Al29—Al7—Al37^iv^	93.50 (6)	Al29—Al48—Al50^i^	93.65 (6)
Al33—Al7—Al37^iv^	59.04 (5)	Al28—Al48—Al50^i^	58.09 (5)
Al34—Al7—Al37^iv^	57.45 (5)	Cr13—Al48—Al61	57.17 (4)
Al34^iv^—Al7—Al37^iv^	111.10 (6)	Cr1—Al48—Al61	118.35 (6)
Al20^vi^—Al7—Al37^iv^	89.79 (6)	Al29—Al48—Al61	61.26 (5)
Al23—Al7—Al37^iv^	147.41 (7)	Al28—Al48—Al61	111.01 (6)
Al8—Al7—Al37^iv^	153.36 (7)	Al50^i^—Al48—Al61	153.46 (7)
Al9—Al7—Al37^iv^	113.85 (6)	Cr13—Al48—Al51	122.12 (7)
Al14—Al7—Al37^iv^	62.80 (5)	Cr1—Al48—Al51	67.66 (5)
Al2—Al8—Al30	100.44 (6)	Al29—Al48—Al51	58.19 (5)
Al2—Al8—Cr7^vi^	55.89 (4)	Al28—Al48—Al51	93.92 (6)
Al30—Al8—Cr7^vi^	118.46 (6)	Al50^i^—Al48—Al51	69.73 (5)
Al2—Al8—Al9	150.01 (7)	Al61—Al48—Al51	88.32 (6)
Al30—Al8—Al9	108.60 (6)	Cr13—Al48—Al62^i^	57.37 (4)
Cr7^vi^—Al8—Al9	113.14 (6)	Cr1—Al48—Al62^i^	118.39 (6)
Al2—Al8—Cr8	54.41 (4)	Al29—Al48—Al62^i^	110.92 (6)
Al30—Al8—Cr8	54.20 (4)	Al28—Al48—Al62^i^	61.07 (5)
Cr7^vi^—Al8—Cr8	104.05 (5)	Al50^i^—Al48—Al62^i^	88.30 (6)
Al9—Al8—Cr8	142.44 (7)	Al61—Al48—Al62^i^	107.91 (6)
Al2—Al8—Al23	99.05 (6)	Al51—Al48—Al62^i^	153.53 (7)
Al30—Al8—Al23	85.13 (6)	Cr13—Al48—Al52^i^	117.07 (6)
Cr7^vi^—Al8—Al23	52.08 (4)	Cr1—Al48—Al52^i^	58.17 (4)
Al9—Al8—Al23	90.84 (6)	Al29—Al48—Al52^i^	153.58 (7)
Cr8—Al8—Al23	117.11 (6)	Al28—Al48—Al52^i^	93.37 (6)
Al2—Al8—Al24	57.06 (5)	Al50^i^—Al48—Al52^i^	62.52 (5)
Al30—Al8—Al24	61.91 (5)	Al61—Al48—Al52^i^	143.80 (7)
Cr7^vi^—Al8—Al24	57.94 (4)	Al51—Al48—Al52^i^	117.23 (6)
Al9—Al8—Al24	145.79 (7)	Al62^i^—Al48—Al52^i^	60.37 (5)
Cr8—Al8—Al24	61.72 (4)	Cr13—Al48—Al49	116.90 (6)
Al23—Al8—Al24	56.86 (5)	Cr1—Al48—Al49	58.14 (4)
Al2—Al8—Al7	98.69 (6)	Al29—Al48—Al49	93.43 (6)
Al30—Al8—Al7	142.74 (7)	Al28—Al48—Al49	153.65 (7)
Cr7^vi^—Al8—Al7	51.63 (4)	Al50^i^—Al48—Al49	117.22 (7)
Al9—Al8—Al7	61.94 (5)	Al61—Al48—Al49	60.37 (5)
Cr8—Al8—Al7	153.05 (6)	Al51—Al48—Al49	62.32 (5)
Al23—Al8—Al7	60.39 (5)	Al62^i^—Al48—Al49	143.93 (7)
Al24—Al8—Al7	104.33 (6)	Al52^i^—Al48—Al49	107.24 (6)
Al2—Al8—Al43	101.06 (6)	Cr13—Al48—Al66	112.85 (6)
Al30—Al8—Al43	58.48 (5)	Cr1—Al48—Al66	54.24 (4)
Cr7^vi^—Al8—Al43	156.78 (6)	Al29—Al48—Al66	147.96 (7)
Al9—Al8—Al43	88.19 (6)	Al28—Al48—Al66	147.85 (7)
Cr8—Al8—Al43	54.26 (4)	Al50^i^—Al48—Al66	111.74 (6)
Al23—Al8—Al43	140.91 (7)	Al61—Al48—Al66	89.73 (6)
Al24—Al8—Al43	109.35 (6)	Al51—Al48—Al66	111.57 (6)
Al7—Al8—Al43	146.26 (7)	Al62^i^—Al48—Al66	89.81 (6)
Al2—Al8—Al63	56.60 (5)	Al52^i^—Al48—Al66	58.10 (5)
Al30—Al8—Al63	102.18 (6)	Al49—Al48—Al66	58.13 (5)
Cr7^vi^—Al8—Al63	104.84 (6)	Cr13—Al48—Al53	53.66 (4)
Al9—Al8—Al63	108.58 (7)	Cr1—Al48—Al53	113.43 (6)
Cr8—Al8—Al63	54.53 (4)	Al29—Al48—Al53	109.81 (6)
Al23—Al8—Al63	155.31 (7)	Al28—Al48—Al53	109.72 (6)
Al24—Al8—Al63	105.59 (6)	Al50^i^—Al48—Al53	144.96 (6)
Al7—Al8—Al63	115.05 (6)	Al61—Al48—Al53	59.49 (5)
Al43—Al8—Al63	57.41 (5)	Al51—Al48—Al53	144.89 (6)
Al2—Al8—Al18^xv^	147.05 (7)	Al62^i^—Al48—Al53	59.53 (5)
Al30—Al8—Al18^xv^	56.17 (5)	Al52^i^—Al48—Al53	87.73 (6)
Cr7^vi^—Al8—Al18^xv^	111.48 (6)	Al49—Al48—Al53	87.81 (5)
Al9—Al8—Al18^xv^	61.10 (5)	Al66—Al48—Al53	59.19 (5)
Cr8—Al8—Al18^xv^	110.21 (6)	Cr14—Al49—Al52	131.90 (7)
Al23—Al8—Al18^xv^	59.59 (5)	Cr14—Al49—Cr1	168.02 (7)
Al24—Al8—Al18^xv^	90.11 (6)	Al52—Al49—Cr1	60.08 (4)
Al7—Al8—Al18^xv^	91.75 (6)	Cr14—Al49—Al66	115.18 (6)
Al43—Al8—Al18^xv^	86.33 (5)	Al52—Al49—Al66	105.89 (6)
Al63—Al8—Al18^xv^	143.38 (7)	Cr1—Al49—Al66	55.09 (4)
Al15^iv^—Al9—Cr10	56.20 (4)	Cr14—Al49—Cr15	69.62 (5)
Al15^iv^—Al9—Al8	147.70 (7)	Al52—Al49—Cr15	62.29 (5)
Cr10—Al9—Al8	145.88 (7)	Cr1—Al49—Cr15	122.36 (6)
Al15^iv^—Al9—Al34^iv^	97.73 (6)	Al66—Al49—Cr15	148.24 (7)
Cr10—Al9—Al34^iv^	116.58 (6)	Cr14—Al49—Al4	69.62 (5)
Al8—Al9—Al34^iv^	88.46 (6)	Al52—Al49—Al4	62.29 (5)
Al15^iv^—Al9—Al3^iv^	100.07 (6)	Cr1—Al49—Al4	122.36 (6)
Cr10—Al9—Al3^iv^	52.49 (4)	Al66—Al49—Al4	148.24 (7)
Al8—Al9—Al3^iv^	112.05 (7)	Cr14—Al49—Al48	114.92 (6)
Al34^iv^—Al9—Al3^iv^	85.30 (6)	Al52—Al49—Al48	105.54 (6)
Al15^iv^—Al9—Al37	57.03 (5)	Cr1—Al49—Al48	55.61 (4)
Cr10—Al9—Al37	58.96 (4)	Al66—Al49—Al48	62.28 (5)
Al8—Al9—Al37	145.41 (7)	Cr15—Al49—Al48	147.04 (7)
Al34^iv^—Al9—Al37	58.81 (5)	Al4—Al49—Al48	147.04 (7)
Al3^iv^—Al9—Al37	58.69 (5)	Cr14—Al49—Al61	55.71 (4)
Al15^iv^—Al9—Cr4	53.98 (4)	Al52—Al49—Al61	148.36 (7)
Cr10—Al9—Cr4	103.13 (5)	Cr1—Al49—Al61	114.78 (6)
Al8—Al9—Cr4	110.78 (6)	Al66—Al49—Al61	91.55 (6)
Al34^iv^—Al9—Cr4	51.38 (4)	Cr15—Al49—Al61	113.75 (6)
Al3^iv^—Al9—Cr4	116.25 (6)	Al4—Al49—Al61	113.75 (6)
Al37—Al9—Cr4	59.03 (4)	Al48—Al49—Al61	59.32 (5)
Al15^iv^—Al9—Al46	101.55 (6)	Cr14—Al49—Al33	61.87 (5)
Cr10—Al9—Al46	53.84 (4)	Al52—Al49—Al33	94.26 (6)
Al8—Al9—Al46	92.10 (6)	Cr1—Al49—Al33	121.95 (6)
Al34^iv^—Al9—Al46	142.29 (7)	Al66—Al49—Al33	149.20 (7)
Al3^iv^—Al9—Al46	59.67 (5)	Cr15—Al49—Al33	62.20 (5)
Al37—Al9—Al46	106.87 (6)	Al4—Al49—Al33	62.20 (5)
Cr4—Al9—Al46	155.27 (7)	Al48—Al49—Al33	90.27 (6)
Al15^iv^—Al9—Al7	95.63 (6)	Al61—Al49—Al33	60.69 (5)
Cr10—Al9—Al7	151.60 (7)	Cr14—Al49—Al57^i^	55.02 (4)
Al8—Al9—Al7	60.73 (5)	Al52—Al49—Al57^i^	150.10 (8)
Al34^iv^—Al9—Al7	58.79 (5)	Cr1—Al49—Al57^i^	115.16 (6)
Al3^iv^—Al9—Al7	142.45 (7)	Al66—Al49—Al57^i^	60.24 (5)
Al37—Al9—Al7	104.69 (6)	Al4—Al49—Al57^i^	114.17 (6)
Cr4—Al9—Al7	50.54 (4)	Al48—Al49—Al57^i^	91.39 (6)
Al46—Al9—Al7	148.44 (7)	Al61—Al49—Al57^i^	61.53 (5)
Al15^iv^—Al9—Al18^xv^	145.78 (7)	Al33—Al49—Al57^i^	110.42 (6)
Cr10—Al9—Al18^xv^	109.53 (6)	Cr14—Al49—Al65	61.90 (5)
Al8—Al9—Al18^xv^	62.46 (5)	Al52—Al49—Al65	95.36 (6)
Al34^iv^—Al9—Al18^xv^	58.53 (5)	Cr1—Al49—Al65	121.44 (6)
Al3^iv^—Al9—Al18^xv^	57.15 (5)	Al66—Al49—Al65	90.12 (6)
Al37—Al9—Al18^xv^	88.78 (6)	Cr15—Al49—Al65	63.67 (5)
Cr4—Al9—Al18^xv^	109.84 (6)	Al4—Al49—Al65	63.67 (5)
Al46—Al9—Al18^xv^	88.64 (6)	Al48—Al49—Al65	148.83 (7)
Al7—Al9—Al18^xv^	91.85 (6)	Al61—Al49—Al65	111.10 (6)
Al15^iv^—Al9—Al64	57.70 (5)	Al33—Al49—Al65	111.30 (6)
Cr10—Al9—Al64	56.87 (4)	Al57^i^—Al49—Al65	60.62 (5)
Al8—Al9—Al64	109.30 (6)	Cr14—Al49—Al51	118.02 (6)
Al34^iv^—Al9—Al64	154.78 (7)	Al52—Al49—Al51	63.60 (5)
Al3^iv^—Al9—Al64	103.39 (6)	Cr1—Al49—Al51	65.04 (4)
Al37—Al9—Al64	105.29 (6)	Al66—Al49—Al51	111.76 (6)
Cr4—Al9—Al64	104.32 (6)	Cr15—Al49—Al51	89.98 (6)
Al46—Al9—Al64	57.36 (5)	Al4—Al49—Al51	89.98 (6)
Al7—Al9—Al64	113.82 (6)	Al48—Al49—Al51	58.39 (5)
Al18^xv^—Al9—Al64	145.54 (7)	Al61—Al49—Al51	85.59 (6)
Cr11^x^—Al10—Cr10^iv^	167.37 (7)	Al33—Al49—Al51	56.96 (5)
Cr11^x^—Al10—Al22^x^	65.14 (5)	Al57^i^—Al49—Al51	144.57 (7)
Cr10^iv^—Al10—Al22^x^	127.13 (7)	Al65—Al49—Al51	152.56 (7)
Cr11^x^—Al10—Al21^iv^	127.63 (7)	Al56—Al50—Al28^i^	124.71 (7)
Cr10^iv^—Al10—Al21^iv^	64.64 (5)	Al56—Al50—Al31	125.05 (7)
Al22^x^—Al10—Al21^iv^	62.51 (5)	Al28^i^—Al50—Al31	66.47 (5)
Cr11^x^—Al10—Al21^x^	63.10 (5)	Al56—Al50—Al63^i^	62.59 (5)
Cr10^iv^—Al10—Al21^x^	123.86 (6)	Al28^i^—Al50—Al63^i^	69.31 (6)
Al22^x^—Al10—Al21^x^	59.11 (5)	Al31—Al50—Al63^i^	125.93 (7)
Al21^iv^—Al10—Al21^x^	90.93 (6)	Al56—Al50—Al65	63.55 (5)
Cr11^x^—Al10—Al22^iv^	124.40 (6)	Al28^i^—Al50—Al65	124.69 (7)
Cr10^iv^—Al10—Al22^iv^	62.54 (5)	Al31—Al50—Al65	68.35 (5)
Al22^x^—Al10—Al22^iv^	90.97 (6)	Al63^i^—Al50—Al65	117.92 (7)
Al21^iv^—Al10—Al22^iv^	58.93 (5)	Al56—Al50—Al48^i^	141.59 (7)
Al21^x^—Al10—Al22^iv^	61.60 (5)	Al28^i^—Al50—Al48^i^	60.86 (5)
Cr11^x^—Al10—Al46^iv^	114.63 (6)	Al31—Al50—Al48^i^	92.85 (6)
Cr10^iv^—Al10—Al46^iv^	56.90 (4)	Al63^i^—Al50—Al48^i^	91.61 (6)
Al22^x^—Al10—Al46^iv^	150.49 (7)	Al65—Al50—Al48^i^	150.34 (7)
Al21^iv^—Al10—Al46^iv^	111.42 (6)	Al56—Al50—Al66	58.31 (5)
Al21^x^—Al10—Al46^iv^	93.52 (6)	Al28^i^—Al50—Al66	142.89 (7)
Al22^iv^—Al10—Al46^iv^	64.21 (5)	Al31—Al50—Al66	147.24 (7)
Cr11^x^—Al10—Al47^x^	56.87 (4)	Al63^i^—Al50—Al66	85.83 (6)
Cr10^iv^—Al10—Al47^x^	114.67 (6)	Al65—Al50—Al66	90.94 (6)
Al22^x^—Al10—Al47^x^	111.73 (6)	Al48^i^—Al50—Al66	94.12 (6)
Al21^iv^—Al10—Al47^x^	150.58 (7)	Al56—Al50—Al52	141.80 (7)
Al21^x^—Al10—Al47^x^	64.43 (5)	Al28^i^—Al50—Al52	92.96 (6)
Al22^iv^—Al10—Al47^x^	93.70 (6)	Al31—Al50—Al52	60.42 (5)
Al46^iv^—Al10—Al47^x^	57.98 (5)	Al63^i^—Al50—Al52	150.74 (7)
Cr11^x^—Al10—Al3	114.02 (6)	Al65—Al50—Al52	91.25 (6)
Cr10^iv^—Al10—Al3	54.30 (4)	Al48^i^—Al50—Al52	59.14 (5)
Al22^x^—Al10—Al3	149.22 (7)	Al66—Al50—Al52	96.60 (6)
Al21^iv^—Al10—Al3	109.76 (6)	Al56—Al50—Al49	88.77 (6)
Al21^x^—Al10—Al3	150.53 (7)	Al28^i^—Al50—Al49	145.84 (7)
Al22^iv^—Al10—Al3	110.85 (6)	Al31—Al50—Al49	89.79 (6)
Al46^iv^—Al10—Al3	59.91 (5)	Al63^i^—Al50—Al49	142.47 (7)
Al47^x^—Al10—Al3	89.00 (6)	Al65—Al50—Al49	60.47 (5)
Cr11^x^—Al10—Al41^iv^	119.97 (6)	Al48^i^—Al50—Al49	98.32 (6)
Cr10^iv^—Al10—Al41^iv^	60.70 (4)	Al66—Al50—Al49	57.54 (5)
Al22^x^—Al10—Al41^iv^	92.47 (6)	Al52—Al50—Al49	53.13 (5)
Al21^iv^—Al10—Al41^iv^	61.75 (5)	Al55—Al51—Al29	124.58 (7)
Al21^x^—Al10—Al41^iv^	148.50 (7)	Al55—Al51—Al33	125.26 (7)
Al22^iv^—Al10—Al41^iv^	109.91 (6)	Al29—Al51—Al33	66.93 (5)
Al46^iv^—Al10—Al41^iv^	110.30 (6)	Al55—Al51—Al64	63.63 (5)
Al47^x^—Al10—Al41^iv^	146.05 (7)	Al29—Al51—Al64	125.14 (7)
Al3—Al10—Al41^iv^	60.33 (5)	Al33—Al51—Al64	68.44 (5)
Cr11^x^—Al10—Al25^x^	60.54 (4)	Al55—Al51—Al63	62.50 (5)
Cr10^iv^—Al10—Al25^x^	120.14 (6)	Al29—Al51—Al63	68.98 (6)
Al22^x^—Al10—Al25^x^	61.87 (5)	Al33—Al51—Al63	125.98 (7)
Al21^iv^—Al10—Al25^x^	92.70 (6)	Al64—Al51—Al63	117.86 (7)
Al21^x^—Al10—Al25^x^	110.14 (6)	Al55—Al51—Al48	141.43 (7)
Al22^iv^—Al10—Al25^x^	148.62 (7)	Al29—Al51—Al48	60.58 (5)
Al46^iv^—Al10—Al25^x^	146.09 (7)	Al33—Al51—Al48	92.79 (6)
Al47^x^—Al10—Al25^x^	110.22 (6)	Al64—Al51—Al48	150.43 (7)
Al3—Al10—Al25^x^	90.21 (6)	Al63—Al51—Al48	91.58 (6)
Al41^iv^—Al10—Al25^x^	59.83 (5)	Al55—Al51—Al66^i^	58.29 (5)
Cr11^x^—Al10—Al6^xiii^	53.93 (4)	Al29—Al51—Al66^i^	142.46 (7)
Cr10^iv^—Al10—Al6^xiii^	114.38 (6)	Al33—Al51—Al66^i^	147.13 (7)
Al22^x^—Al10—Al6^xiii^	109.90 (6)	Al64—Al51—Al66^i^	90.89 (6)
Al21^iv^—Al10—Al6^xiii^	149.23 (7)	Al63—Al51—Al66^i^	85.91 (6)
Al21^x^—Al10—Al6^xiii^	111.08 (6)	Al48—Al51—Al66^i^	94.11 (6)
Al22^iv^—Al10—Al6^xiii^	150.60 (7)	Al55—Al51—Al49	141.92 (7)
Al46^iv^—Al10—Al6^xiii^	89.17 (6)	Al29—Al51—Al49	93.01 (6)
Al47^x^—Al10—Al6^xiii^	59.86 (5)	Al33—Al51—Al49	60.08 (5)
Al3—Al10—Al6^xiii^	60.14 (5)	Al64—Al51—Al49	91.18 (6)
Al41^iv^—Al10—Al6^xiii^	90.23 (6)	Al63—Al51—Al49	150.85 (7)
Al25^x^—Al10—Al6^xiii^	60.20 (5)	Al48—Al51—Al49	59.29 (5)
Cr12—Al11—Cr14^iv^	179.12 (7)	Al66^i^—Al51—Al49	96.74 (6)
Cr12—Al11—Al15^iv^	118.05 (6)	Al55—Al51—Al52	88.79 (6)
Cr14^iv^—Al11—Al15^iv^	61.36 (5)	Al29—Al51—Al52	146.04 (7)
Cr12—Al11—Al13^iv^	61.41 (5)	Al33—Al51—Al52	89.67 (6)
Cr14^iv^—Al11—Al13^iv^	118.15 (6)	Al64—Al51—Al52	60.40 (5)
Al15^iv^—Al11—Al13^iv^	118.94 (7)	Al63—Al51—Al52	142.53 (7)
Cr12—Al11—Cr11	63.06 (4)	Al48—Al51—Al52	98.42 (6)
Cr14^iv^—Al11—Cr11	117.50 (6)	Al66^i^—Al51—Al52	57.54 (5)
Al15^iv^—Al11—Cr11	178.10 (7)	Al49—Al51—Al52	53.22 (5)
Al13^iv^—Al11—Cr11	59.97 (5)	Cr12—Al52—Al49	131.46 (7)
Cr12—Al11—Cr10	117.46 (6)	Cr12—Al52—Cr1	168.39 (7)
Cr14^iv^—Al11—Cr10	62.95 (4)	Al49—Al52—Cr1	60.15 (4)
Al15^iv^—Al11—Cr10	60.21 (5)	Cr12—Al52—Al66^i^	115.50 (6)
Al13^iv^—Al11—Cr10	178.31 (7)	Al49—Al52—Al66^i^	106.00 (6)
Cr11—Al11—Cr10	120.92 (6)	Cr1—Al52—Al66^i^	55.16 (4)
Cr12—Al11—Al22	64.67 (5)	Cr12—Al52—Cr15	69.22 (5)
Cr14^iv^—Al11—Al22	116.14 (6)	Al49—Al52—Cr15	62.24 (5)
Al15^iv^—Al11—Al22	115.69 (7)	Cr1—Al52—Cr15	122.39 (6)
Al13^iv^—Al11—Al22	115.71 (7)	Al66^i^—Al52—Cr15	148.46 (7)
Cr11—Al11—Al22	66.10 (5)	Cr12—Al52—Al4	69.22 (5)
Cr10—Al11—Al22	64.17 (5)	Al49—Al52—Al4	62.24 (5)
Cr12—Al11—Al21	116.50 (6)	Cr1—Al52—Al4	122.39 (6)
Cr14^iv^—Al11—Al21	64.36 (5)	Al66^i^—Al52—Al4	148.46 (7)
Al15^iv^—Al11—Al21	115.41 (7)	Cr12—Al52—Al48^i^	115.04 (6)
Al13^iv^—Al11—Al21	115.89 (7)	Al49—Al52—Al48^i^	105.66 (6)
Cr11—Al11—Al21	64.61 (5)	Cr1—Al52—Al48^i^	55.71 (4)
Cr10—Al11—Al21	65.66 (5)	Al66^i^—Al52—Al48^i^	62.41 (5)
Al22—Al11—Al21	62.96 (5)	Al4—Al52—Al48^i^	146.76 (7)
Cr12—Al11—Al64	64.03 (5)	Cr12—Al52—Al62	55.62 (4)
Cr14^iv^—Al11—Al64	115.86 (6)	Al49—Al52—Al62	148.32 (7)
Al15^iv^—Al11—Al64	63.64 (5)	Cr1—Al52—Al62	115.09 (6)
Al13^iv^—Al11—Al64	115.68 (7)	Al66^i^—Al52—Al62	91.84 (6)
Cr11—Al11—Al64	118.15 (6)	Cr15—Al52—Al62	113.25 (6)
Cr10—Al11—Al64	62.68 (5)	Al4—Al52—Al62	113.25 (6)
Al22—Al11—Al64	63.93 (5)	Al48^i^—Al52—Al62	59.53 (5)
Al21—Al11—Al64	117.21 (7)	Cr12—Al52—Al54	55.25 (4)
Cr12—Al11—Al65^iv^	116.00 (6)	Al49—Al52—Al54	150.11 (8)
Cr14^iv^—Al11—Al65^iv^	64.08 (5)	Cr1—Al52—Al54	115.33 (6)
Al15^iv^—Al11—Al65^iv^	115.54 (7)	Al66^i^—Al52—Al54	60.34 (5)
Al13^iv^—Al11—Al65^iv^	63.58 (5)	Cr15—Al52—Al54	114.12 (6)
Cr11—Al11—Al65^iv^	62.65 (5)	Al4—Al52—Al54	114.12 (6)
Cr10—Al11—Al65^iv^	118.06 (6)	Al48^i^—Al52—Al54	91.51 (6)
Al22—Al11—Al65^iv^	117.40 (7)	Al62—Al52—Al54	61.57 (5)
Al21—Al11—Al65^iv^	64.10 (5)	Cr12—Al52—Al31	61.64 (5)
Al64—Al11—Al65^iv^	178.61 (7)	Al49—Al52—Al31	94.21 (6)
Cr12—Al11—Al4	66.30 (4)	Cr1—Al52—Al31	121.90 (6)
Cr14^iv^—Al11—Al4	112.84 (6)	Al66^i^—Al52—Al31	149.18 (7)
Al15^iv^—Al11—Al4	62.87 (5)	Cr15—Al52—Al31	62.00 (5)
Al13^iv^—Al11—Al4	64.26 (5)	Al4—Al52—Al31	62.00 (5)
Cr11—Al11—Al4	117.03 (6)	Al48^i^—Al52—Al31	90.13 (6)
Cr10—Al11—Al4	114.24 (6)	Al62—Al52—Al31	60.42 (5)
Al22—Al11—Al4	119.81 (6)	Al54—Al52—Al31	110.34 (6)
Al21—Al11—Al4	177.06 (7)	Cr12—Al52—Al64	61.84 (5)
Al64—Al11—Al4	64.53 (5)	Al49—Al52—Al64	95.30 (6)
Al65^iv^—Al11—Al4	114.14 (6)	Cr1—Al52—Al64	121.54 (6)
Cr12—Al11—Al4^iv^	112.75 (6)	Al66^i^—Al52—Al64	90.19 (6)
Cr14^iv^—Al11—Al4^iv^	66.44 (5)	Cr15—Al52—Al64	63.77 (5)
Al15^iv^—Al11—Al4^iv^	64.22 (5)	Al4—Al52—Al64	63.77 (5)
Al13^iv^—Al11—Al4^iv^	62.84 (5)	Al48^i^—Al52—Al64	148.97 (7)
Cr11—Al11—Al4^iv^	114.02 (6)	Al62—Al52—Al64	110.92 (6)
Cr10—Al11—Al4^iv^	117.20 (6)	Al54—Al52—Al64	60.58 (5)
Al22—Al11—Al4^iv^	177.20 (7)	Al31—Al52—Al64	111.23 (6)
Al21—Al11—Al4^iv^	119.75 (6)	Cr12—Al52—Al50	117.75 (6)
Al64—Al11—Al4^iv^	114.22 (6)	Al49—Al52—Al50	63.69 (5)
Al65^iv^—Al11—Al4^iv^	64.43 (5)	Cr1—Al52—Al50	65.07 (4)
Al4—Al11—Al4^iv^	57.49 (5)	Al66^i^—Al52—Al50	111.83 (6)
Cr12—Cr17—Cr14^iv^	179.12 (7)	Cr15—Al52—Al50	89.81 (6)
Cr12—Cr17—Cr11	63.06 (4)	Al4—Al52—Al50	89.81 (6)
Cr14^iv^—Cr17—Cr11	117.50 (6)	Al48^i^—Al52—Al50	58.33 (5)
Cr12—Cr17—Cr10	117.46 (6)	Al62—Al52—Al50	85.56 (6)
Cr14^iv^—Cr17—Cr10	62.95 (4)	Al54—Al52—Al50	144.57 (7)
Cr11—Cr17—Cr10	120.92 (6)	Al31—Al52—Al50	56.87 (5)
Cr12—Cr17—Al22	64.67 (5)	Al64—Al52—Al50	152.50 (7)
Cr14^iv^—Cr17—Al22	116.14 (6)	Cr13—Al53—Cr2^i^	167.98 (7)
Cr11—Cr17—Al22	66.10 (5)	Cr13—Al53—Al59	125.55 (6)
Cr10—Cr17—Al22	64.17 (5)	Cr2^i^—Al53—Al59	63.89 (5)
Cr12—Cr17—Al21	116.50 (6)	Cr13—Al53—Al59^xi^	125.69 (6)
Cr14^iv^—Cr17—Al21	64.36 (5)	Cr2^i^—Al53—Al59^xi^	63.90 (5)
Cr11—Cr17—Al21	64.61 (5)	Al59—Al53—Al59^xi^	63.09 (6)
Cr10—Cr17—Al21	65.66 (5)	Cr13—Al53—Al38	62.38 (5)
Al22—Cr17—Al21	62.96 (5)	Cr2^i^—Al53—Al38	126.93 (6)
Cr12—Cr17—Al64	64.03 (5)	Al59—Al53—Al38	63.27 (5)
Cr14^iv^—Cr17—Al64	115.86 (6)	Al59^xi^—Al53—Al38	95.83 (6)
Cr11—Cr17—Al64	118.15 (6)	Cr13—Al53—Al40	62.26 (5)
Cr10—Cr17—Al64	62.68 (5)	Cr2^i^—Al53—Al40	127.19 (6)
Al22—Cr17—Al64	63.93 (5)	Al59—Al53—Al40	95.98 (6)
Al21—Cr17—Al64	117.21 (7)	Al59^xi^—Al53—Al40	63.51 (5)
Cr12—Cr17—Al65^iv^	116.00 (6)	Al38—Al53—Al40	63.53 (5)
Cr14^iv^—Cr17—Al65^iv^	64.08 (5)	Cr13—Al53—Al54^i^	118.65 (6)
Cr11—Cr17—Al65^iv^	62.65 (5)	Cr2^i^—Al53—Al54^i^	57.48 (4)
Cr10—Cr17—Al65^iv^	118.06 (6)	Al59—Al53—Al54^i^	110.86 (6)
Al22—Cr17—Al65^iv^	117.40 (7)	Al59^xi^—Al53—Al54^i^	60.50 (5)
Al21—Cr17—Al65^iv^	64.10 (5)	Al38—Al53—Al54^i^	152.77 (7)
Al64—Cr17—Al65^iv^	178.61 (7)	Al40—Al53—Al54^i^	92.10 (6)
Cr12—Cr17—Cr15	66.30 (4)	Cr13—Al53—Al57^i^	118.49 (6)
Cr14^iv^—Cr17—Cr15	112.84 (6)	Cr2^i^—Al53—Al57^i^	57.39 (4)
Cr11—Cr17—Cr15	117.03 (6)	Al59—Al53—Al57^i^	60.57 (5)
Cr10—Cr17—Cr15	114.24 (6)	Al59^xi^—Al53—Al57^i^	110.85 (6)
Al22—Cr17—Cr15	119.81 (6)	Al38—Al53—Al57^i^	92.08 (6)
Al21—Cr17—Cr15	177.06 (7)	Al40—Al53—Al57^i^	152.92 (7)
Al64—Cr17—Cr15	64.53 (5)	Al54^i^—Al53—Al57^i^	108.27 (6)
Al65^iv^—Cr17—Cr15	114.14 (6)	Cr13—Al53—Al61	56.63 (4)
Cr9—Al12—Cr9^xi^	172.13 (10)	Cr2^i^—Al53—Al61	118.98 (6)
Cr9—Al12—Al40^xi^	124.32 (6)	Al59—Al53—Al61	93.24 (6)
Cr9^xi^—Al12—Al40^xi^	61.87 (4)	Al59^xi^—Al53—Al61	153.10 (7)
Cr9—Al12—Al40	61.87 (4)	Al38—Al53—Al61	60.19 (5)
Cr9^xi^—Al12—Al40	124.32 (6)	Al40—Al53—Al61	109.93 (6)
Al40^xi^—Al12—Al40	95.38 (9)	Al54^i^—Al53—Al61	145.64 (7)
Cr9—Al12—Al38	61.75 (4)	Al57^i^—Al53—Al61	62.07 (5)
Cr9^xi^—Al12—Al38	124.47 (6)	Cr13—Al53—Al62^i^	56.85 (4)
Al40^xi^—Al12—Al38	62.65 (5)	Cr2^i^—Al53—Al62^i^	119.04 (6)
Al40—Al12—Al38	63.28 (5)	Al59—Al53—Al62^i^	152.91 (7)
Cr9—Al12—Al38^xi^	124.47 (6)	Al59^xi^—Al53—Al62^i^	93.09 (6)
Cr9^xi^—Al12—Al38^xi^	61.75 (4)	Al38—Al53—Al62^i^	110.03 (6)
Al40^xi^—Al12—Al38^xi^	63.28 (5)	Al40—Al53—Al62^i^	59.90 (5)
Al40—Al12—Al38^xi^	62.65 (5)	Al54^i^—Al53—Al62^i^	62.03 (5)
Al38—Al12—Al38^xi^	95.04 (9)	Al57^i^—Al53—Al62^i^	145.76 (7)
Cr9—Al12—Al23^vi^	120.23 (4)	Al61—Al53—Al62^i^	106.04 (6)
Cr9^xi^—Al12—Al23^vi^	56.98 (3)	Cr13—Al53—Al66	113.83 (6)
Al40^xi^—Al12—Al23^vi^	60.72 (4)	Cr2^i^—Al53—Al66	54.15 (4)
Al40—Al12—Al23^vi^	153.48 (8)	Al59—Al53—Al66	109.53 (6)
Al38—Al12—Al23^vi^	93.54 (4)	Al59^xi^—Al53—Al66	109.48 (6)
Al38^xi^—Al12—Al23^vi^	110.20 (5)	Al38—Al53—Al66	147.08 (7)
Cr9—Al12—Al23^ix^	56.98 (3)	Al40—Al53—Al66	147.00 (7)
Cr9^xi^—Al12—Al23^ix^	120.23 (4)	Al54^i^—Al53—Al66	59.83 (5)
Al40^xi^—Al12—Al23^ix^	153.48 (8)	Al57^i^—Al53—Al66	59.80 (5)
Al40—Al12—Al23^ix^	60.72 (4)	Al61—Al53—Al66	89.70 (6)
Al38—Al12—Al23^ix^	110.20 (5)	Al62^i^—Al53—Al66	89.80 (6)
Al38^xi^—Al12—Al23^ix^	93.54 (4)	Cr13—Al53—Al48	53.55 (4)
Al23^vi^—Al12—Al23^ix^	144.92 (10)	Cr2^i^—Al53—Al48	114.42 (6)
Cr9—Al12—Al20^xi^	120.51 (4)	Al59—Al53—Al48	147.88 (6)
Cr9^xi^—Al12—Al20^xi^	56.69 (3)	Al59^xi^—Al53—Al48	147.78 (6)
Al40^xi^—Al12—Al20^xi^	110.24 (5)	Al38—Al53—Al48	107.41 (6)
Al40—Al12—Al20^xi^	93.44 (4)	Al40—Al53—Al48	107.13 (6)
Al38—Al12—Al20^xi^	153.31 (8)	Al54^i^—Al53—Al48	90.66 (6)
Al38^xi^—Al12—Al20^xi^	60.90 (4)	Al57^i^—Al53—Al48	90.70 (6)
Al23^vi^—Al12—Al20^xi^	105.17 (5)	Al61—Al53—Al48	58.32 (5)
Al23^ix^—Al12—Al20^xi^	63.68 (4)	Al62^i^—Al53—Al48	58.40 (5)
Cr9—Al12—Al20	56.69 (3)	Al66—Al53—Al48	60.28 (5)
Cr9^xi^—Al12—Al20	120.51 (5)	Cr12—Al54—Cr2	166.17 (7)
Al40^xi^—Al12—Al20	93.44 (4)	Cr12—Al54—Al58^iii^	130.27 (6)
Al40—Al12—Al20	110.24 (5)	Cr2—Al54—Al58^iii^	63.39 (5)
Al38—Al12—Al20	60.91 (4)	Cr12—Al54—Al22	64.01 (5)
Al38^xi^—Al12—Al20	153.31 (8)	Cr2—Al54—Al22	129.59 (6)
Al23^vi^—Al12—Al20	63.68 (4)	Al58^iii^—Al54—Al22	66.26 (5)
Al23^ix^—Al12—Al20	105.17 (5)	Cr12—Al54—Al55	121.95 (6)
Al20^xi^—Al12—Al20	144.90 (10)	Cr2—Al54—Al55	59.51 (4)
Cr9—Al12—Al34^ix^	117.55 (8)	Al58^iii^—Al54—Al55	61.68 (5)
Cr9^xi^—Al12—Al34^ix^	54.58 (4)	Al22—Al54—Al55	94.46 (6)
Al40^xi^—Al12—Al34^ix^	107.48 (4)	Cr12—Al54—Al53^i^	114.31 (6)
Al40—Al12—Al34^ix^	147.95 (5)	Cr2—Al54—Al53^i^	55.81 (4)
Al38—Al12—Al34^ix^	147.71 (5)	Al58^iii^—Al54—Al53^i^	108.90 (6)
Al38^xi^—Al12—Al34^ix^	107.84 (4)	Al22—Al54—Al53^i^	150.98 (7)
Al23^vi^—Al12—Al34^ix^	57.63 (5)	Al55—Al54—Al53^i^	108.46 (6)
Al23^ix^—Al12—Al34^ix^	91.23 (6)	Cr12—Al54—Al59^iii^	122.78 (6)
Al20^xi^—Al12—Al34^ix^	58.01 (5)	Cr2—Al54—Al59^iii^	62.71 (5)
Al20—Al12—Al34^ix^	90.89 (6)	Al58^iii^—Al54—Al59^iii^	62.84 (5)
Cr9—Al12—Al34^vi^	54.58 (4)	Al22—Al54—Al59^iii^	96.31 (6)
Cr9^xi^—Al12—Al34^vi^	117.55 (8)	Al55—Al54—Al59^iii^	112.20 (6)
Al40^xi^—Al12—Al34^vi^	147.95 (5)	Al53^i^—Al54—Al59^iii^	59.03 (5)
Al40—Al12—Al34^vi^	107.48 (4)	Cr12—Al54—Al66^i^	113.36 (6)
Al38—Al12—Al34^vi^	107.84 (4)	Cr2—Al54—Al66^i^	54.41 (4)
Al38^xi^—Al12—Al34^vi^	147.71 (5)	Al58^iii^—Al54—Al66^i^	108.00 (6)
Al23^vi^—Al12—Al34^vi^	91.23 (6)	Al22—Al54—Al66^i^	147.25 (7)
Al23^ix^—Al12—Al34^vi^	57.63 (5)	Al55—Al54—Al66^i^	57.90 (5)
Al20^xi^—Al12—Al34^vi^	90.89 (6)	Al53^i^—Al54—Al66^i^	61.63 (5)
Al20—Al12—Al34^vi^	58.01 (5)	Al59^iii^—Al54—Al66^i^	109.75 (6)
Al34^ix^—Al12—Al34^vi^	62.98 (7)	Cr12—Al54—Al52	54.49 (4)
Al11^iv^—Al13—Cr11^iv^	60.32 (5)	Cr2—Al54—Al52	113.23 (6)
Al11^iv^—Al13—Cr3	177.37 (7)	Al58^iii^—Al54—Al52	148.81 (7)
Cr11^iv^—Al13—Cr3	119.35 (6)	Al22—Al54—Al52	108.19 (6)
Al11^iv^—Al13—Al13^iv^	120.39 (4)	Al55—Al54—Al52	89.28 (6)
Cr11^iv^—Al13—Al13^iv^	178.72 (8)	Al53^i^—Al54—Al52	90.09 (6)
Cr3—Al13—Al13^iv^	59.89 (3)	Al59^iii^—Al54—Al52	146.16 (7)
Al11^iv^—Al13—Cr12^iv^	58.87 (5)	Al66^i^—Al54—Al52	58.95 (5)
Cr11^iv^—Al13—Cr12^iv^	62.30 (4)	Cr12—Al54—Al25	60.20 (4)
Cr3—Al13—Cr12^iv^	118.54 (5)	Cr2—Al54—Al25	125.36 (6)
Al13^iv^—Al13—Cr12^iv^	116.98 (7)	Al58^iii^—Al54—Al25	96.96 (6)
Al11^iv^—Al13—Al45^iv^	112.46 (6)	Al22—Al54—Al25	62.91 (5)
Cr11^iv^—Al13—Al45^iv^	63.74 (5)	Al55—Al54—Al25	154.76 (7)
Cr3—Al13—Al45^iv^	65.71 (5)	Al53^i^—Al54—Al25	90.50 (6)
Al13^iv^—Al13—Al45^iv^	115.03 (6)	Al59^iii^—Al54—Al25	63.05 (5)
Cr12^iv^—Al13—Al45^iv^	63.52 (5)	Al66^i^—Al54—Al25	147.11 (7)
Al11^iv^—Al13—Al31^iv^	112.54 (7)	Al52—Al54—Al25	107.68 (6)
Cr11^iv^—Al13—Al31^iv^	116.01 (6)	Cr12—Al54—Al64	61.43 (5)
Cr3—Al13—Al31^iv^	65.07 (4)	Cr2—Al54—Al64	120.42 (6)
Al13^iv^—Al13—Al31^iv^	62.80 (6)	Al58^iii^—Al54—Al64	93.63 (6)
Cr12^iv^—Al13—Al31^iv^	62.98 (5)	Al22—Al54—Al64	60.18 (5)
Al45^iv^—Al13—Al31^iv^	63.95 (5)	Al55—Al54—Al64	61.22 (5)
Al11^iv^—Al13—Al44	114.70 (6)	Al53^i^—Al54—Al64	147.13 (7)
Cr11^iv^—Al13—Al44	62.68 (5)	Al59^iii^—Al54—Al64	153.04 (7)
Cr3—Al13—Al44	66.48 (5)	Al66^i^—Al54—Al64	89.17 (6)
Al13^iv^—Al13—Al44	117.29 (6)	Al52—Al54—Al64	60.19 (5)
Cr12^iv^—Al13—Al44	115.64 (6)	Al25—Al54—Al64	110.71 (6)
Al45^iv^—Al13—Al44	64.01 (5)	Cr12—Al54—Al62	54.46 (4)
Al31^iv^—Al13—Al44	119.20 (7)	Cr2—Al54—Al62	115.32 (6)
Al11^iv^—Al13—Al31	118.44 (7)	Al58^iii^—Al54—Al62	151.91 (7)
Cr11^iv^—Al13—Al31	120.59 (6)	Al22—Al54—Al62	109.93 (6)
Cr3—Al13—Al31	64.11 (4)	Al55—Al54—Al62	144.45 (7)
Al13^iv^—Al13—Al31	60.21 (6)	Al53^i^—Al54—Al62	59.94 (5)
Cr12^iv^—Al13—Al31	175.16 (7)	Al59^iii^—Al54—Al62	90.99 (6)
Al45^iv^—Al13—Al31	121.00 (7)	Al66^i^—Al54—Al62	89.64 (5)
Al31^iv^—Al13—Al31	116.65 (6)	Al52—Al54—Al62	59.19 (5)
Al44—Al13—Al31	68.96 (5)	Al25—Al54—Al62	59.73 (5)
Al11^iv^—Al13—Al65	60.31 (5)	Al64—Al54—Al62	108.68 (6)
Cr11^iv^—Al13—Al65	61.60 (4)	Al43—Al55—Cr2	124.55 (6)
Cr3—Al13—Al65	122.08 (6)	Al43—Al55—Al46	63.68 (5)
Al13^iv^—Al13—Al65	119.64 (8)	Cr2—Al55—Al46	123.52 (6)
Cr12^iv^—Al13—Al65	111.08 (6)	Al43—Al55—Al51	107.14 (7)
Al45^iv^—Al13—Al65	117.93 (6)	Cr2—Al55—Al51	118.66 (6)
Al31^iv^—Al13—Al65	172.84 (7)	Al46—Al55—Al51	106.85 (6)
Al44—Al13—Al65	66.42 (5)	Al43—Al55—Al66^i^	146.17 (7)
Al31—Al13—Al65	68.89 (5)	Cr2—Al55—Al66^i^	54.92 (4)
Al11^iv^—Al13—Al4	65.28 (5)	Al46—Al55—Al66^i^	149.11 (7)
Cr11^iv^—Al13—Al4	116.36 (6)	Al51—Al55—Al66^i^	63.80 (5)
Cr3—Al13—Al4	116.42 (6)	Al43—Al55—Al54	150.52 (7)
Al13^iv^—Al13—Al4	64.84 (4)	Cr2—Al55—Al54	56.27 (4)
Cr12^iv^—Al13—Al4	112.70 (6)	Al46—Al55—Al54	90.87 (6)
Al45^iv^—Al13—Al4	175.94 (7)	Al51—Al55—Al54	93.85 (6)
Al31^iv^—Al13—Al4	113.37 (6)	Al66^i^—Al55—Al54	62.05 (5)
Al44—Al13—Al4	119.87 (6)	Al43—Al55—Al60^i^	64.83 (6)
Al31—Al13—Al4	62.73 (5)	Cr2—Al55—Al60^i^	59.82 (5)
Al65—Al13—Al4	64.32 (5)	Al46—Al55—Al60^i^	95.38 (5)
Al11^iv^—Al13—Al4^iv^	64.32 (5)	Al51—Al55—Al60^i^	150.00 (7)
Cr11^iv^—Al13—Al4^iv^	117.38 (6)	Al66^i^—Al55—Al60^i^	105.73 (6)
Cr3—Al13—Al4^iv^	114.58 (5)	Al54—Al55—Al60^i^	105.93 (7)
Al13^iv^—Al13—Al4^iv^	62.69 (4)	Al43—Al55—Al58^iii^	94.95 (6)
Cr12^iv^—Al13—Al4^iv^	65.58 (4)	Cr2—Al55—Al58^iii^	60.99 (4)
Al45^iv^—Al13—Al4^iv^	117.96 (6)	Al46—Al55—Al58^iii^	62.66 (5)
Al31^iv^—Al13—Al4^iv^	62.73 (5)	Al51—Al55—Al58^iii^	148.20 (7)
Al44—Al13—Al4^iv^	177.96 (7)	Al66^i^—Al55—Al58^iii^	108.63 (6)
Al31—Al13—Al4^iv^	109.77 (6)	Al54—Al55—Al58^iii^	58.31 (5)
Al65—Al13—Al4^iv^	111.71 (6)	Al60^i^—Al55—Al58^iii^	60.64 (5)
Al4—Al13—Al4^iv^	58.15 (6)	Al43—Al55—Al63	62.06 (5)
Cr11^iv^—Cr18—Cr3	119.35 (6)	Cr2—Al55—Al63	116.77 (6)
Cr11^iv^—Cr18—Cr12^iv^	62.30 (4)	Al46—Al55—Al63	114.20 (6)
Cr3—Cr18—Cr12^iv^	118.54 (5)	Al51—Al55—Al63	59.78 (5)
Cr11^iv^—Cr18—Al45^iv^	63.74 (5)	Al66^i^—Al55—Al63	87.36 (6)
Cr3—Cr18—Al45^iv^	65.71 (5)	Al54—Al55—Al63	147.17 (7)
Cr12^iv^—Cr18—Al45^iv^	63.52 (5)	Al60^i^—Al55—Al63	93.02 (6)
Cr11^iv^—Cr18—Al31^iv^	116.01 (6)	Al58^iii^—Al55—Al63	151.68 (7)
Cr3—Cr18—Al31^iv^	65.07 (4)	Al43—Al55—Al56^i^	91.96 (6)
Cr12^iv^—Cr18—Al31^iv^	62.98 (5)	Cr2—Al55—Al56^i^	57.35 (4)
Al45^iv^—Cr18—Al31^iv^	63.95 (5)	Al46—Al55—Al56^i^	151.62 (7)
Cr11^iv^—Cr18—Al44	62.68 (5)	Al51—Al55—Al56^i^	93.66 (6)
Cr3—Cr18—Al44	66.48 (5)	Al66^i^—Al55—Al56^i^	58.13 (5)
Cr12^iv^—Cr18—Al44	115.64 (6)	Al54—Al55—Al56^i^	107.44 (6)
Al45^iv^—Cr18—Al44	64.01 (5)	Al60^i^—Al55—Al56^i^	59.27 (4)
Al31^iv^—Cr18—Al44	119.20 (7)	Al58^iii^—Al55—Al56^i^	108.45 (6)
Cr11^iv^—Cr18—Al31	120.59 (6)	Al63—Al55—Al56^i^	59.71 (5)
Cr3—Cr18—Al31	64.11 (4)	Al43—Al55—Al64	112.27 (6)
Cr12^iv^—Cr18—Al31	175.16 (7)	Cr2—Al55—Al64	117.14 (6)
Al45^iv^—Cr18—Al31	121.00 (7)	Al46—Al55—Al64	60.76 (5)
Al31^iv^—Cr18—Al31	116.65 (6)	Al51—Al55—Al64	59.17 (5)
Al44—Cr18—Al31	68.96 (5)	Al66^i^—Al55—Al64	91.46 (6)
Cr11^iv^—Cr18—Al65	61.60 (4)	Al54—Al55—Al64	61.16 (5)
Cr3—Cr18—Al65	122.08 (6)	Al60^i^—Al55—Al64	150.66 (6)
Cr12^iv^—Cr18—Al65	111.08 (6)	Al58^iii^—Al55—Al64	91.63 (6)
Al45^iv^—Cr18—Al65	117.93 (6)	Al63—Al55—Al64	111.70 (6)
Al31^iv^—Cr18—Al65	172.84 (7)	Al56^i^—Al55—Al64	147.28 (7)
Al44—Cr18—Al65	66.42 (5)	Al42^i^—Al56—Cr2^i^	124.61 (6)
Al31—Cr18—Al65	68.89 (5)	Al42^i^—Al56—Al47^iv^	63.82 (5)
Cr11^iv^—Cr18—Cr15	116.36 (6)	Cr2^i^—Al56—Al47^iv^	123.60 (6)
Cr3—Cr18—Cr15	116.42 (6)	Al42^i^—Al56—Al50	106.99 (7)
Cr12^iv^—Cr18—Cr15	112.70 (6)	Cr2^i^—Al56—Al50	118.62 (6)
Al45^iv^—Cr18—Cr15	175.94 (7)	Al47^iv^—Al56—Al50	106.81 (6)
Al31^iv^—Cr18—Cr15	113.37 (6)	Al42^i^—Al56—Al66	146.08 (7)
Al44—Cr18—Cr15	119.87 (6)	Cr2^i^—Al56—Al66	54.87 (4)
Al31—Cr18—Cr15	62.73 (5)	Al47^iv^—Al56—Al66	149.04 (7)
Al65—Cr18—Cr15	64.32 (5)	Al50—Al56—Al66	63.82 (5)
Cr16—Al14—Al61	103.78 (6)	Al42^i^—Al56—Al57^i^	150.62 (7)
Al5—Al14—Al61	103.78 (6)	Cr2^i^—Al56—Al57^i^	56.19 (4)
Cr16—Al14—Al20	103.06 (6)	Al47^iv^—Al56—Al57^i^	90.86 (6)
Al5—Al14—Al20	103.06 (6)	Al50—Al56—Al57^i^	93.99 (6)
Al61—Al14—Al20	118.13 (7)	Al66—Al56—Al57^i^	62.03 (5)
Cr16—Al14—Al29	59.54 (5)	Al42^i^—Al56—Al60	64.92 (6)
Al5—Al14—Al29	59.54 (5)	Cr2^i^—Al56—Al60	59.80 (5)
Al61—Al14—Al29	62.44 (5)	Al47^iv^—Al56—Al60	95.53 (5)
Al20—Al14—Al29	160.10 (7)	Al50—Al56—Al60	149.89 (7)
Cr16—Al14—Cr9	55.87 (4)	Al66—Al56—Al60	105.67 (6)
Al5—Al14—Cr9	55.87 (4)	Al57^i^—Al56—Al60	105.83 (7)
Al61—Al14—Cr9	102.09 (5)	Al42^i^—Al56—Al58	95.21 (6)
Al20—Al14—Cr9	54.32 (4)	Cr2^i^—Al56—Al58	60.85 (4)
Al29—Al14—Cr9	105.78 (5)	Al47^iv^—Al56—Al58	62.89 (5)
Cr16—Al14—Cr13	55.68 (4)	Al50—Al56—Al58	148.28 (7)
Al5—Al14—Cr13	55.68 (4)	Al66—Al56—Al58	108.44 (6)
Al61—Al14—Cr13	54.64 (4)	Al57^i^—Al56—Al58	58.16 (5)
Al20—Al14—Cr13	102.59 (6)	Al60—Al56—Al58	60.62 (5)
Al29—Al14—Cr13	60.46 (4)	Al42^i^—Al56—Al63^i^	62.07 (5)
Cr9—Al14—Cr13	55.20 (3)	Cr2^i^—Al56—Al63^i^	116.50 (6)
Al5—Al14—Cr7^vi^	56.74 (4)	Al47^iv^—Al56—Al63^i^	114.42 (6)
Al61—Al14—Cr7^vi^	119.34 (6)	Al50—Al56—Al63^i^	59.75 (5)
Al20—Al14—Cr7^vi^	122.09 (6)	Al66—Al56—Al63^i^	87.20 (6)
Al29—Al14—Cr7^vi^	58.72 (4)	Al57^i^—Al56—Al63^i^	147.09 (7)
Cr9—Al14—Cr7^vi^	106.34 (5)	Al60—Al56—Al63^i^	92.91 (6)
Cr13—Al14—Cr7^vi^	104.24 (5)	Al58—Al56—Al63^i^	151.69 (7)
Cr16—Al14—Al19	58.18 (5)	Al42^i^—Al56—Al55^i^	91.99 (6)
Al5—Al14—Al19	58.18 (5)	Cr2^i^—Al56—Al55^i^	57.26 (4)
Al61—Al14—Al19	160.08 (7)	Al47^iv^—Al56—Al55^i^	151.79 (7)
Al20—Al14—Al19	63.60 (5)	Al50—Al56—Al55^i^	93.56 (6)
Al29—Al14—Al19	108.70 (6)	Al66—Al56—Al55^i^	58.04 (5)
Cr9—Al14—Al19	61.75 (4)	Al57^i^—Al56—Al55^i^	107.30 (6)
Cr13—Al14—Al19	105.49 (5)	Al60—Al56—Al55^i^	59.28 (4)
Cr7^vi^—Al14—Al19	60.16 (4)	Al58—Al56—Al55^i^	108.31 (6)
Al5—Al14—Al20^vi^	99.71 (6)	Al63^i^—Al56—Al55^i^	59.53 (5)
Al61—Al14—Al20^vi^	139.84 (7)	Al42^i^—Al56—Al65	112.14 (6)
Al20—Al14—Al20^vi^	86.95 (6)	Cr2^i^—Al56—Al65	117.28 (6)
Al29—Al14—Al20^vi^	104.64 (6)	Al47^iv^—Al56—Al65	60.56 (5)
Cr9—Al14—Al20^vi^	118.05 (6)	Al50—Al56—Al65	59.26 (5)
Cr13—Al14—Al20^vi^	154.86 (6)	Al66—Al56—Al65	91.60 (6)
Cr7^vi^—Al14—Al20^vi^	52.07 (4)	Al57^i^—Al56—Al65	61.37 (5)
Al19—Al14—Al20^vi^	57.78 (5)	Al60—Al56—Al65	150.67 (6)
Cr16—Al14—Al38	102.34 (6)	Al58—Al56—Al65	91.72 (6)
Al5—Al14—Al38	102.34 (6)	Al63^i^—Al56—Al65	111.80 (6)
Al61—Al14—Al38	59.89 (5)	Al55^i^—Al56—Al65	147.31 (7)
Al20—Al14—Al38	60.42 (5)	Cr14^i^—Al57—Cr2	166.10 (7)
Al29—Al14—Al38	111.16 (6)	Cr14^i^—Al57—Al58^i^	130.33 (6)
Cr9—Al14—Al38	56.48 (4)	Cr2—Al57—Al58^i^	63.40 (5)
Cr13—Al14—Al38	56.95 (4)	Cr14^i^—Al57—Al21^xix^	63.96 (5)
Cr7^vi^—Al14—Al38	158.99 (6)	Cr2—Al57—Al21^xix^	129.71 (6)
Al19—Al14—Al38	112.65 (6)	Al58^i^—Al57—Al21^xix^	66.37 (5)
Al20^vi^—Al14—Al38	143.87 (7)	Cr14^i^—Al57—Al56^i^	121.89 (6)
Cr16—Al14—Al7	99.49 (6)	Cr2—Al57—Al56^i^	59.59 (4)
Al5—Al14—Al7	99.49 (6)	Al58^i^—Al57—Al56^i^	61.78 (5)
Al61—Al14—Al7	85.00 (6)	Al21^xix^—Al57—Al56^i^	94.58 (6)
Al20—Al14—Al7	141.95 (7)	Cr14^i^—Al57—Al53^i^	114.27 (6)
Al29—Al14—Al7	56.29 (5)	Cr2—Al57—Al53^i^	55.78 (4)
Cr9—Al14—Al7	155.26 (6)	Al58^i^—Al57—Al53^i^	108.86 (6)
Cr13—Al14—Al7	115.44 (6)	Al21^xix^—Al57—Al53^i^	150.90 (7)
Cr7^vi^—Al14—Al7	50.95 (4)	Al56^i^—Al57—Al53^i^	108.50 (6)
Al19—Al14—Al7	105.20 (6)	Cr14^i^—Al57—Al59^i^	122.83 (6)
Al20^vi^—Al14—Al7	59.10 (5)	Cr2—Al57—Al59^i^	62.64 (5)
Al38—Al14—Al7	142.06 (7)	Al58^i^—Al57—Al59^i^	62.85 (5)
Al11^iv^—Al15—Cr4	177.43 (7)	Al21^xix^—Al57—Al59^i^	96.40 (6)
Al11^iv^—Al15—Cr10^iv^	60.40 (5)	Al56^i^—Al57—Al59^i^	112.27 (6)
Cr4—Al15—Cr10^iv^	119.38 (6)	Al53^i^—Al57—Al59^i^	58.91 (5)
Al11^iv^—Al15—Al15^iv^	120.48 (4)	Cr14^i^—Al57—Al66^i^	113.30 (6)
Cr4—Al15—Al15^iv^	59.69 (3)	Cr2—Al57—Al66^i^	54.41 (4)
Cr10^iv^—Al15—Al15^iv^	178.62 (7)	Al58^i^—Al57—Al66^i^	108.07 (6)
Al11^iv^—Al15—Cr14	59.19 (5)	Al21^xix^—Al57—Al66^i^	147.40 (7)
Cr4—Al15—Cr14	118.30 (5)	Al56^i^—Al57—Al66^i^	57.96 (5)
Cr10^iv^—Al15—Cr14	62.26 (4)	Al53^i^—Al57—Al66^i^	61.58 (5)
Al15^iv^—Al15—Cr14	117.07 (7)	Al59^i^—Al57—Al66^i^	109.63 (6)
Al11^iv^—Al15—Al37^iv^	112.45 (6)	Cr14^i^—Al57—Al41^xix^	60.43 (5)
Cr4—Al15—Al37^iv^	65.84 (5)	Cr2—Al57—Al41^xix^	125.12 (6)
Cr10^iv^—Al15—Al37^iv^	63.50 (5)	Al58^i^—Al57—Al41^xix^	96.81 (6)
Al15^iv^—Al15—Al37^iv^	115.14 (6)	Al21^xix^—Al57—Al41^xix^	62.90 (5)
Cr14—Al15—Al37^iv^	63.29 (5)	Al56^i^—Al57—Al41^xix^	154.78 (7)
Al11^iv^—Al15—Al33	112.65 (7)	Al53^i^—Al57—Al41^xix^	90.35 (6)
Cr4—Al15—Al33	64.97 (4)	Al59^i^—Al57—Al41^xix^	62.87 (5)
Cr10^iv^—Al15—Al33	115.99 (6)	Al66^i^—Al57—Al41^xix^	147.00 (7)
Al15^iv^—Al15—Al33	62.78 (6)	Cr14^i^—Al57—Al49^i^	54.41 (4)
Cr14—Al15—Al33	62.89 (4)	Cr2—Al57—Al49^i^	113.26 (6)
Al37^iv^—Al15—Al33	64.14 (5)	Al58^i^—Al57—Al49^i^	148.90 (7)
Al11^iv^—Al15—Al9^iv^	114.38 (7)	Al21^xix^—Al57—Al49^i^	108.13 (6)
Cr4—Al15—Al9^iv^	66.86 (5)	Al56^i^—Al57—Al49^i^	89.27 (6)
Cr10^iv^—Al15—Al9^iv^	62.45 (5)	Al53^i^—Al57—Al49^i^	90.10 (6)
Al15^iv^—Al15—Al9^iv^	117.40 (6)	Al59^i^—Al57—Al49^i^	146.05 (7)
Cr14—Al15—Al9^iv^	115.41 (6)	Al66^i^—Al57—Al49^i^	58.98 (5)
Al37^iv^—Al15—Al9^iv^	63.99 (5)	Al41^xix^—Al57—Al49^i^	107.79 (6)
Al33—Al15—Al9^iv^	119.43 (7)	Cr14^i^—Al57—Al65^i^	61.52 (5)
Al11^iv^—Al15—Al33^iv^	118.47 (7)	Cr2—Al57—Al65^i^	120.36 (6)
Cr4—Al15—Al33^iv^	64.00 (4)	Al58^i^—Al57—Al65^i^	93.66 (6)
Cr10^iv^—Al15—Al33^iv^	120.54 (6)	Al21^xix^—Al57—Al65^i^	60.28 (5)
Al15^iv^—Al15—Al33^iv^	60.21 (6)	Al56^i^—Al57—Al65^i^	61.09 (5)
Cr14—Al15—Al33^iv^	175.53 (7)	Al53^i^—Al57—Al65^i^	147.10 (7)
Al37^iv^—Al15—Al33^iv^	120.83 (7)	Al59^i^—Al57—Al65^i^	153.16 (7)
Al33—Al15—Al33^iv^	116.60 (6)	Al66^i^—Al57—Al65^i^	89.16 (6)
Al9^iv^—Al15—Al33^iv^	68.88 (5)	Al41^xix^—Al57—Al65^i^	110.99 (6)
Al11^iv^—Al15—Al64^iv^	60.40 (5)	Al49^i^—Al57—Al65^i^	60.20 (5)
Cr4—Al15—Al64^iv^	121.98 (6)	Cr14^i^—Al57—Al61^i^	54.57 (4)
Cr10^iv^—Al15—Al64^iv^	61.59 (4)	Cr2—Al57—Al61^i^	115.12 (6)
Al15^iv^—Al15—Al64^iv^	119.71 (7)	Al58^i^—Al57—Al61^i^	151.90 (7)
Cr14—Al15—Al64^iv^	111.30 (6)	Al21^xix^—Al57—Al61^i^	109.99 (6)
Al37^iv^—Al15—Al64^iv^	117.59 (6)	Al56^i^—Al57—Al61^i^	144.30 (7)
Al33—Al15—Al64^iv^	173.05 (7)	Al53^i^—Al57—Al61^i^	59.78 (5)
Al9^iv^—Al15—Al64^iv^	65.95 (5)	Al59^i^—Al57—Al61^i^	90.91 (6)
Al33^iv^—Al15—Al64^iv^	68.86 (5)	Al66^i^—Al57—Al61^i^	89.41 (6)
Al11^iv^—Al15—Al4^iv^	65.32 (5)	Al41^xix^—Al57—Al61^i^	59.92 (5)
Cr4—Al15—Al4^iv^	116.27 (6)	Al49^i^—Al57—Al61^i^	59.12 (5)
Cr10^iv^—Al15—Al4^iv^	116.45 (6)	Al65^i^—Al57—Al61^i^	108.81 (6)
Al15^iv^—Al15—Al4^iv^	64.88 (4)	Al57^i^—Al58—Al54^x^	117.02 (7)
Cr14—Al15—Al4^iv^	113.09 (6)	Al57^i^—Al58—Cr2^i^	55.50 (4)
Al37^iv^—Al15—Al4^iv^	176.17 (7)	Al54^x^—Al58—Cr2^i^	104.35 (6)
Al33—Al15—Al4^iv^	113.45 (6)	Al57^i^—Al58—Cr2^x^	104.47 (6)
Al9^iv^—Al15—Al4^iv^	119.62 (6)	Al54^x^—Al58—Cr2^x^	55.44 (4)
Al33^iv^—Al15—Al4^iv^	62.74 (5)	Cr2^i^—Al58—Cr2^x^	57.15 (4)
Al64^iv^—Al15—Al4^iv^	64.43 (5)	Al57^i^—Al58—Al55^x^	159.93 (7)
Al11^iv^—Al15—Al4	64.54 (5)	Al54^x^—Al58—Al55^x^	60.01 (5)
Cr4—Al15—Al4	114.30 (5)	Cr2^i^—Al58—Al55^x^	104.87 (6)
Cr10^iv^—Al15—Al4	117.63 (6)	Cr2^x^—Al58—Al55^x^	56.58 (4)
Al15^iv^—Al15—Al4	62.58 (4)	Al57^i^—Al58—Al56	60.06 (5)
Cr14—Al15—Al4	65.93 (4)	Al54^x^—Al58—Al56	159.88 (7)
Al37^iv^—Al15—Al4	118.16 (6)	Cr2^i^—Al58—Al56	56.66 (4)
Al33—Al15—Al4	62.68 (5)	Cr2^x^—Al58—Al56	104.87 (6)
Al9^iv^—Al15—Al4	177.77 (7)	Al55^x^—Al58—Al56	115.03 (6)
Al33^iv^—Al15—Al4	109.74 (6)	Al57^i^—Al58—Al60	106.39 (6)
Al64^iv^—Al15—Al4	112.00 (6)	Al54^x^—Al58—Al60	106.35 (6)
Al4^iv^—Al15—Al4	58.22 (6)	Cr2^i^—Al58—Al60	58.06 (4)
Cr4—Cr19—Cr10^iv^	119.38 (6)	Cr2^x^—Al58—Al60	58.02 (4)
Cr4—Cr19—Cr14	118.30 (5)	Al55^x^—Al58—Al60	59.48 (4)
Cr10^iv^—Cr19—Cr14	62.26 (4)	Al56—Al58—Al60	59.45 (4)
Cr4—Cr19—Al37^iv^	65.84 (5)	Al57^i^—Al58—Al46^x^	142.26 (7)
Cr10^iv^—Cr19—Al37^iv^	63.50 (5)	Al54^x^—Al58—Al46^x^	88.31 (6)
Cr14—Cr19—Al37^iv^	63.29 (5)	Cr2^i^—Al58—Al46^x^	148.61 (7)
Cr4—Cr19—Al33	64.97 (4)	Cr2^x^—Al58—Al46^x^	113.11 (6)
Cr10^iv^—Cr19—Al33	115.99 (6)	Al55^x^—Al58—Al46^x^	56.64 (5)
Cr14—Cr19—Al33	62.89 (4)	Al56—Al58—Al46^x^	105.17 (6)
Al37^iv^—Cr19—Al33	64.14 (5)	Al60—Al58—Al46^x^	90.98 (6)
Cr4—Cr19—Al9^iv^	66.86 (5)	Al57^i^—Al58—Al59	59.85 (5)
Cr10^iv^—Cr19—Al9^iv^	62.45 (5)	Al54^x^—Al58—Al59	59.69 (5)
Cr14—Cr19—Al9^iv^	115.41 (6)	Cr2^i^—Al58—Al59	59.13 (4)
Al37^iv^—Cr19—Al9^iv^	63.99 (5)	Cr2^x^—Al58—Al59	59.12 (4)
Al33—Cr19—Al9^iv^	119.43 (7)	Al55^x^—Al58—Al59	108.38 (6)
Cr4—Cr19—Al33^iv^	64.00 (4)	Al56—Al58—Al59	108.53 (6)
Cr10^iv^—Cr19—Al33^iv^	120.54 (6)	Al60—Al58—Al59	107.18 (6)
Cr14—Cr19—Al33^iv^	175.53 (7)	Al46^x^—Al58—Al59	146.27 (7)
Al37^iv^—Cr19—Al33^iv^	120.83 (7)	Al57^i^—Al58—Al47^iv^	88.19 (6)
Al33—Cr19—Al33^iv^	116.60 (6)	Al54^x^—Al58—Al47^iv^	142.46 (7)
Al9^iv^—Cr19—Al33^iv^	68.88 (5)	Cr2^i^—Al58—Al47^iv^	113.02 (6)
Cr4—Cr19—Al64^iv^	121.98 (6)	Cr2^x^—Al58—Al47^iv^	148.48 (7)
Cr10^iv^—Cr19—Al64^iv^	61.59 (4)	Al55^x^—Al58—Al47^iv^	105.25 (6)
Cr14—Cr19—Al64^iv^	111.30 (6)	Al56—Al58—Al47^iv^	56.47 (5)
Al37^iv^—Cr19—Al64^iv^	117.59 (6)	Al60—Al58—Al47^iv^	90.88 (6)
Al33—Cr19—Al64^iv^	173.05 (7)	Al46^x^—Al58—Al47^iv^	57.66 (5)
Al9^iv^—Cr19—Al64^iv^	65.95 (5)	Al59—Al58—Al47^iv^	146.35 (7)
Al33^iv^—Cr19—Al64^iv^	68.86 (5)	Al53—Al59—Al53^xi^	115.19 (6)
Cr4—Cr19—Cr15	114.30 (5)	Al53—Al59—Al54^x^	155.70 (7)
Cr10^iv^—Cr19—Cr15	117.63 (6)	Al53^xi^—Al59—Al54^x^	60.47 (5)
Cr14—Cr19—Cr15	65.93 (4)	Al53—Al59—Cr2^i^	53.96 (4)
Al37^iv^—Cr19—Cr15	118.16 (6)	Al53^xi^—Al59—Cr2^i^	102.45 (6)
Al33—Cr19—Cr15	62.68 (5)	Al54^x^—Al59—Cr2^i^	102.30 (6)
Al9^iv^—Cr19—Cr15	177.77 (7)	Al53—Al59—Cr2^x^	102.45 (6)
Al33^iv^—Cr19—Cr15	109.74 (6)	Al53^xi^—Al59—Cr2^x^	53.93 (4)
Al64^iv^—Cr19—Cr15	112.00 (6)	Al54^x^—Al59—Cr2^x^	54.63 (4)
Al5—Al16—Al23^ix^	102.58 (6)	Cr2^i^—Al59—Cr2^x^	56.88 (4)
Al5—Al16—Al62^i^	103.71 (6)	Al53—Al59—Al57^i^	60.52 (5)
Al23^ix^—Al16—Al62^i^	118.14 (7)	Al53^xi^—Al59—Al57^i^	155.61 (7)
Cr16—Al16—Al28	59.45 (5)	Al54^x^—Al59—Al57^i^	112.45 (6)
Al5—Al16—Al28	59.45 (5)	Cr2^i^—Al59—Al57^i^	54.55 (4)
Al23^ix^—Al16—Al28	159.59 (7)	Cr2^x^—Al59—Al57^i^	102.22 (6)
Al62^i^—Al16—Al28	62.60 (5)	Al53—Al59—Al59^xi^	58.48 (6)
Cr16—Al16—Al18	57.71 (5)	Al53^xi^—Al59—Al59^xi^	58.44 (6)
Al5—Al16—Al18	57.71 (5)	Al54^x^—Al59—Al59^xi^	107.17 (7)
Al23^ix^—Al16—Al18	63.51 (5)	Cr2^i^—Al59—Al59^xi^	59.01 (4)
Al62^i^—Al16—Al18	159.49 (7)	Cr2^x^—Al59—Al59^xi^	58.97 (4)
Al28—Al16—Al18	108.25 (6)	Al57^i^—Al59—Al59^xi^	107.18 (7)
Al5—Al16—Cr6^i^	56.98 (4)	Al53—Al59—Al58	105.75 (6)
Al23^ix^—Al16—Cr6^i^	122.22 (6)	Al53^xi^—Al59—Al58	105.81 (6)
Al62^i^—Al16—Cr6^i^	119.15 (6)	Al54^x^—Al59—Al58	57.47 (5)
Al28—Al16—Cr6^i^	58.42 (4)	Cr2^i^—Al59—Al58	58.68 (4)
Al18—Al16—Cr6^i^	60.43 (4)	Cr2^x^—Al59—Al58	58.74 (4)
Cr16—Al16—Cr9	55.48 (4)	Al57^i^—Al59—Al58	57.29 (5)
Al5—Al16—Cr9	55.48 (4)	Al59^xi^—Al59—Al58	107.94 (4)
Al23^ix^—Al16—Cr9	54.30 (4)	Al53—Al59—Al38	58.87 (5)
Al62^i^—Al16—Cr9	101.79 (6)	Al53^xi^—Al59—Al38	108.95 (6)
Al28—Al16—Cr9	105.30 (6)	Al54^x^—Al59—Al38	145.03 (7)
Al18—Al16—Cr9	61.50 (4)	Cr2^i^—Al59—Al38	112.66 (6)
Cr6^i^—Al16—Cr9	106.43 (5)	Cr2^x^—Al59—Al38	149.29 (6)
Cr16—Al16—Cr13	55.45 (4)	Al57^i^—Al59—Al38	89.56 (6)
Al5—Al16—Cr13	55.45 (4)	Al59^xi^—Al59—Al38	90.53 (5)
Al23^ix^—Al16—Cr13	102.16 (6)	Al58—Al59—Al38	145.25 (7)
Al62^i^—Al16—Cr13	54.77 (4)	Al53—Al59—Al40^xi^	108.99 (6)
Al28—Al16—Cr13	60.54 (4)	Al53^xi^—Al59—Al40^xi^	58.75 (5)
Al18—Al16—Cr13	104.77 (6)	Al54^x^—Al59—Al40^xi^	89.37 (6)
Cr6^i^—Al16—Cr13	104.18 (5)	Cr2^i^—Al59—Al40^xi^	149.26 (6)
Cr9—Al16—Cr13	54.66 (3)	Cr2^x^—Al59—Al40^xi^	112.52 (6)
Al5—Al16—Al30^ix^	99.66 (6)	Al57^i^—Al59—Al40^xi^	145.25 (7)
Al23^ix^—Al16—Al30^ix^	86.03 (6)	Al59^xi^—Al59—Al40^xi^	90.45 (5)
Al62^i^—Al16—Al30^ix^	140.94 (7)	Al58—Al59—Al40^xi^	145.26 (7)
Al28—Al16—Al30^ix^	105.53 (6)	Al38—Al59—Al40^xi^	59.89 (5)
Al18—Al16—Al30^ix^	57.38 (5)	Al53—Al59—Al41^iv^	89.21 (6)
Cr6^i^—Al16—Al30^ix^	52.65 (4)	Al53^xi^—Al59—Al41^iv^	143.89 (7)
Cr9—Al16—Al30^ix^	117.25 (6)	Al54^x^—Al59—Al41^iv^	107.35 (6)
Cr13—Al16—Al30^ix^	154.80 (6)	Cr2^i^—Al59—Al41^iv^	113.57 (6)
Cr16—Al16—Al40	102.00 (6)	Cr2^x^—Al59—Al41^iv^	149.71 (6)
Al5—Al16—Al40	102.00 (6)	Al57^i^—Al59—Al41^iv^	59.32 (5)
Al23^ix^—Al16—Al40	60.40 (5)	Al59^xi^—Al59—Al41^iv^	145.47 (8)
Al62^i^—Al16—Al40	59.85 (5)	Al58—Al59—Al41^iv^	91.30 (6)
Al28—Al16—Al40	111.10 (6)	Al38—Al59—Al41^iv^	59.85 (5)
Al18—Al16—Al40	112.34 (6)	Al40^xi^—Al59—Al41^iv^	89.13 (6)
Cr6^i^—Al16—Al40	158.86 (6)	Al53—Al59—Al25^x^	143.84 (7)
Cr9—Al16—Al40	56.33 (4)	Al53^xi^—Al59—Al25^x^	89.17 (6)
Cr13—Al16—Al40	56.77 (4)	Al54^x^—Al59—Al25^x^	59.32 (5)
Al30^ix^—Al16—Al40	143.17 (7)	Cr2^i^—Al59—Al25^x^	149.80 (6)
Al5—Al16—Al36^i^	99.74 (6)	Cr2^x^—Al59—Al25^x^	113.63 (6)
Al23^ix^—Al16—Al36^i^	141.41 (7)	Al57^i^—Al59—Al25^x^	107.44 (6)
Al62^i^—Al16—Al36^i^	85.98 (6)	Al59^xi^—Al59—Al25^x^	145.37 (8)
Al28—Al16—Al36^i^	57.09 (5)	Al58—Al59—Al25^x^	91.46 (6)
Al18—Al16—Al36^i^	104.68 (6)	Al38—Al59—Al25^x^	88.93 (6)
Cr6^i^—Al16—Al36^i^	50.33 (4)	Al40^xi^—Al59—Al25^x^	59.77 (5)
Cr9—Al16—Al36^i^	155.05 (6)	Al41^iv^—Al59—Al25^x^	58.25 (5)
Cr13—Al16—Al36^i^	116.43 (6)	Cr2^x^—Al60—Cr2^i^	58.35 (5)
Al30^ix^—Al16—Al36^i^	59.31 (5)	Cr2^x^—Al60—Al56	106.71 (7)
Al40—Al16—Al36^i^	142.89 (7)	Cr2^i^—Al60—Al56	57.36 (4)
Al2^i^—Al17—Al27	100.06 (6)	Cr2^x^—Al60—Al56^xi^	57.36 (4)
Al2^i^—Al17—Cr6	56.53 (4)	Cr2^i^—Al60—Al56^xi^	106.70 (7)
Al27—Al17—Cr6	118.65 (7)	Al56—Al60—Al56^xi^	163.41 (10)
Al2^i^—Al17—Al44	150.77 (7)	Cr2^x^—Al60—Al55^x^	57.30 (4)
Al27—Al17—Al44	108.31 (6)	Cr2^i^—Al60—Al55^x^	106.56 (7)
Cr6—Al17—Al44	112.57 (6)	Al56—Al60—Al55^x^	115.83 (4)
Al2^i^—Al17—Al26^vii^	56.79 (5)	Al56^xi^—Al60—Al55^x^	61.45 (4)
Al27—Al17—Al26^vii^	61.57 (5)	Cr2^x^—Al60—Al55^i^	106.56 (7)
Cr6—Al17—Al26^vii^	58.63 (4)	Cr2^i^—Al60—Al55^i^	57.30 (4)
Al44—Al17—Al26^vii^	145.50 (7)	Al56—Al60—Al55^i^	61.45 (4)
Al2^i^—Al17—Al27^vii^	99.46 (6)	Al56^xi^—Al60—Al55^i^	115.83 (4)
Al27—Al17—Al27^vii^	84.00 (6)	Al55^x^—Al60—Al55^i^	163.19 (10)
Cr6—Al17—Al27^vii^	52.76 (4)	Cr2^x^—Al60—Al58^xi^	59.81 (5)
Al44—Al17—Al27^vii^	90.52 (6)	Cr2^i^—Al60—Al58^xi^	59.90 (5)
Al26^vii^—Al17—Al27^vii^	56.83 (5)	Al56—Al60—Al58^xi^	109.59 (6)
Al2^i^—Al17—Cr8^i^	54.14 (4)	Al56^xi^—Al60—Al58^xi^	59.92 (4)
Al27—Al17—Cr8^i^	54.15 (4)	Al55^x^—Al60—Al58^xi^	109.49 (6)
Cr6—Al17—Cr8^i^	104.59 (5)	Al55^i^—Al60—Al58^xi^	59.88 (4)
Al44—Al17—Cr8^i^	142.56 (7)	Cr2^x^—Al60—Al58	59.89 (5)
Al26^vii^—Al17—Cr8^i^	61.37 (4)	Cr2^i^—Al60—Al58	59.81 (5)
Al27^vii^—Al17—Cr8^i^	116.50 (6)	Al56—Al60—Al58	59.92 (4)
Al2^i^—Al17—Al36	99.42 (6)	Al56^xi^—Al60—Al58	109.58 (6)
Al27—Al17—Al36	141.82 (7)	Al55^x^—Al60—Al58	59.88 (4)
Cr6—Al17—Al36	51.09 (4)	Al55^i^—Al60—Al58	109.49 (6)
Al44—Al17—Al36	61.86 (5)	Al58^xi^—Al60—Al58	109.78 (9)
Al26^vii^—Al17—Al36	104.27 (6)	Cr2^x^—Al60—Al43^i^	147.98 (4)
Al27^vii^—Al17—Al36	60.56 (5)	Cr2^i^—Al60—Al43^i^	112.00 (3)
Cr8^i^—Al17—Al36	153.46 (6)	Al56—Al60—Al43^i^	87.50 (5)
Al2^i^—Al17—Al42^i^	100.82 (6)	Al56^xi^—Al60—Al43^i^	104.22 (5)
Al27—Al17—Al42^i^	58.24 (5)	Al55^x^—Al60—Al43^i^	141.38 (8)
Cr6—Al17—Al42^i^	157.20 (6)	Al55^i^—Al60—Al43^i^	54.79 (4)
Al44—Al17—Al42^i^	88.52 (6)	Al58^xi^—Al60—Al43^i^	88.56 (4)
Al26^vii^—Al17—Al42^i^	108.70 (6)	Al58—Al60—Al43^i^	146.15 (4)
Al27^vii^—Al17—Al42^i^	139.58 (7)	Cr2^x^—Al60—Al43^x^	112.00 (3)
Cr8^i^—Al17—Al42^i^	54.05 (4)	Cr2^i^—Al60—Al43^x^	147.98 (4)
Al36—Al17—Al42^i^	146.91 (7)	Al56—Al60—Al43^x^	104.22 (5)
Al2^i^—Al17—Al63^i^	56.55 (5)	Al56^xi^—Al60—Al43^x^	87.50 (5)
Al27—Al17—Al63^i^	101.75 (6)	Al55^x^—Al60—Al43^x^	54.79 (4)
Cr6—Al17—Al63^i^	105.30 (5)	Al55^i^—Al60—Al43^x^	141.38 (8)
Al44—Al17—Al63^i^	109.39 (7)	Al58^xi^—Al60—Al43^x^	146.15 (4)
Al26^vii^—Al17—Al63^i^	105.05 (6)	Al58—Al60—Al43^x^	88.56 (4)
Al27^vii^—Al17—Al63^i^	155.84 (7)	Al43^i^—Al60—Al43^x^	91.13 (8)
Cr8^i^—Al17—Al63^i^	54.10 (4)	Cr2^x^—Al60—Al42^x^	112.04 (3)
Al36—Al17—Al63^i^	116.39 (6)	Cr2^i^—Al60—Al42^x^	148.27 (4)
Al42^i^—Al17—Al63^i^	57.28 (5)	Al56—Al60—Al42^x^	141.21 (8)
Al5—Al18—Al30^ix^	104.05 (6)	Al56^xi^—Al60—Al42^x^	54.77 (4)
Cr16—Al18—Cr5	56.37 (4)	Al55^x^—Al60—Al42^x^	87.47 (5)
Al5—Al18—Cr5	56.37 (4)	Al55^i^—Al60—Al42^x^	104.44 (5)
Al30^ix^—Al18—Cr5	103.23 (6)	Al58^xi^—Al60—Al42^x^	88.76 (4)
Al5—Al18—Al3^vi^	102.12 (6)	Al58—Al60—Al42^x^	146.01 (4)
Al30^ix^—Al18—Al3^vi^	120.22 (7)	Al43^i^—Al60—Al42^x^	58.21 (5)
Cr5—Al18—Al3^vi^	53.46 (4)	Al43^x^—Al60—Al42^x^	62.87 (5)
Cr16—Al18—Al16	58.71 (5)	Cr2^x^—Al60—Al42^i^	148.27 (4)
Al5—Al18—Al16	58.71 (5)	Cr2^i^—Al60—Al42^i^	112.04 (3)
Al30^ix^—Al18—Al16	62.29 (5)	Al56—Al60—Al42^i^	54.77 (4)
Cr5—Al18—Al16	105.49 (6)	Al56^xi^—Al60—Al42^i^	141.21 (8)
Al3^vi^—Al18—Al16	158.84 (7)	Al55^x^—Al60—Al42^i^	104.44 (5)
Cr16—Al18—Al19	58.60 (5)	Al55^i^—Al60—Al42^i^	87.47 (5)
Al5—Al18—Al19	58.60 (5)	Al58^xi^—Al60—Al42^i^	146.01 (4)
Al30^ix^—Al18—Al19	159.67 (7)	Al58—Al60—Al42^i^	88.76 (4)
Cr5—Al18—Al19	59.25 (4)	Al43^i^—Al60—Al42^i^	62.87 (5)
Al3^vi^—Al18—Al19	59.73 (5)	Al43^x^—Al60—Al42^i^	58.21 (5)
Al16—Al18—Al19	109.96 (6)	Al42^x^—Al60—Al42^i^	90.84 (8)
Al5—Al18—Cr6^i^	57.07 (4)	Cr14—Al61—Cr13	164.18 (7)
Al30^ix^—Al18—Cr6^i^	53.88 (4)	Cr14—Al61—Al14	131.43 (6)
Cr5—Al18—Cr6^i^	56.60 (3)	Cr13—Al61—Al14	64.25 (5)
Al3^vi^—Al18—Cr6^i^	103.32 (6)	Cr14—Al61—Al37^iv^	62.98 (5)
Al16—Al18—Cr6^i^	59.85 (4)	Cr13—Al61—Al37^iv^	132.68 (7)
Al19—Al18—Cr6^i^	105.79 (6)	Al14—Al61—Al37^iv^	68.46 (5)
Al5—Al18—Al34^vi^	99.65 (6)	Cr14—Al61—Al48	114.50 (6)
Al30^ix^—Al18—Al34^vi^	138.55 (7)	Cr13—Al61—Al48	54.46 (4)
Cr5—Al18—Al34^vi^	118.21 (6)	Al14—Al61—Al48	106.86 (6)
Al3^vi^—Al18—Al34^vi^	86.55 (6)	Al37^iv^—Al61—Al48	148.42 (7)
Al16—Al18—Al34^vi^	104.52 (6)	Cr14—Al61—Al38	120.71 (6)
Al19—Al18—Al34^vi^	60.07 (5)	Cr13—Al61—Al38	60.93 (4)
Cr6^i^—Al18—Al34^vi^	155.92 (7)	Al14—Al61—Al38	63.37 (5)
Cr16—Al18—Cr9	55.11 (4)	Al37^iv^—Al61—Al38	96.47 (6)
Al5—Al18—Cr9	55.11 (4)	Al48—Al61—Al38	109.40 (6)
Al30^ix^—Al18—Cr9	120.19 (6)	Cr14—Al61—Al49	54.30 (4)
Cr5—Al18—Cr9	104.47 (5)	Cr13—Al61—Al49	114.16 (6)
Al3^vi^—Al18—Cr9	118.97 (6)	Al14—Al61—Al49	149.92 (7)
Al16—Al18—Cr9	59.64 (4)	Al37^iv^—Al61—Al49	107.30 (6)
Al19—Al18—Cr9	60.84 (4)	Al48—Al61—Al49	60.31 (5)
Cr6^i^—Al18—Cr9	104.60 (5)	Al38—Al61—Al49	144.72 (7)
Al34^vi^—Al18—Cr9	52.05 (4)	Cr14—Al61—Al53	111.87 (6)
Al5—Al18—Al39^v^	102.18 (6)	Cr13—Al61—Al53	54.08 (4)
Al30^ix^—Al18—Al39^v^	60.83 (5)	Al14—Al61—Al53	109.23 (6)
Cr5—Al18—Al39^v^	56.14 (4)	Al37^iv^—Al61—Al53	149.39 (7)
Al3^vi^—Al18—Al39^v^	61.58 (5)	Al48—Al61—Al53	62.19 (5)
Al16—Al18—Al39^v^	110.83 (6)	Al38—Al61—Al53	58.78 (5)
Al19—Al18—Al39^v^	109.73 (6)	Al49—Al61—Al53	89.26 (6)
Cr6^i^—Al18—Al39^v^	56.11 (4)	Cr14—Al61—Al29	124.25 (6)
Al34^vi^—Al18—Al39^v^	144.38 (7)	Cr13—Al61—Al29	62.52 (4)
Cr9—Al18—Al39^v^	157.29 (6)	Al14—Al61—Al29	59.70 (5)
Al5—Al18—Al23^ix^	98.51 (6)	Al37^iv^—Al61—Al29	95.04 (6)
Al30^ix^—Al18—Al23^ix^	85.10 (6)	Al48—Al61—Al29	59.09 (5)
Cr5—Al18—Al23^ix^	154.65 (6)	Al38—Al61—Al29	111.77 (6)
Al3^vi^—Al18—Al23^ix^	141.41 (7)	Al49—Al61—Al29	92.09 (6)
Al16—Al18—Al23^ix^	56.91 (5)	Al53—Al61—Al29	110.25 (6)
Al19—Al18—Al23^ix^	106.77 (6)	Cr14—Al61—Al33	61.02 (4)
Cr6^i^—Al18—Al23^ix^	115.27 (6)	Cr13—Al61—Al33	125.44 (6)
Al34^vi^—Al18—Al23^ix^	57.88 (5)	Al14—Al61—Al33	95.57 (6)
Cr9—Al18—Al23^ix^	51.93 (4)	Al37^iv^—Al61—Al33	60.19 (5)
Al39^v^—Al18—Al23^ix^	143.42 (6)	Al48—Al61—Al33	90.29 (6)
Al5—Al19—Al20^vi^	102.42 (6)	Al38—Al61—Al33	154.18 (7)
Cr16—Al19—Cr5	56.04 (4)	Al49—Al61—Al33	59.74 (5)
Al5—Al19—Cr5	56.04 (4)	Al53—Al61—Al33	146.91 (7)
Al20^vi^—Al19—Cr5	102.03 (6)	Al29—Al61—Al33	63.65 (5)
Al5—Al19—Al3^vi^	100.99 (6)	Cr14—Al61—Al41^iv^	59.75 (4)
Al20^vi^—Al19—Al3^vi^	121.93 (7)	Cr13—Al61—Al41^iv^	121.85 (6)
Cr5—Al19—Al3^vi^	53.34 (4)	Al14—Al61—Al41^iv^	97.03 (6)
Cr16—Al19—Al18	57.49 (5)	Al37^iv^—Al61—Al41^iv^	62.06 (5)
Al5—Al19—Al18	57.49 (5)	Al48—Al61—Al41^iv^	146.97 (7)
Al20^vi^—Al19—Al18	157.41 (7)	Al38—Al61—Al41^iv^	61.58 (5)
Cr5—Al19—Al18	59.28 (4)	Al49—Al61—Al41^iv^	107.16 (6)
Al3^vi^—Al19—Al18	59.32 (5)	Al53—Al61—Al41^iv^	88.78 (6)
Al5—Al19—Al34^vi^	98.91 (6)	Al29—Al61—Al41^iv^	153.32 (7)
Al20^vi^—Al19—Al34^vi^	139.66 (7)	Al33—Al61—Al41^iv^	110.09 (6)
Cr5—Al19—Al34^vi^	118.28 (6)	Cr14—Al61—Al57^i^	53.80 (4)
Al3^vi^—Al19—Al34^vi^	86.35 (6)	Cr13—Al61—Al57^i^	112.05 (6)
Al18—Al19—Al34^vi^	60.11 (5)	Al14—Al61—Al57^i^	150.69 (7)
Cr16—Al19—Al14	58.04 (5)	Al37^iv^—Al61—Al57^i^	108.06 (6)
Al5—Al19—Al14	58.04 (5)	Al48—Al61—Al57^i^	90.68 (6)
Al20^vi^—Al19—Al14	61.59 (5)	Al38—Al61—Al57^i^	88.94 (6)
Cr5—Al19—Al14	104.51 (6)	Al49—Al61—Al57^i^	59.35 (5)
Al3^vi^—Al19—Al14	157.49 (7)	Al53—Al61—Al57^i^	58.15 (5)
Al18—Al19—Al14	108.25 (6)	Al29—Al61—Al57^i^	147.19 (7)
Al34^vi^—Al19—Al14	103.91 (6)	Al33—Al61—Al57^i^	107.91 (6)
Al5—Al19—Cr7^vi^	56.56 (4)	Al41^iv^—Al61—Al57^i^	58.91 (5)
Al20^vi^—Al19—Cr7^vi^	53.03 (4)	Cr12—Al62—Cr13^i^	164.00 (7)
Cr5—Al19—Cr7^vi^	55.69 (3)	Cr12—Al62—Al16^i^	131.20 (6)
Al3^vi^—Al19—Cr7^vi^	103.09 (6)	Cr13^i^—Al62—Al16^i^	64.69 (5)
Al18—Al19—Cr7^vi^	104.42 (6)	Cr12—Al62—Al45	63.35 (5)
Al34^vi^—Al19—Cr7^vi^	154.74 (7)	Cr13^i^—Al62—Al45	132.52 (7)
Al14—Al19—Cr7^vi^	59.77 (4)	Al16^i^—Al62—Al45	67.85 (5)
Cr16—Al19—Cr9	54.74 (4)	Cr12—Al62—Al40^i^	121.05 (6)
Al5—Al19—Cr9	54.74 (4)	Cr13^i^—Al62—Al40^i^	60.86 (5)
Al20^vi^—Al19—Cr9	118.65 (6)	Al16^i^—Al62—Al40^i^	63.40 (5)
Cr5—Al19—Cr9	104.32 (5)	Al45—Al62—Al40^i^	96.43 (6)
Al3^vi^—Al19—Cr9	118.34 (6)	Cr12—Al62—Al48^i^	114.40 (6)
Al18—Al19—Cr9	60.62 (4)	Cr13^i^—Al62—Al48^i^	54.28 (4)
Al34^vi^—Al19—Cr9	52.00 (4)	Al16^i^—Al62—Al48^i^	107.03 (6)
Al14—Al19—Cr9	58.56 (4)	Al45—Al62—Al48^i^	148.32 (7)
Cr7^vi^—Al19—Cr9	103.65 (5)	Al40^i^—Al62—Al48^i^	109.26 (6)
Al5—Al19—Al32^vi^	102.41 (6)	Cr12—Al62—Al52	54.40 (4)
Al20^vi^—Al19—Al32^vi^	61.49 (5)	Cr13^i^—Al62—Al52	113.76 (6)
Cr5—Al19—Al32^vi^	56.06 (4)	Al16^i^—Al62—Al52	149.88 (7)
Al3^vi^—Al19—Al32^vi^	61.90 (5)	Al45—Al62—Al52	107.78 (6)
Al18—Al19—Al32^vi^	109.69 (6)	Al40^i^—Al62—Al52	144.66 (7)
Al34^vi^—Al19—Al32^vi^	144.42 (7)	Al48^i^—Al62—Al52	60.10 (5)
Al14—Al19—Al32^vi^	111.51 (6)	Cr12—Al62—Al53^i^	111.95 (6)
Cr7^vi^—Al19—Al32^vi^	56.79 (4)	Cr13^i^—Al62—Al53^i^	53.93 (4)
Cr9—Al19—Al32^vi^	157.15 (6)	Al16^i^—Al62—Al53^i^	109.52 (6)
Cr16—Al19—Al20	97.91 (6)	Al45—Al62—Al53^i^	149.60 (7)
Al5—Al19—Al20	97.91 (6)	Al40^i^—Al62—Al53^i^	58.86 (5)
Al20^vi^—Al19—Al20	85.22 (6)	Al48^i^—Al62—Al53^i^	62.07 (5)
Cr5—Al19—Al20	153.80 (7)	Al52—Al62—Al53^i^	88.99 (6)
Al3^vi^—Al19—Al20	141.85 (7)	Cr12—Al62—Al28^i^	124.07 (6)
Al18—Al19—Al20	106.41 (6)	Cr13^i^—Al62—Al28^i^	62.64 (4)
Al34^vi^—Al19—Al20	58.04 (5)	Al16^i^—Al62—Al28^i^	59.63 (5)
Al14—Al19—Al20	56.64 (5)	Al45—Al62—Al28^i^	94.61 (6)
Cr7^vi^—Al19—Al20	114.97 (6)	Al40^i^—Al62—Al28^i^	111.58 (6)
Cr9—Al19—Al20	51.43 (4)	Al48^i^—Al62—Al28^i^	59.24 (5)
Al32^vi^—Al19—Al20	143.88 (6)	Al52—Al62—Al28^i^	92.11 (6)
Cr7—Al20—Cr9	164.04 (7)	Al53^i^—Al62—Al28^i^	110.30 (6)
Cr7—Al20—Al14	131.47 (6)	Cr12—Al62—Al25	59.75 (4)
Cr9—Al20—Al14	64.06 (5)	Cr13^i^—Al62—Al25	122.05 (6)
Cr7—Al20—Al19^vi^	65.23 (5)	Al16^i^—Al62—Al25	97.03 (6)
Cr9—Al20—Al19^vi^	130.14 (7)	Al45—Al62—Al25	62.23 (5)
Al14—Al20—Al19^vi^	66.25 (5)	Al40^i^—Al62—Al25	61.90 (5)
Cr7—Al20—Al34^vi^	114.21 (6)	Al48^i^—Al62—Al25	146.96 (7)
Cr9—Al20—Al34^vi^	55.89 (4)	Al52—Al62—Al25	107.16 (6)
Al14—Al20—Al34^vi^	107.28 (6)	Al53^i^—Al62—Al25	88.95 (6)
Al19^vi^—Al20—Al34^vi^	149.10 (7)	Al28^i^—Al62—Al25	153.21 (7)
Cr7—Al20—Al12	111.18 (5)	Cr12—Al62—Al31	61.18 (4)
Cr9—Al20—Al12	54.01 (3)	Cr13^i^—Al62—Al31	125.22 (6)
Al14—Al20—Al12	109.37 (6)	Al16^i^—Al62—Al31	95.02 (6)
Al19^vi^—Al20—Al12	147.82 (7)	Al45—Al62—Al31	60.11 (5)
Al34^vi^—Al20—Al12	62.95 (5)	Al40^i^—Al62—Al31	153.92 (7)
Cr7—Al20—Al38	120.08 (6)	Al48^i^—Al62—Al31	90.34 (6)
Cr9—Al20—Al38	60.12 (4)	Al52—Al62—Al31	60.22 (5)
Al14—Al20—Al38	63.04 (5)	Al53^i^—Al62—Al31	147.03 (7)
Al19^vi^—Al20—Al38	93.88 (6)	Al28^i^—Al62—Al31	63.32 (5)
Al34^vi^—Al20—Al38	110.33 (6)	Al25—Al62—Al31	110.09 (6)
Al12—Al20—Al38	59.06 (6)	Cr12—Al62—Al54	54.01 (4)
Cr7—Al20—Al14^vi^	63.24 (4)	Cr13^i^—Al62—Al54	111.76 (6)
Cr9—Al20—Al14^vi^	125.32 (6)	Al16^i^—Al62—Al54	150.84 (7)
Al14—Al20—Al14^vi^	93.04 (6)	Al45—Al62—Al54	108.61 (6)
Al19^vi^—Al20—Al14^vi^	60.63 (5)	Al40^i^—Al62—Al54	89.12 (6)
Al34^vi^—Al20—Al14^vi^	90.74 (6)	Al48^i^—Al62—Al54	90.48 (6)
Al12—Al20—Al14^vi^	149.14 (7)	Al52—Al62—Al54	59.24 (5)
Al38—Al20—Al14^vi^	151.50 (7)	Al53^i^—Al62—Al54	58.03 (5)
Cr7—Al20—Al7^vi^	54.78 (4)	Al28^i^—Al62—Al54	147.15 (7)
Cr9—Al20—Al7^vi^	114.72 (6)	Al25—Al62—Al54	59.04 (5)
Al14—Al20—Al7^vi^	149.88 (7)	Al31—Al62—Al54	108.30 (6)
Al19^vi^—Al20—Al7^vi^	109.93 (6)	Cr8—Al63—Al2	56.81 (4)
Al34^vi^—Al20—Al7^vi^	59.57 (5)	Cr8—Al63—Al51	143.97 (7)
Al12—Al20—Al7^vi^	89.24 (6)	Al2—Al63—Al51	111.79 (7)
Al38—Al20—Al7^vi^	145.09 (7)	Cr8—Al63—Al50^i^	144.01 (7)
Al14^vi^—Al20—Al7^vi^	62.25 (5)	Al2—Al63—Al50^i^	111.37 (7)
Cr7—Al20—Al32	60.37 (4)	Al51—Al63—Al50^i^	71.05 (6)
Cr9—Al20—Al32	119.75 (6)	Cr8—Al63—Al43	57.27 (4)
Al14—Al20—Al32	96.15 (6)	Al2—Al63—Al43	104.42 (6)
Al19^vi^—Al20—Al32	62.00 (5)	Al51—Al63—Al43	100.54 (6)
Al34^vi^—Al20—Al32	147.10 (7)	Al50^i^—Al63—Al43	143.85 (7)
Al12—Al20—Al32	87.89 (6)	Cr8—Al63—Al42	57.41 (4)
Al38—Al20—Al32	60.31 (5)	Al2—Al63—Al42	104.01 (6)
Al14^vi^—Al20—Al32	111.15 (6)	Al51—Al63—Al42	143.92 (7)
Al7^vi^—Al20—Al32	108.36 (6)	Al50^i^—Al63—Al42	100.31 (6)
Cr7—Al20—Al19	124.86 (6)	Al43—Al63—Al42	65.29 (5)
Cr9—Al20—Al19	63.33 (5)	Cr8—Al63—Al55	112.50 (6)
Al14—Al20—Al19	59.76 (5)	Al2—Al63—Al55	149.29 (7)
Al19^vi^—Al20—Al19	94.78 (6)	Al51—Al63—Al55	57.72 (5)
Al34^vi^—Al20—Al19	59.07 (5)	Al50^i^—Al63—Al55	93.05 (6)
Al12—Al20—Al19	110.64 (5)	Al43—Al63—Al55	55.58 (5)
Al38—Al20—Al19	111.68 (6)	Al42—Al63—Al55	88.89 (6)
Al14^vi^—Al20—Al19	62.31 (5)	Cr8—Al63—Al56^i^	112.55 (6)
Al7^vi^—Al20—Al19	91.96 (6)	Al2—Al63—Al56^i^	148.58 (7)
Al32—Al20—Al19	152.95 (7)	Al51—Al63—Al56^i^	93.06 (6)
Cr7—Al20—Al23^vi^	53.11 (4)	Al50^i^—Al63—Al56^i^	57.66 (5)
Cr9—Al20—Al23^vi^	112.01 (6)	Al43—Al63—Al56^i^	88.81 (6)
Al14—Al20—Al23^vi^	150.56 (7)	Al42—Al63—Al56^i^	55.51 (5)
Al19^vi^—Al20—Al23^vi^	108.96 (6)	Al55—Al63—Al56^i^	60.76 (5)
Al34^vi^—Al20—Al23^vi^	90.85 (6)	Cr8—Al63—Al8	60.60 (4)
Al12—Al20—Al23^vi^	58.12 (4)	Al2—Al63—Al8	57.55 (5)
Al38—Al20—Al23^vi^	89.22 (5)	Al51—Al63—Al8	84.15 (6)
Al14^vi^—Al20—Al23^vi^	110.11 (6)	Al50^i^—Al63—Al8	146.85 (7)
Al7^vi^—Al20—Al23^vi^	59.51 (5)	Al43—Al63—Al8	60.76 (5)
Al32—Al20—Al23^vi^	59.19 (5)	Al42—Al63—Al8	112.56 (6)
Al19—Al20—Al23^vi^	147.42 (6)	Al55—Al63—Al8	91.81 (6)
Al11—Al21—Al57^xvi^	102.55 (6)	Al56^i^—Al63—Al8	148.10 (7)
Al11—Al21—Cr14^iv^	55.60 (4)	Cr8—Al63—Al17^i^	61.19 (4)
Al57^xvi^—Al21—Cr14^iv^	53.27 (4)	Al2—Al63—Al17^i^	57.16 (5)
Cr17—Al21—Al22	58.49 (5)	Al51—Al63—Al17^i^	146.41 (7)
Al11—Al21—Al22	58.49 (5)	Al50^i^—Al63—Al17^i^	83.60 (6)
Al57^xvi^—Al21—Al22	158.51 (7)	Al43—Al63—Al17^i^	112.82 (6)
Cr14^iv^—Al21—Al22	105.24 (6)	Al42—Al63—Al17^i^	60.73 (5)
Cr17—Al21—Cr11	56.03 (4)	Al55—Al63—Al17^i^	148.00 (7)
Al11—Al21—Cr11	56.03 (4)	Al56^i^—Al63—Al17^i^	91.48 (6)
Al57^xvi^—Al21—Cr11	118.56 (6)	Al8—Al63—Al17^i^	108.29 (6)
Cr14^iv^—Al21—Cr11	103.18 (5)	Cr17—Al64—Cr10	56.88 (4)
Al22—Al21—Cr11	61.53 (4)	Al11—Al64—Cr10	56.88 (4)
Al11—Al21—Al10^iv^	101.17 (6)	Al11—Al64—Al15^iv^	55.96 (5)
Al57^xvi^—Al21—Al10^iv^	118.79 (7)	Cr10—Al64—Al15^iv^	55.86 (4)
Cr14^iv^—Al21—Al10^iv^	101.74 (6)	Cr17—Al64—Cr12	55.51 (4)
Al22—Al21—Al10^iv^	61.22 (5)	Al11—Al64—Cr12	55.51 (4)
Cr11—Al21—Al10^iv^	121.58 (6)	Cr10—Al64—Cr12	105.23 (5)
Al11—Al21—Al65^iv^	58.05 (5)	Al15^iv^—Al64—Cr12	104.28 (6)
Al57^xvi^—Al21—Al65^iv^	62.39 (5)	Cr17—Al64—Al51	151.50 (7)
Cr14^iv^—Al21—Al65^iv^	59.12 (4)	Al11—Al64—Al51	151.50 (7)
Al22—Al21—Al65^iv^	108.55 (6)	Cr10—Al64—Al51	140.64 (7)
Cr11—Al21—Al65^iv^	57.91 (4)	Al15^iv^—Al64—Al51	110.39 (6)
Al10^iv^—Al21—Al65^iv^	156.82 (7)	Cr12—Al64—Al51	114.10 (6)
Al11—Al21—Al10^iii^	99.83 (6)	Cr17—Al64—Al22	57.91 (5)
Al57^xvi^—Al21—Al10^iii^	139.35 (7)	Al11—Al64—Al22	57.91 (5)
Cr14^iv^—Al21—Al10^iii^	154.45 (7)	Cr10—Al64—Al22	60.11 (4)
Al22—Al21—Al10^iii^	59.79 (5)	Al15^iv^—Al64—Al22	104.54 (6)
Cr11—Al21—Al10^iii^	52.10 (4)	Cr12—Al64—Al22	59.71 (4)
Al10^iv^—Al21—Al10^iii^	89.07 (6)	Al51—Al64—Al22	144.76 (7)
Al65^iv^—Al21—Al10^iii^	103.75 (6)	Cr17—Al64—Al46	105.54 (6)
Cr17—Al21—Cr10	55.58 (4)	Al11—Al64—Al46	105.54 (6)
Al11—Al21—Cr10	55.58 (4)	Cr10—Al64—Al46	54.99 (4)
Al57^xvi^—Al21—Cr10	102.62 (6)	Al15^iv^—Al64—Al46	102.85 (6)
Cr14^iv^—Al21—Cr10	56.74 (3)	Cr12—Al64—Al46	122.62 (6)
Al22—Al21—Cr10	59.29 (4)	Al51—Al64—Al46	101.91 (6)
Cr11—Al21—Cr10	104.95 (5)	Al22—Al64—Al46	64.96 (5)
Al10^iv^—Al21—Cr10	52.00 (4)	Cr17—Al64—Al52	99.08 (6)
Al65^iv^—Al21—Cr10	104.85 (6)	Al11—Al64—Al52	99.08 (6)
Al10^iii^—Al21—Cr10	118.02 (6)	Cr10—Al64—Al52	155.59 (6)
Al11—Al21—Al22^xvii^	148.53 (7)	Al15^iv^—Al64—Al52	116.69 (6)
Al57^xvi^—Al21—Al22^xvii^	108.37 (6)	Cr12—Al64—Al52	51.73 (4)
Cr14^iv^—Al21—Al22^xvii^	145.56 (6)	Al51—Al64—Al52	62.78 (5)
Al22—Al21—Al22^xvii^	90.04 (6)	Al22—Al64—Al52	105.35 (6)
Cr11—Al21—Al22^xvii^	111.22 (6)	Al46—Al64—Al52	140.35 (7)
Al10^iv^—Al21—Al22^xvii^	58.63 (5)	Cr17—Al64—Al54	98.84 (6)
Al65^iv^—Al21—Al22^xvii^	144.54 (7)	Al11—Al64—Al54	98.84 (6)
Al10^iii^—Al21—Al22^xvii^	59.35 (5)	Cr10—Al64—Al54	116.02 (6)
Cr10—Al21—Al22^xvii^	110.61 (6)	Al15^iv^—Al64—Al54	154.56 (7)
Cr17—Al21—Al41	101.16 (6)	Cr12—Al64—Al54	52.12 (4)
Al11—Al21—Al41	101.16 (6)	Al51—Al64—Al54	90.38 (6)
Al57^xvi^—Al21—Al41	60.21 (5)	Al22—Al64—Al54	57.57 (5)
Cr14^iv^—Al21—Al41	56.47 (4)	Al46—Al64—Al54	86.29 (6)
Al22—Al21—Al41	110.53 (6)	Al52—Al64—Al54	59.23 (5)
Cr11—Al21—Al41	157.10 (6)	Cr17—Al64—Al55	148.44 (7)
Al10^iv^—Al21—Al41	60.22 (5)	Al11—Al64—Al55	148.44 (7)
Al65^iv^—Al21—Al41	110.53 (6)	Cr10—Al64—Al55	111.47 (6)
Al10^iii^—Al21—Al41	145.53 (7)	Al15^iv^—Al64—Al55	146.45 (7)
Cr10—Al21—Al41	56.49 (4)	Cr12—Al64—Al55	109.20 (6)
Al22^xvii^—Al21—Al41	89.40 (5)	Al51—Al64—Al55	57.20 (5)
Cr17—Al22—Al54	102.43 (6)	Al22—Al64—Al55	90.58 (6)
Al11—Al22—Al54	102.43 (6)	Al46—Al64—Al55	56.49 (5)
Cr17—Al22—Cr12	55.34 (4)	Al52—Al64—Al55	86.70 (6)
Al11—Al22—Cr12	55.34 (4)	Al54—Al64—Al55	57.62 (5)
Al54—Al22—Cr12	53.32 (4)	Cr17—Al64—Al9	102.91 (6)
Cr17—Al22—Cr10	56.30 (4)	Al11—Al64—Al9	102.91 (6)
Al11—Al22—Cr10	56.30 (4)	Cr10—Al64—Al9	57.22 (4)
Al54—Al22—Cr10	118.72 (6)	Al15^iv^—Al64—Al9	56.35 (5)
Cr12—Al22—Cr10	103.25 (5)	Cr12—Al64—Al9	158.36 (6)
Cr17—Al22—Al21	58.54 (5)	Al51—Al64—Al9	84.06 (6)
Al11—Al22—Al21	58.54 (5)	Al22—Al64—Al9	112.44 (6)
Al54—Al22—Al21	158.48 (7)	Al46—Al64—Al9	60.36 (5)
Cr12—Al22—Al21	105.17 (6)	Al52—Al64—Al9	142.12 (6)
Cr10—Al22—Al21	61.38 (4)	Al54—Al64—Al9	143.92 (7)
Al11—Al22—Al10^iii^	100.80 (6)	Al55—Al64—Al9	90.28 (6)
Al54—Al22—Al10^iii^	118.83 (7)	Al11^iv^—Al65—Cr11^iv^	56.83 (4)
Cr12—Al22—Al10^iii^	101.54 (6)	Al11^iv^—Al65—Al13	56.12 (5)
Cr10—Al22—Al10^iii^	121.33 (6)	Cr11^iv^—Al65—Al13	55.70 (4)
Al21—Al22—Al10^iii^	61.10 (5)	Cr11^iv^—Al65—Cr18	55.70 (4)
Cr17—Al22—Al64	58.15 (5)	Al11^iv^—Al65—Al50	151.51 (7)
Al11—Al22—Al64	58.15 (5)	Cr11^iv^—Al65—Al50	140.80 (7)
Al54—Al22—Al64	62.24 (5)	Al13—Al65—Al50	110.48 (6)
Cr12—Al22—Al64	59.00 (4)	Cr18—Al65—Al50	110.48 (6)
Cr10—Al22—Al64	58.22 (4)	Al11^iv^—Al65—Cr14	55.63 (4)
Al21—Al22—Al64	108.64 (6)	Cr11^iv^—Al65—Cr14	105.32 (5)
Al10^iii^—Al22—Al64	156.52 (7)	Al13—Al65—Cr14	104.60 (6)
Al11—Al22—Al10^iv^	100.22 (6)	Cr18—Al65—Cr14	104.60 (6)
Al54—Al22—Al10^iv^	139.36 (7)	Al50—Al65—Cr14	113.86 (6)
Cr12—Al22—Al10^iv^	154.60 (6)	Al11^iv^—Al65—Al47^iv^	105.52 (6)
Cr10—Al22—Al10^iv^	52.18 (4)	Cr11^iv^—Al65—Al47^iv^	55.11 (4)
Al21—Al22—Al10^iv^	59.85 (5)	Al13—Al65—Al47^iv^	102.81 (6)
Al10^iii^—Al22—Al10^iv^	89.03 (6)	Al50—Al65—Al47^iv^	101.99 (6)
Al64—Al22—Al10^iv^	104.08 (6)	Cr14—Al65—Al47^iv^	122.43 (6)
Cr17—Al22—Cr11	55.35 (4)	Al11^iv^—Al65—Al21^iv^	57.85 (5)
Al11—Al22—Cr11	55.35 (4)	Cr11^iv^—Al65—Al21^iv^	60.43 (4)
Al54—Al22—Cr11	102.38 (6)	Al13—Al65—Al21^iv^	104.71 (6)
Cr12—Al22—Cr11	56.47 (3)	Al50—Al65—Al21^iv^	144.53 (7)
Cr10—Al22—Cr11	104.99 (5)	Cr14—Al65—Al21^iv^	59.39 (4)
Al21—Al22—Cr11	59.50 (4)	Al47^iv^—Al65—Al21^iv^	65.12 (5)
Al10^iii^—Al22—Cr11	51.90 (4)	Al11^iv^—Al65—Al49	98.92 (6)
Al64—Al22—Cr11	104.65 (6)	Cr11^iv^—Al65—Al49	155.41 (7)
Al10^iv^—Al22—Cr11	118.25 (6)	Al13—Al65—Al49	116.74 (6)
Al11—Al22—Al21^xvii^	148.51 (7)	Cr18—Al65—Al49	116.74 (6)
Al54—Al22—Al21^xvii^	108.49 (6)	Al50—Al65—Al49	62.77 (5)
Cr12—Al22—Al21^xvii^	145.67 (6)	Cr14—Al65—Al49	51.53 (4)
Cr10—Al22—Al21^xvii^	111.02 (6)	Al47^iv^—Al65—Al49	140.35 (7)
Al21—Al22—Al21^xvii^	89.96 (6)	Al21^iv^—Al65—Al49	104.96 (6)
Al10^iii^—Al22—Al21^xvii^	58.86 (5)	Al11^iv^—Al65—Al56	148.30 (7)
Al64—Al22—Al21^xvii^	144.62 (7)	Cr11^iv^—Al65—Al56	111.65 (6)
Al10^iv^—Al22—Al21^xvii^	59.05 (5)	Al13—Al65—Al56	146.50 (7)
Cr11—Al22—Al21^xvii^	110.74 (6)	Cr18—Al65—Al56	146.50 (7)
Cr17—Al22—Al25	100.66 (6)	Al50—Al65—Al56	57.19 (5)
Al11—Al22—Al25	100.66 (6)	Cr14—Al65—Al56	108.82 (6)
Al54—Al22—Al25	60.17 (5)	Al47^iv^—Al65—Al56	56.56 (5)
Cr12—Al22—Al25	56.24 (4)	Al21^iv^—Al65—Al56	90.50 (6)
Cr10—Al22—Al25	156.88 (6)	Al49—Al65—Al56	86.68 (5)
Al21—Al22—Al25	110.47 (6)	Al11^iv^—Al65—Al57^i^	98.70 (6)
Al10^iii^—Al22—Al25	60.29 (5)	Cr11^iv^—Al65—Al57^i^	116.10 (6)
Al64—Al22—Al25	110.18 (6)	Al13—Al65—Al57^i^	154.58 (7)
Al10^iv^—Al22—Al25	145.55 (7)	Al50—Al65—Al57^i^	90.28 (6)
Cr11—Al22—Al25	56.18 (4)	Cr14—Al65—Al57^i^	51.83 (4)
Al21^xvii^—Al22—Al25	89.73 (5)	Al47^iv^—Al65—Al57^i^	86.27 (6)
Cr7^vi^—Al23—Cr9^xv^	164.18 (7)	Al21^iv^—Al65—Al57^i^	57.33 (5)
Cr7^vi^—Al23—Al16^xv^	130.51 (6)	Al49—Al65—Al57^i^	59.18 (5)
Cr9^xv^—Al23—Al16^xv^	64.92 (5)	Al56—Al65—Al57^i^	57.54 (5)
Cr7^vi^—Al23—Al24	63.39 (5)	Cr2^i^—Al66—Cr1	171.58 (7)
Cr9^xv^—Al23—Al24	131.93 (7)	Cr2^i^—Al66—Al55^i^	61.35 (5)
Al16^xv^—Al23—Al24	67.12 (5)	Cr1—Al66—Al55^i^	125.23 (6)
Cr7^vi^—Al23—Al34^iv^	114.09 (6)	Cr2^i^—Al66—Al56	61.38 (5)
Cr9^xv^—Al23—Al34^iv^	56.03 (4)	Cr1—Al66—Al56	125.21 (6)
Al16^xv^—Al23—Al34^iv^	108.08 (6)	Al55^i^—Al66—Al56	63.82 (5)
Al24—Al23—Al34^iv^	148.40 (7)	Cr2^i^—Al66—Al52^i^	117.88 (6)
Cr7^vi^—Al23—Al12^vi^	111.44 (5)	Cr1—Al66—Al52^i^	59.21 (4)
Cr9^xv^—Al23—Al12^vi^	53.95 (3)	Al55^i^—Al66—Al52^i^	91.61 (6)
Al16^xv^—Al23—Al12^vi^	109.93 (6)	Al56—Al66—Al52^i^	153.13 (7)
Al24—Al23—Al12^vi^	148.40 (7)	Cr2^i^—Al66—Al49	117.90 (6)
Al34^iv^—Al23—Al12^vi^	63.19 (5)	Cr1—Al66—Al49	59.20 (4)
Cr7^vi^—Al23—Al40^xv^	120.17 (6)	Al55^i^—Al66—Al49	153.09 (7)
Cr9^xv^—Al23—Al40^xv^	60.25 (4)	Al56—Al66—Al49	91.57 (6)
Al16^xv^—Al23—Al40^xv^	63.10 (5)	Al52^i^—Al66—Al49	109.64 (6)
Al24—Al23—Al40^xv^	95.14 (6)	Cr2^i^—Al66—Al54^i^	57.31 (4)
Al34^iv^—Al23—Al40^xv^	110.70 (6)	Cr1—Al66—Al54^i^	119.74 (6)
Al12^vi^—Al23—Al40^xv^	59.15 (6)	Al55^i^—Al66—Al54^i^	60.05 (5)
Cr7^vi^—Al23—Al8	62.60 (5)	Al56—Al66—Al54^i^	110.30 (6)
Cr9^xv^—Al23—Al8	125.00 (6)	Al52^i^—Al66—Al54^i^	60.72 (5)
Al16^xv^—Al23—Al8	94.82 (6)	Al49—Al66—Al54^i^	145.01 (7)
Al24—Al23—Al8	61.88 (5)	Cr2^i^—Al66—Al57^i^	57.25 (4)
Al34^iv^—Al23—Al8	88.40 (6)	Cr1—Al66—Al57^i^	119.81 (6)
Al12^vi^—Al23—Al8	146.58 (7)	Al55^i^—Al66—Al57^i^	110.22 (6)
Al40^xv^—Al23—Al8	153.97 (7)	Al56—Al66—Al57^i^	60.02 (5)
Cr7^vi^—Al23—Al32^vi^	60.30 (4)	Al52^i^—Al66—Al57^i^	144.99 (7)
Cr9^xv^—Al23—Al32^vi^	119.89 (6)	Al49—Al66—Al57^i^	60.79 (5)
Al16^xv^—Al23—Al32^vi^	95.88 (6)	Al54^i^—Al66—Al57^i^	106.26 (6)
Al24—Al23—Al32^vi^	62.12 (5)	Cr2^i^—Al66—Al51^i^	119.19 (6)
Al34^iv^—Al23—Al32^vi^	147.02 (7)	Cr1—Al66—Al51^i^	67.38 (5)
Al12^vi^—Al23—Al32^vi^	87.76 (6)	Al55^i^—Al66—Al51^i^	57.91 (5)
Al40^xv^—Al23—Al32^vi^	60.39 (5)	Al56—Al66—Al51^i^	93.55 (6)
Al8—Al23—Al32^vi^	112.34 (6)	Al52^i^—Al66—Al51^i^	62.51 (5)
Cr7^vi^—Al23—Al7	54.32 (4)	Al49—Al66—Al51^i^	117.08 (6)
Cr9^xv^—Al23—Al7	114.87 (6)	Al54^i^—Al66—Al51^i^	89.35 (6)
Al16^xv^—Al23—Al7	151.13 (7)	Al57^i^—Al66—Al51^i^	152.46 (7)
Al24—Al23—Al7	108.41 (6)	Cr2^i^—Al66—Al50	119.18 (6)
Al34^iv^—Al23—Al7	59.81 (5)	Cr1—Al66—Al50	67.41 (5)
Al12^vi^—Al23—Al7	88.56 (6)	Al55^i^—Al66—Al50	93.46 (6)
Al40^xv^—Al23—Al7	144.18 (7)	Al56—Al66—Al50	57.87 (5)
Al8—Al23—Al7	60.67 (5)	Al52^i^—Al66—Al50	117.08 (6)
Al32^vi^—Al23—Al7	107.15 (6)	Al49—Al66—Al50	62.56 (5)
Cr7^vi^—Al23—Al18^xv^	124.75 (6)	Al54^i^—Al66—Al50	152.38 (7)
Cr9^xv^—Al23—Al18^xv^	63.41 (4)	Al57^i^—Al66—Al50	89.43 (6)
Al16^xv^—Al23—Al18^xv^	59.59 (5)	Al51^i^—Al66—Al50	68.20 (5)
Al24—Al23—Al18^xv^	94.78 (6)	Cr2^i^—Al66—Al53	55.37 (4)
Al34^iv^—Al23—Al18^xv^	59.56 (5)	Cr1—Al66—Al53	116.21 (6)
Al12^vi^—Al23—Al18^xv^	110.98 (6)	Al55^i^—Al66—Al53	107.68 (6)
Al40^xv^—Al23—Al18^xv^	111.37 (6)	Al56—Al66—Al53	107.74 (6)
Al8—Al23—Al18^xv^	62.41 (5)	Al52^i^—Al66—Al53	89.32 (6)
Al32^vi^—Al23—Al18^xv^	152.60 (7)	Al49—Al66—Al53	89.43 (6)
Al7—Al23—Al18^xv^	93.58 (6)	Al54^i^—Al66—Al53	58.53 (5)
Cr7^vi^—Al23—Al20^vi^	53.29 (4)	Al57^i^—Al66—Al53	58.61 (5)
Cr9^xv^—Al23—Al20^vi^	112.02 (6)	Al51^i^—Al66—Al53	145.79 (7)
Al16^xv^—Al23—Al20^vi^	150.42 (7)	Al50—Al66—Al53	145.96 (7)
Al24—Al23—Al20^vi^	107.80 (6)	Cr2^i^—Al66—Al48	115.91 (6)
Al34^iv^—Al23—Al20^vi^	90.91 (6)	Cr1—Al66—Al48	55.67 (4)
Al12^vi^—Al23—Al20^vi^	58.20 (4)	Al55^i^—Al66—Al48	147.07 (7)
Al40^xv^—Al23—Al20^vi^	89.32 (6)	Al56—Al66—Al48	147.13 (7)
Al8—Al23—Al20^vi^	108.53 (6)	Al52^i^—Al66—Al48	59.49 (5)
Al32^vi^—Al23—Al20^vi^	58.91 (5)	Al49—Al66—Al48	59.59 (5)
Al7—Al23—Al20^vi^	58.32 (5)	Al54^i^—Al66—Al48	90.06 (6)
Al18^xv^—Al23—Al20^vi^	148.03 (7)	Al57^i^—Al66—Al48	90.16 (6)
Al2—Al24—Al23	103.73 (6)	Al51^i^—Al66—Al48	112.84 (6)
Al2—Al24—Cr5	57.22 (4)	Al50—Al66—Al48	112.95 (6)
Al23—Al24—Cr5	103.28 (6)	Al53—Al66—Al48	60.53 (5)
Al2—Al24—Cr7^vi^	57.02 (4)		

Symmetry codes: (i) −*x*+1/2, −*y*+1/2, −*z*+1; (ii) −*x*+1, *y*, −*z*+1/2; (iii) *x*+1/2, −*y*+1/2, *z*−1/2; (iv) −*x*, *y*, −*z*+1/2; (v) *x*+1/2, *y*−1/2, *z*; (vi) −*x*, −*y*, −*z*+1; (vii) −*x*, −*y*+1, −*z*+1; (viii) −*x*+1/2, *y*+1/2, −*z*+1/2; (ix) *x*, −*y*, *z*+1/2; (x) *x*−1/2, −*y*+1/2, *z*+1/2; (xi) −*x*, *y*, −*z*+3/2; (xii) *x*, −*y*+1, *z*+1/2; (xiii) −*x*−1/2, −*y*+1/2, −*z*+1; (xiv) *x*−1/2, *y*+1/2, *z*; (xv) *x*, −*y*, *z*−1/2; (xvi) *x*−1/2, −*y*+1/2, *z*−1/2; (xvii) −*x*+1/2, −*y*+1/2, −*z*; (xviii) −*x*+1/2, *y*−1/2, −*z*+1/2; (xix) *x*+1/2, −*y*+1/2, *z*+1/2.

## References

[bb1] Bendersky, L. A., Roth, R. S., Ramon, J. T. & Shechtman, D. (1991). *Metall. Trans. A*, **22**, 5–10.

[bb2] Bradley, A. J. & Lu, S. S. (1937). *J. Inst. Met.* **60**, 319–337.

[bb3] Brandenburg, K. & Putz, H. (2017). *DIAMOND*. Crystal Impact GbR, Bonn, Germany.

[bb4] Bruker (2015). *APEX3*, *SAINT* and *SADABS*, Bruker AXS Inc. Madison, Wisconsin, USA.

[bb5] Cao, B. B. & Kuo, K. H. (2008*a*). *J. Alloys Compd.* **458**, 238–247.

[bb6] Cao, B. B. & Kuo, K. H. (2008*b*). *J. Chin. Electr. Microsc. Soc*, **27**, 179–184.

[bb7] Inoue, A., Kimura, H. & Masumoto, T. (1987). *J. Mater. Sci.* **22**, 1758–1768.

[bb8] Lilienfeld, D. A., Nastasi, M., Johnson, H. H., Ast, D. G. & Mayer, J. W. (1986). *J. Mater. Res.* **1**, 237–242.

[bb9] Little, K. (1954). *J. Inst. Met.* **82**, 463.

[bb10] Shechtman, D., Blech, I., Gratias, D. & Cahn, J. W. (1984). *Phys. Rev. Lett.* **53**, 1951–1953.

[bb11] Sheldrick, G. M. (2015*a*). *Acta Cryst.* A**71**, 3–8.

[bb12] Sheldrick, G. M. (2015*b*). *Acta Cryst.* C**71**, 3–8.

[bb13] Swamy, V. T., Ranganathan, S. & Chattopadhyay, K. (1989). *J. Mater. Res.* **4**, 539–551.

[bb14] Westrip, S. P. (2010). *J. Appl. Cryst.* **43**, 920–925.

[bb15] Xia, Z., Liu, C. & Fan, C. Z. (2018). *IUCrData*, **3**, x180593.

[bb16] Zhang, H., Wang, D. H. & Kuo, K. H. (1988). *Phys. Rev. B*, **37**, 6220–6225. 10.1103/physrevb.37.6220 9943858

