# Supplementary material for: Crystal structure of Al2.95Cr0.59, a phase closely related to the η-phase in the binary Al–Cr system
Source: IUCrdata. 2020 Oct 27;5(Pt 10):x201412. doi: 10.1107/S2414314620014121 (PMC9462158; doi:10.1107/S2414314620014121)
Supplement: Supplementary file 3 [file x-05-x201412-sup3.pdf]

SUPPLEMENTARY MATERIAL

Crystal structure of $Al_{2.95}Cr_{0.59}$, a phase related to the η-phase in the Al---Cr system

Xu Geng, Bin Wen and Changzeng Fan*

State Key Laboratory of Metastable Materials Science and Technology, Yanshan University, Qinhuangdao 066004, P.R. China

*Correspondence email: chzfan@ysu.edu.cn

Table S1. Comparison of the atom coordinates for the *η*-phase [Cao, B. B. & Kuo, K. H. (2008). *J. Alloys Compd.* **458**, 30, 319-337] (with white background) and the η′-phase [this work] (with grey background).

Label	Site	x	y	z	Occ.	B_{equ}
**Cr1**	4*d*	0.25000	0.25000	0.50000	1	0.0053(1)
**Cr1**	4*c*	0.250000	0.250000	0.500000	1	0.0041(3)
**Cr2**	8*f*	0.44728(2)	0.24899(1)	0.30274(2)	1	0.0047(1)
**Cr2**	8*f*	0.44734(4)	0.24848(2)	0.30280(4)	1	0.00366(19)
**Cr3**	4*e*	0.00000	0.39562(2)	0.25000	1	0.0045(1)
**Cr3**	4*e*	0.000000	0.39588(3)	0.250000	1	0.0038(3)
**Cr4**	4*e*	0.00000	0.10540(2)	0.25000	1	0.0045(1)
**Cr4**	4*e*	0.000000	0.10561(3)	0.250000	1	0.0035(3)
**Cr5**	8*f*	0.24987(2)	-0.03623(1)	0.49969(2)	1	0.0048(1)
**Cr5**	8*f*	0.24978(4)	-0.03633(2)	0.49964(4)	1	0.00377(19)
**Cr6**	8*f*	0.16284(2)	0.48619(1)	0.41386(2)	1	0.0047(1)
**Cr6**	8*f*	0.16243(4)	0.48613(2)	0.41394(4)	1	0.00351(18)
**Cr7**	8*f*	-0.16431(2)	-0.01338(1)	0.58668(2)	1	0.0046(1)
**Cr7**	8*f*	-0.16469(4)	-0.01328(2)	0.58691(4)	1	0.00311(18)
**Cr8**	8*f*	0.40203(2)	0.03972(1)	0.34717(2)	1	0.0056(1)
**Cr8**	8*f*	0.40155(4)	0.04038(3)	0.34708(4)	1	0.00335(19)
**Cr9**	8*f*	0.09619(2)	0.03628(1)	0.65293(2)	1	0.0054(1)
**Cr9**	8*f*	0.09583(4)	0.03550(2)	0.65295(4)	1	0.00351(19)
**Cr10**	8*f*	0.15625(2)	0.17676(1)	0.10093(2)	1	0.0078(1)
**Cr10**	8*f*	0.15775(4)	0.17684(2)	0.10249(4)	1	0.00398(18)
**Cr11**	8*f*	0.14887(2)	0.32370(1)	0.09399(2)	1	0.0077(1)
**Cr11**	8*f*	0.14733(4)	0.32385(2)	0.09261(4)	1	0.00369(18)
**Cr12/Al**	8*f*	0.18594(2)	0.29768(1)	0.23289(2)	0.8291/0.1709	0.0077(1)
**Cr12**	8*f*	0.18525(4)	0.29801(2)	0.23235(4)	1	0.00487(19)
**Cr13/Al**	8*f*	0.14815(2)	0.10965(1)	0.60156(2)	0.8291/0.1709	0.0060(1)
**Cr13**	8*f*	0.14806(4)	0.10915(2)	0.60130(4)	1	0.00353(18)
**Cr14/Al**	8*f*	-0.01719(2)	0.20251(1)	0.43598(2)	0.8291/0.1709	0.0078(1)

**Cr14**	8*f*	-0.01777(4)	0.20238(2)	0.43544(4)	1	0.00468(19)
**Al1**	4*e*	0.00000	0.45807(3)	0.750000	1	0.0082(2)
**Al1**	4*e*	0.000000	0.45786(7)	0.750000	1	0.0077(5)
**Al2**	8*f*	0.30124(4)	0.03764(2)	0.44881(4)	1	0.0059(1)
**Al2**	8*f*	0.30099(8)	0.03789(5)	0.44894(7)	1	0.0038(3)
**Al3**	8*f*	-0.19380(4)	0.10281(2)	0.44208(4)	1	0.0079(1)
**Al3**	8*f*	-0.19430(8)	0.10296(5)	0.44181(8)	1	0.0064(3)
**Al4**	8*f*	0.05717(4)	0.25030(2)	0.30717(4)	1	0.0069(1)
**Al4**	8*f*	0.05724(8)	0.25039(4)	0.30708(8)	0.899(5)	0.0092(5)
**Cr15**	8*f*	0.05724(8)	0.25039(4)	0.30708(8)	0.101(5)	0.0092(5)
**Al5**	8*f*	0.19798(4)	0.03602(2)	0.55203(4)	1	0.0055(1)
**Al5**	8*f*	0.19798(8)	0.03557(5)	0.55224(7)	0.984(4)	0.0043(5)
**Cr16**	8*f*	0.19798(8)	0.03557(5)	0.55224(7)	0.016(4)	0.0043(5)
**Al6**	8*f*	-0.19169(4)	0.39774(2)	0.44344(4)	1	0.0079(1)
**Al6**	8*f*	-0.19137(8)	0.39765(5)	0.44365(8)	1	0.0061(3)
**Al7**	8*f*	0.08899(4)	0.06808(2)	0.33983(4)	1	0.0082(1)
**Al7**	8*f*	0.08860(8)	0.06822(5)	0.34072(8)	1	0.0068(3)
**Al8**	8*f*	0.24886(4)	0.06012(3)	0.30761(4)	1	0.0104(1)
**Al8**	8*f*	0.24834(8)	0.05992(5)	0.30739(8)	1	0.0096(4)
**Al9**	8*f*	0.15822(4)	0.11360(3)	0.21244(5)	1	0.0119(1)
**Al9**	8*f*	0.15861(8)	0.11356(5)	0.21280(8)	1	0.0098(4)
**Al10**	8*f*	-0.25069(4)	0.18467(2)	0.49958(4)	1	0.0078(1)
**Al10**	8*f*	-0.25107(8)	0.18478(5)	0.49931(8)	1	0.0069(4)
**Al11**	8*f*	0.10215(4)	0.25020(2)	0.14782(4)	1	0.0057(1)
**Al11**	8*f*	0.10237(8)	0.25033(5)	0.14778(8)	0.977(5)	0.0056(5)
**Cr17**	8*f*	0.10237(8)	0.25033(5)	0.14778(8)	0.023(5)	0.0056(5)
**Al12**	4*e*	0.00000	0.04112(3)	0.75000	1	0.0074(2)
**Al12**	4*e*	0.000000	0.04101(7)	0.750000	1	0.0053(5)
**Al13**	8*f*	-0.04935(4)	0.32267(2)	0.30270(4)	1	0.0058(1)
**Al13**	8*f*	-0.04869(7)	0.32273(5)	0.30330(7)	0.946(4)	0.0061(5)
**Cr18**	8*f*	-0.04869(7)	0.32273(5)	0.30330(7)	0.054(4)	0.0061(5)
**Al14**	8*f*	0.05857(4)	0.05754(2)	0.50244(4)	1	0.0090(1)
**Al14**	8*f*	0.05907(8)	0.05710(5)	0.50340(8)	1	0.0066(3)
**Al15**	8*f*	-0.05272(4)	0.17799(2)	0.29932(4)	1	0.0058(1)
**Al15**	8*f*	-0.05333(7)	0.17819(4)	0.29867(7)	0.945(4)	0.0056(4)
**Cr19**	8*f*	-0.05333(7)	0.17819(4)	0.29867(7)	0.055(4)	0.0056(4)
**Al16**	8*f*	0.24783(4)	0.05762(2)	0.69111(4)	1	0.0094(1)
**Al16**	8*f*	0.24698(8)	0.05713(5)	0.69112(8)	1	0.0067(3)
**Al17**	8*f*	0.05785(4)	0.43980(3)	0.49861(4)	1	0.0104(1)
**Al17**	8*f*	0.05822(8)	0.44014(5)	0.49793(8)	1	0.0097(4)
**Al18**	8*f*	0.21337(4)	-0.02952(2)	0.64986(4)	1	0.0084(1)
**Al18**	8*f*	0.21340(8)	-0.02979(5)	0.64991(8)	1	0.0063(3)
**Al19**	8*f*	0.09967(4)	-0.03027(2)	0.53646(4)	1	0.0080(1)
**Al19**	8*f*	0.09959(8)	-0.03082(5)	0.53662(8)	1	0.0061(3)
**Al20**	8*f*	-0.03161(4)	0.01318(2)	0.60043(4)	1	0.0073(1)
**Al20**	8*f*	-0.03199(8)	0.01301(5)	0.60072(8)	1	0.0059(3)
**Al21**	8*f*	0.13625(4)	0.24905(2)	0.00131(4)	1	0.0087(1)
**Al21**	8*f*	0.13623(8)	0.24868(5)	0.00158(8)	1	0.0073(4)
**Al22**	8*f*	0.24863(4)	0.25102(2)	0.11375(4)	1	0.0087(1)
**Al22**	8*f*	0.24848(8)	0.25145(5)	0.11385(8)	1	0.0071(3)
**Al23**	8*f*	0.14974(4)	-0.01311(2)	0.28085(4)	1	0.0076(1)
**Al23**	8*f*	0.14938(8)	-0.01304(5)	0.28092(8)	1	0.0063(3)
**Al24**	8*f*	0.28605(4)	-0.02861(2)	0.35013(4)	1	0.0086(1)
**Al24**	8*f*	0.28573(8)	-0.02881(5)	0.35037(8)	1	0.0075(4)

**Al25**	8*f*	0.28761(4)	0.34274(2)	0.15087(4)	1	0.0094(1)
**Al25**	8*f*	0.28704(8)	0.34248(5)	0.15098(8)	1	0.0079(3)
**Al26**	8*f*	-0.10058(4)	0.47215(2)	0.46375(4)	1	0.0081(1)
**Al26**	8*f*	-0.10093(8)	0.47223(5)	0.46366(8)	1	0.0068(3)
**Al27**	8*f*	-0.03007(4)	0.48631(2)	0.59896(4)	1	0.0078(1)
**Al27**	8*f*	-0.02969(8)	0.48609(5)	0.59881(8)	1	0.0071(3)
**Al28**	8*f*	0.30396(5)	0.10035(2)	0.56393(5)	1	0.0129(2)
**Al28**	8*f*	0.30215(8)	0.10031(5)	0.56536(8)	1	0.0064(3)
**Al29**	8*f*	0.18634(5)	0.10053(2)	0.44636(5)	1	0.0126(2)
**Al29**	8*f*	0.18487(8)	0.10057(5)	0.44794(8)	1	0.0062(3)
**Al30**	8*f*	0.34916(4)	0.01378(2)	0.21915(4)	1	0.0078(1)
**Al30**	8*f*	0.34886(8)	0.01406(5)	0.21925(8)	1	0.0063(3)
**Al31**	8*f*	0.09764(5)	0.34224(3)	0.33731(5)	1	0.0136(2)
**Al31**	8*f*	0.09927(8)	0.34223(5)	0.33571(8)	1	0.0073(3)
**Al32**	8*f*	-0.15422(4)	0.07574(2)	0.5962(4)	1	0.0088(1)
**Al32**	8*f*	-0.15423(8)	0.07608(5)	0.59617(8)	1	0.0067(3)
**Al33**	8*f*	0.08733(5)	0.15829(3)	0.34765(5)	1	0.0135(2)
**Al33**	8*f*	0.08565(8)	0.15864(5)	0.34924(8)	1	0.0067(3)
**Al34**	8*f*	-0.06096(4)	0.04095(2)	0.31068(4)	1	0.0078(1)
**Al34**	8*f*	-0.06083(8)	0.04125(5)	0.31042(8)	1	0.0063(3)
**Al35**	8*f*	-0.06176(4)	0.46037(2)	0.31202(4)	1	0.0078(1)
**Al35**	8*f*	-0.06187(8)	0.46077(5)	0.31249(8)	1	0.0060(3)
**Al36**	8*f*	0.08919(4)	0.43284(2)	0.33832(4)	1	0.0083(1)
**Al36**	8*f*	0.08975(8)	0.43312(5)	0.33737(8)	1	0.0068(3)
**Al37**	8*f*	0.04317(4)	0.11460(2)	0.09684(5)	1	0.0104(1)
**Al37**	8*f*	0.04419(8)	0.11508(5)	0.09782(8)	1	0.0073(3)
**Al38**	8*f*	-0.00248(4)	0.10295(2)	0.63318(4)	1	0.0089(1)
**Al38**	8*f*	-0.00194(8)	0.10311(5)	0.63352(8)	1	0.0068(3)
**Al39**	8*f*	-0.15351(4)	0.42563(2)	0.59608(4)	1	0.0083(1)
**Al39**	8*f*	-0.15352(8)	0.42581(5)	0.59628(8)	1	0.0066(3)
**Al40**	8*f*	0.11697(4)	0.10282(2)	0.75211(4)	1	0.0089(1)
**Al40**	8*f*	0.11673(8)	0.10287(5)	0.75126(8)	1	0.0071(3)
**Al41**	8*f*	0.09923(4)	0.15754(2)	-0.03785(4)	1	0.0095(1)
**Al41**	8*f*	0.09933(8)	0.15775(5)	-0.03738(8)	1	0.0075(3)
**Al42**	8*f*	0.50458(4)	0.10243(2)	0.36808(5)	1	0.0097(1)
**Al42**	8*f*	0.50498(8)	0.10226(5)	0.36801(8)	1	0.0085(4)
**Al43**	8*f*	0.38203(4)	0.10259(2)	0.24508(4)	1	0.0094(1)
**Al43**	8*f*	0.38188(8)	0.10254(5)	0.24457(8)	1	0.0076(3)
**Al44**	8*f*	-0.03734(4)	0.38711(3)	0.40809(5)	1	0.0115(1)
**Al44**	8*f*	-0.03745(8)	0.38746(5)	0.40781(8)	1	0.0105(4)
**Al45**	8*f*	0.15303(4)	0.38604(2)	0.20707(5)	1	0.0105(1)
**Al45**	8*f*	0.15248(8)	0.38578(5)	0.20640(8)	1	0.0073(3)
**Al46**	8*f*	0.29056(4)	0.15695(2)	0.15097(4)	1	0.0096(1)
**Al46**	8*f*	0.29115(8)	0.15696(5)	0.15089(8)	1	0.0080(3)
**Al47**	8*f*	0.09885(4)	0.34330(2)	-0.04034(4)	1	0.0098(1)
**Al47**	8*f*	0.09882(8)	0.34335(5)	-0.04088(8)	1	0.0079(3)
**Al48A**	8*f*	0.20788(5)	0.17332(3)	0.54201(5)	0.8291	0.0081(2)
**Al48B**	8*f*	0.2081(2)	0.32659(14)	0.5420(3)	0.1709	0.0081(2)
**Al48**	8*f*	0.20769(8)	0.17339(5)	0.54219(8)	1	0.0072(3)
**Al49A**	8*f*	0.11266(5)	0.22868(3)	0.45304(5)	0.8291	0.0085(2)
**Al49B**	8*f*	0.2032(2)	0.22884(14)	0.3627(3)	0.1709	0.0085(2)
**Al49**	8*f*	0.11258(8)	0.22881(5)	0.45288(8)	1	0.0075(3)
**Al50A**	8*f*	0.13590(5)	0.32294(3)	0.48413(5)	0.8291	0.0123(2)
**Al50B**	8*f*	0.2660(3)	0.17694(15)	0.6137(3)	0.1709	0.0123(2)
**Al50**	8*f*	0.13633(9)	0.32293(5)	0.48425(8)	1	0.0144(4)
**Al51A**	8*f*	0.23410(5)	0.17724(3)	0.38596(6)	0.8291	0.0122(2)
**Al51B**	8*f*	0.1364(3)	0.17782(15)	0.4841(3)	0.1709	0.0122(2)

**Al51**	8*f*	0.23406(8)	0.17736(5)	0.38604(9)	1	0.0143(4)
**Al52A**	8*f*	0.20296(5)	0.27147(3)	0.36270(5)	0.8291	0.0084(2)
**Al52B**	8*f*	0.1128(2)	0.27190(14)	0.4535(2)	0.1709	0.0084(2)
**Al52**	8*f*	0.20329(8)	0.27139(5)	0.36267(8)	1	0.0079(3)
**Al53A**	8*f*	0.09217(5)	0.17541(3)	0.65782(5)	0.8291	0.0070(2)
**Al53B**	8*f*	0.4076(2)	0.17549(13)	0.3426(2)	0.1709	0.0070(2)
**Al53**	8*f*	0.09196(8)	0.17539(5)	0.65788(8)	1	0.0063(3)
**Al54A**	8*f*	0.31646(5)	0.27081(3)	0.25058(5)	0.8291	0.0069(2)
**Al54B**	8*f*	0.4995(2)	0.22898(13)	0.4338(2)	0.1709	0.0069(2)
**Al54**	8*f*	0.31655(8)	0.27079(5)	0.25076(8)	1	0.0061(3)
**Al55A**	8*f*	0.34778(5)	0.18345(3)	0.28628(5)	0.8291	0.0075(2)
**Al55B**	8*f*	0.0361(2)	0.18346(13)	0.5972(2)	0.1709	0.0075(2)
**Al55**	8*f*	0.34771(8)	0.18353(5)	0.28639(8)	1	0.0063(3)
**Al56A**	8*f*	0.03623(5)	0.31659(3)	0.59787(5)	0.8291	0.0074(2)
**Al56B**	8*f*	0.1524(2)	0.18327(13)	0.7138(2)	0.1709	0.0074(2)
**Al56**	8f	0.03640(8)	0.31665(5)	0.59768(8)	1	0.0060(3)
**Al57A**	8*f*	0.49938(5)	0.27070(3)	0.43351(5)	0.8291	0.0070(2)
**Al57B**	8*f*	0.3164(2)	0.22937(13)	0.2510(2)	0.1709	0.0070(2)
**Al57**	8*f*	0.49945(8)	0.27059(5)	0.43355(8)	1	0.0066(3)
**Al58A**	8*f*	-0.09387(5)	0.27605(3)	0.65611(5)	0.8291	0.0083(2)
**Al58B**	8*f*	-0.0939(2)	0.22408(14)	0.6564(2)	0.1709	0.0083(2)
**Al58**	8*f*	-0.09387(8)	0.27614(5)	0.65609(8)	1	0.0077(3)
**Al59A**	8*f*	-0.05811(5)	0.18589(3)	0.69191(5)	0.8291	0.0082(2)
**Al59B**	8*f*	-0.0578(2)	0.3143(14)	0.6918(2)	0.1709	0.0082(2)
**Al59**	8*f*	-0.05813(8)	0.18594(5)	0.69193(8)	1	0.0072(3)
**Al60A**	4*e*	0.00000	0.33017(4)	0.75000	0.8291	0.0084(2)
**Al60B**	4*e*	0.00000	0.1701(2)	0.75000	0.1709	0.0084(2)
**Al60**	4*e*	0.000000	0.33001(7)	0.750000	1	0.0072(5)
**Al61A**	8*f*	0.05845(5)	0.14680(3)	0.50766(5)	0.8291	0.0072(2)
**Al61B**	8*f*	0.0589(2)	0.35343(13)	0.5073(2)	0.1709	0.0072(2)
**Al61**	8*f*	0.05876(8)	0.14688(5)	0.50765(8)	1	0.0071(3)
**Al62A**	8*f*	0.25769(5)	0.35331(3)	0.30848(5)	0.8291	0.0071(2)
**Al62B**	8*f*	0.2570(2)	0.14742(13)	0.3089(2)	0.1709	0.0071(2)
**Al62**	8*f*	0.25770(8)	0.35334(5)	0.30867(8)	1	0.0063(3)
**Al63/Cr**	8*f*	0.35083(4)	0.11486(2)	0.39853(4)	0.8291/0.1709	Al63/Cr
**Al63**	8*f*	0.35041(8)	0.11692(5)	0.39887(8)	1	0.0064(3)
**Al64/Cr**	8*f*	0.19284(4)	0.20615(2)	0.24032(4)	0.8291/0.1709	Al64/Cr
**Al64**	8*f*	0.19528(8)	0.20754(5)	0.24318(8)	1	0.0062(3)
**Al65/Cr**	8*f*	-0.00959(4)	0.29416(2)	0.44279(4)	0.8291/0.1709	0.0150(1)
**Al65**	8*f*	-0.00686(8)	0.29306(5)	0.44513(8)	1	0.0070(3)
**Al66**	8*f*	0.15073(4)	0.25530(3)	0.59921(4)	1	0.0144(2)
**Al66**	8*f*	0.15067(8)	0.25680(5)	0.59927(8)	1	0.0070(3)

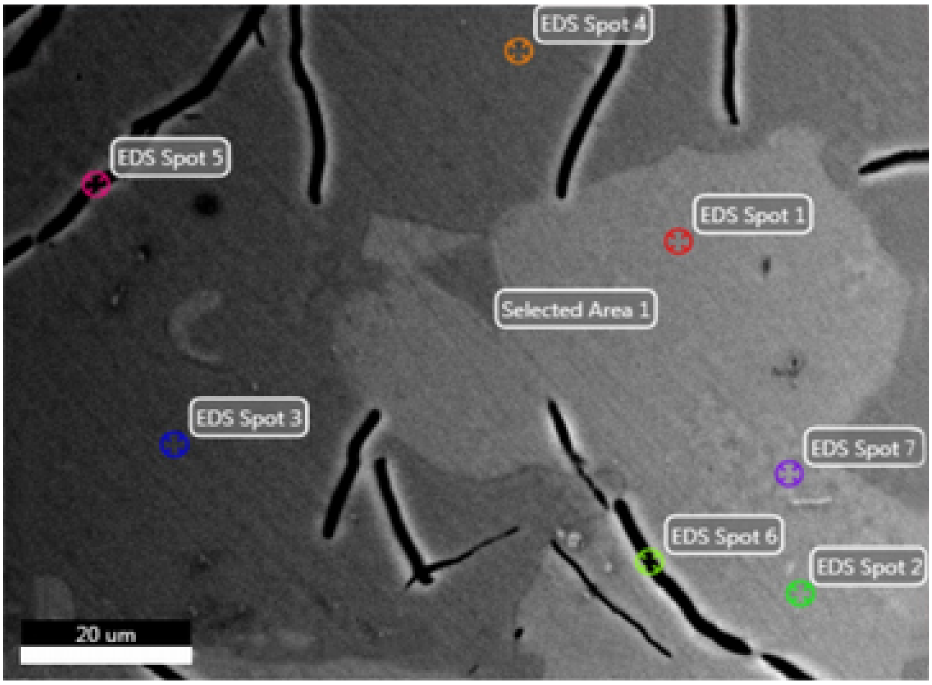

Figure S1. Scanning electron microscope (SEM) micrographs and energy dispersive X-ray spectroscopy (EDS) analysis of the sample prepared by HPS; single crystal fragments for X-ray diffraction studies were selected from this piece. EDS analysis was performed on different sites (spot 1-7 and area 1) and results are listed in Table S1.

Table S1. Chemical compositions of the sample as revealed by EDS analysis (spots 1-7 and area 1 are denoted in Figure S1)

	**Spot 1**	**Spot 2**	**Spot 3**	**Spot 4**	**Spot 5**	**Spot 6**	**Spot 7**
**x(Al)/%**	83.14(±3.89)	83.24(±3.88)	86.00(±3.56)	86.26(±3.52)	86.19(±3.53)	83.28(±3.88)	83.12(±3.89)
**x(Cr)/ %**	16.86(±2.25)	16.76(±2.23)	14.00(±2.40)	13.74(±2.39)	13.81(±2.41)	16.72(±2.26)	16.88(±2.25)
